# Structural brain alterations in anorexia nervosa: a global brain volume and anatomical likelihood estimation (ALE) meta-analysis combined with a functional decoding approach

**DOI:** 10.1016/j.nicl.2026.103950

**Published:** 2026-01-18

**Authors:** Lara Keller, Leon D. Lotter, Claudia R. Eickhoff, Simon B. Eickhoff, Katharina Otten, Beate Herpertz-Dahlmann, Jochen Seitz

**Affiliations:** a Department of Child and Adolescent Psychiatry, Psychosomatics and Psychotherapy, LVR University Hospital Essen, University of Duisburg-Essen, Wickenburgstr. 21, Essen 45147, Germany; b Child Neuropsychology Section, Department of Child and Adolescent Psychiatry, Psychosomatics and Psychotherapy, University Hospital RWTH Aachen, Neuenhofer Weg 21, Aachen 52074, Germany; c West German Center for Child and Adolescent Health, Partner Site Essen, Germany; d Institute of Neuroscience and Medicine, Brain & Behaviour (INM-7), Jülich Research Centre, Wilhelm-Johnen-Straße, Jülich 52428, Germany; e Institute of Systems Neuroscience, Medical Faculty, Heinrich Heine University Düsseldorf, Moorenstrasse 5, Düsseldorf 40225, Germany; f Max Planck School of Cognition, Stephanstrasse 1A, Leipzig 04103, Germany; g Institute of Neuroscience and Medicine Research, Structural and functional organisation of the brain (INM-1), Jülich Research Center, Wilhelm-Johnen-Straße, Jülich 52428, Germany; h Institute of Clinical Neuroscience and Medical Psychology, Medical Faculty, Heinrich Heine University, Moorenstrasse 5, Düsseldorf 40225, Germany; i Department of Child and Adolescent Psychiatry, Psychosomatics and Psychotherapy, University Hospital RWTH Aachen, Neuenhofer Weg 21, Aachen 52074, Germany; j West German Center for Child and Adolescent Health, Partner Site Aachen, Germany

**Keywords:** Anorexia nervosa, Meta-analysis, Brain structure, Anatomical likelihood estimation, Functional decoding, Brain volume loss, Clinical implications

## Abstract

Substantial brain volume loss is well-documented during acute anorexia nervosa (AN); however, longitudinal outcomes are unclear. Our comprehensive meta-analysis investigated global and regional structural brain alterations in adult and adolescent individuals with AN by extracting reported brain volume scores and neuroimaging coordinates from the literature. Results showed significant global brain volume reductions in gray matter (GM), white matter (WM), and increases in cerebrospinal fluid (CSF) in acute AN (N = 1130 patients; N = 40 papers), gradually improving upon weight rehabilitation. However, even after 1.5 years of recovery, significantly lower global GM volume compared to healthy controls was found (N = 232 patients; N = 12 papers). Regarding potential regional changes, our search identified 35 eligible papers with neuroimaging coordinates for 412 foci as input for our anatomical likelihood estimation (ALE) analyses. The results revealed widespread reductions of GM volume and cortical thickness, but notably also identified consistently affected brain regions including the cingulate gyrus, precentral gyrus, and precuneus. Spatial colocalization analyses using the Neurosynth data base indicated brain areas associated with eating, food, threat, and reinforcement to be relatively preserved. The findings of our meta-analysis contribute to a better understanding of the underlying pathophysiology of AN, the time course and residuals of brain structural alterations during recovery and clinical implications potentially relevant for more-targeted treatment options.

## Highlights

• Acute AN shows a reduction of –4.79% gray matter and –2.48% white matter with numerically larger reductions in adolescents.
• Brain volumes improve with weight restoration but remain reduced up to 1.5 years post‑recovery.
• Anatomical likelihood estimation (ALE) indicates widespread gray matter and cortical thickness reductions.
• Key areas affected in AN include the cingulate gyrus, precentral gyrus, and precuneus.
• Preserved regions colocalize with brain areas for eating, food cues, threat, and reinforcement (FDR-corrected).

## 1 Introduction

The core symptoms of anorexia nervosa (AN) comprise significantly low body weight, intense fear of gaining weight and body image disturbances (American PsychiatricAssociation, 2013). Furthermore, AN induced semi-starvation can lead to serious somatic alterations including amenorrhea, loss of bone mineral density, and hormonal imbalance including low estrogen and leptin concentrations and elevated cortisol levels (Haines, 2023, Skowron et al., 2023). Despite ongoing advances in research and continuous improvements in the treatment of AN in recent years, AN remains a severe psychiatric disorder with a high mortality rate (Auger et al., 2021, van Eeden et al., 2021). Studies show that the multimodal treatment approach, including nutritional rehabilitation, treatment of medical problems plus family and individual psychotherapy which is currently considered the gold standard in treating AN (Herpertz-Dahlmann et al., 2015), is only moderately effective with a substantial risk for relapses and chronicity (Dobrescu et al., 2020, Solmi et al., 2024). This underlines the clear need for a better understanding of AN through integrating knowledge from both clinical and biological perspectives to gain deeper insights into underlying pathomechanisms and subsequently improve its treatment through specific research endeavors and targeted therapeutic approaches.

In this context, structural brain alterations are an important research target as they could help explain clinical psychopathology of AN. Numerous prior single neuroimaging studies and meta-analyses have demonstrated a clearly altered brain morphology among patients with AN in the acute stage of disease, characterized by a significant reduction in cortical thickness (CT), gray matter (GM) and white matter (WM) volume, as well as an increase in cerebrospinal fluid (CSF) space volume that could be predictive for clinical outcomes (see e.g., Bahnsen et al., 2022, Bracké et al., 2023, Kappou et al., 2021, Seitz et al., 2018, Su et al., 2021, Vidal et al., 2021, Walton et al., 2022, Zhang et al., 2018). Even though this brain volume loss in acute AN is well-documented, overall, the brain structural changes in AN are not yet fully understood and several questions remain unanswered, to which the present meta-analysis seeks to contribute: Most studies have found that the brains of patients with AN seems to recover quickly during weight gain, indicating the morphological alterations as state markers of the disease and substantially reversible after weight recovery (e.g., Bomba et al., 2015, Castro-Fornieles et al., 2009, Lázaro et al., 2013, Mainz et al., 2012). However, there is a lack of consensus regarding differences between recovered patients and healthy controls (HCs) after a longer-term follow-up. While some studies have observed sustained global and regional brain volume differences in recovered patients (e.g., Friederich et al., 2012, Joos et al., 2011a, Mühlau et al., 2007, Oliva et al., 2020, Roberto et al., 2011, Scaife et al., 2017), others have not (e.g., Bang et al., 2016, Cascino et al., 2020, Favaro et al., 2015, Nickel et al., 2018; see Vidal et al., 2021 for a systematic review). One previous meta-analysis did not find robust evidence for persisting volume reductions (Bahnsen et al., 2022), whereas others found numerical residuals towards a continuous brain volume reduction, although statistically non-significant (Seitz et al., 2014, Seitz et al., 2016, Seitz et al., 2018). Enduring brain changes even after recovery, however, could be relevant for psychopathology and the prognosis of disease. The drastically altered brain morphology in the acute phase of AN could help explain neuropsychological deficits (Miles et al., 2020, Stedal et al., 2021), while persisting alterations after recovery could contribute to residual symptoms, relapses, and the oftentimes severe course of AN.

Another important question that remains unclear is whether the altered brain morphology in AN is a global phenomenon impacting the entire brain or whether specific brain regions are particularly affected and show consistent alterations across studies. A recent large-scale coordinated meta-analysis of multicenter neuroimaging data by Walton and colleagues (Walton et al., 2022) within the ENIGMA Eating Disorders framework demonstrated considerable widespread reductions in subcortical volumes and CT, with particularly affected brain regions being the thalamus and the superior and inferior parietal gyrus. As another route to investigate this, anatomical likelihood estimation (ALE) meta-analyses can be performed. They belong to the coordinate-based meta-analytic approaches providing a powerful tool for summarizing the results of neuroimaging studies, allowing for an objective and comprehensive representation of the current state of research. To date, only few coordinate-based meta-analyses have investigated structural brain alterations in AN. A first ALE meta-analysis by Titova and colleagues (Titova et al., 2013) identified clusters of significantly reduced GM volume in AN. However, it included only seven individual voxel-based morphometry (VBM) studies, which is significantly below the minimal number of required studies for performing ALE meta-analysis corresponding to a recommendation by Eickhoff and colleagues (Eickhoff et al., 2016), limiting the generalizability of results. Another ALE meta-analysis by Sader and colleagues (Sader et al., 2022) including 23 VBM studies found clusters of both significantly increased and decreased GM volume in the patient group. Furthermore, a similar coordinate-based approach called seed-based d mapping (SDM) was applied in two meta-analyses (Su et al., 2021, Zhang et al., 2018) revealing no significant increases in GM volume in AN but distinct clusters of decreased GM volume. However, outcomes vary between these meta-analyses, and they focused on GM volume only. To our knowledge, no coordinate-based meta-analysis exists to date that combines results on GM volume and CT in AN. The present meta-analysis aims to fill this gap and substantially increase the pool of included studies.

Additionally, to date no previous meta-analysis has applied functional decoding as a systematic and objective approach to examine the clinical or functional significance of regional structural brain alterations in AN. Instead, existing investigations have primarily relied on narrative, however necessarily subjective and selective interpretations of literature-based associations to infer the functions associated with affected regions identified through the meta-analyses. Addressing this gap, we applied functional decoding and spatial colocalization analyses with neurotransmitter systems to our meta-analysis, which is a novel and innovative approach in the field of research in AN. These systematic results may help to generate well-founded hypotheses about underlying neurobiological mechanisms and broaden our understanding of the functional and clinical implications of structural brain changes revealed by our ALE meta-analysis.

The main objective of the present study is thus to investigate both global and regional structural brain changes in patients with AN in the acute stage of the disease and during recovery, with a specific focus on age-related differences between adolescents and adults, considering both the influence of neurodevelopmental stage and the clinical and functional relevance of these alterations. The analyses focused exclusively on individuals diagnosed with typical or atypical anorexia nervosa, and disease stages explored were categorized as acute, short-term and longer-term recovery (defined as > 1.5 years since full remission). In addition to quantifying global GM, WM, and CSF alterations, we performed ALE meta-analyses to identify specifically affected brain regions. By integrating functional decoding and spatial colocalization analyses with neurotransmitter systems in a structured and unbiased analysis using the results from our ALE meta-analysis as input, our study extends beyond anatomical mapping and contributes potential new insights into the biological underpinnings and clinical relevance of brain volume loss in AN, enhancing the explanatory value of coordinate-based meta-analyses.

## 2 Methods

After identifying the currently available literature on brain structure in patients with AN by means of a comprehensive and systematic literature search, we applied two different meta-analytic approaches. First, a meta-analysis on global brain volume differences was performed by using total brain volume scores of GM, WM, and CSF reported in the articles. Second, Montreal Neurological Institute (MNI) or Talairach (TAL) coordinates from imaging studies in the field were extracted and served as input for a series of ALE meta-analyses on GM volume and CT (collectively referred to as GM). Afterwards, the resulting ALE maps were used for a further contextualization in form of functional decoding and spatial association analyses with neurotransmitter systems. Whenever feasible, subgroup meta-analyses were conducted between adolescent and adult patients, as well as between acutely ill individuals under care and those at different stages of recovery, given the relevance of age-related neurodevelopmental processes occurring during adolescence, the peak onset phase of AN, and the potential impact of recovery stage in the reversibility and persistence of brain alterations.

### 2.1 Literature search and study selection

This meta-analysis was performed in accordance with the Preferred Reporting Items for Systematic Reviews and Meta-Analyses (PRISMA) guidelines (Moher et al., 2009). With the aim of identifying all relevant studies investigating brain structure in AN, three data bases (Embase, PubMed, and Scopus) were searched for relevant articles. The following search criteria were used: (“anorexia nervosa” OR “anorexia” OR “eating disorder”) AND (“VBM” OR “voxel-based morphometry” OR “gray matter” OR “grey matter” OR “white matter” OR “cortical thickness” OR “cortical volume”). The search was limited to “Title and Abstract”. Additionally, reference lists of relevant articles and previous meta-analyses and reviews were screened to complete the literature search. The final literature search was conducted on January 10, 2025, and included articles published before this date.

The following criteria were applied for inclusion: (i) cross-sectional or longitudinal case-control studies examining patients with a current or past primary diagnosis of AN according to DSM-IV, DSM-5 or ICD-10, (ii) publication in a peer-reviewed journal, (iii) written in English or German, (iv) for the global brain volume meta-analysis: original MRI studies reporting brain volume measures (GM, WM and/or CSF) at whole-brain level, (v) for the ALE meta-analysis: original MRI studies reporting structural differences in a stereotactic space in three-dimensional standard coordinates (MNI or TAL). Corresponding authors were contacted to obtain further data, if details were not provided in the published articles and if it was likely that the relevant data had been assessed in the study. Exclusion criteria were as follows: (i) review articles, meta-analyses or other document types (e.g., comments, letters), (ii) PET, SPECT, and purely fMRI or DTI studies, (iii) single-case studies or case series, (iv) sample overlap with other publications (in this case, the study with the largest sample sizes was selected), (v) no HC group, (vi) only region of interest (ROI) analyses reported, (vii) data not available, i.e. relevant brain volume scores or (for the ALE-analysis only) MNI/ TAL coordinates were not reported or it was impossible to obtain this information (e.g., full-text not found or e-mail request not answered).

**Fig. 1:**
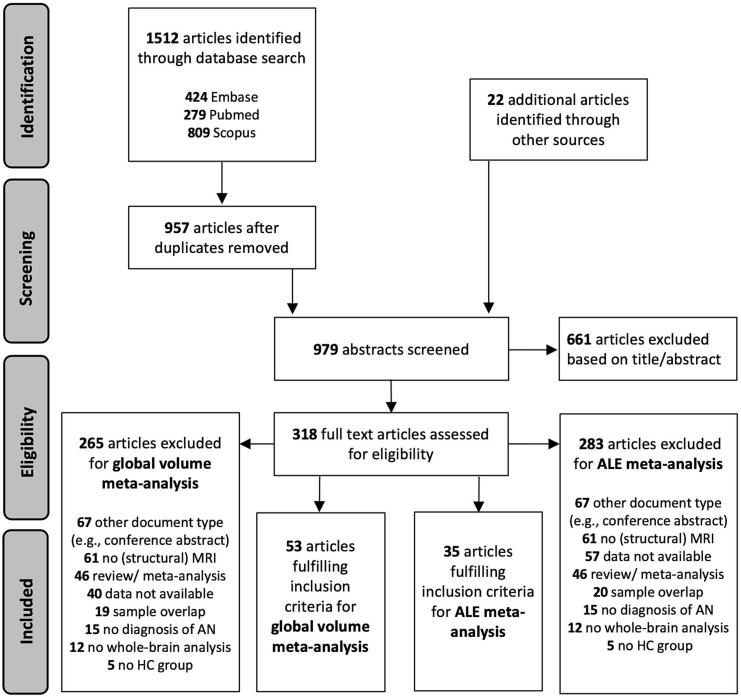
PRISMA flowchart displaying the study selection process including information about excluded studies and respective reasons. *ALE* Anatomical likelihood estimation; *AN* Anorexia nervosa; *HC*, Healthy controls.

Study selection followed a stepwise approach: Initially, duplicates were removed in the identification phase. Next, titles and abstracts of all articles identified through the literature search were screened for eligibility in the screening phase. In this step, no fMRI or DTI articles were excluded to ensure an evaluation of full texts of apparently irrelevant papers which might however include analyses of brain structure. Finally, full texts of potentially relevant articles were obtained and reviewed for eligibility based on the predefined inclusion and exclusion criteria. Fig. 1 shows a PRISMA flowchart (Moher et al., 2009) providing an overview of each step in the study selection process.

### 2.2 Data extraction and risk of bias assessment

As outcome measures for the global brain volume meta-analysis, reported scores of GM, WM, CSF, and total intracranial volume (ICV) together with their standard deviation were extracted. The patient group was subdivided into different groups based on the stadium of disease: patients with acute AN (AN_acute_), patients after weight-restoration and short-term weight recovery (AN_short-rec_), and patients being longer-term recovered (continued average duration of recovery > 1.5 years, AN_longer-rec_). In all studies reporting on longer-term recovery, key criterion for recovery was a maintained stable body weight within a healthy range. Most studies established additional criteria for recovery (e.g., regular menstruation, no binging or purging behavior, no restrictive eating patterns), however, studies differed in their exact definition. Data extraction was performed separately for patients with AN and respective HCs. For recovered patients, the mean recovery length was also recorded. Additionally, sample size, age group (adolescents vs. adults), mean age, mean BMI, mean illness duration (plus respective standard deviation), and the analysis method used (volume-based or surface-based) were extracted.

For the ALE meta-analyses, reported TAL or MNI standard coordinates represented the primary outcome measures and were extracted for all experiments from all included papers. According to the definition of Eickhoff and colleagues (Eickhoff et al., 2016), in this meta-analysis the term “paper” is used to refer to the entire published article, whereas the term “experiment” refers to distinct comparisons reported in the papers (e.g., one paper could report comparisons on both CT and GM volume, thus containing two experiments). Furthermore, the following information were extracted for all experiments: sample size (total/ AN group/ HC group), coordinate space (MNI vs. TAL), contrast (AN < HC/ AN > HC), age group (adolescents vs. adults), AN status (acute/ short-rec/ longer-rec), mean age, mean BMI, mean illness duration (plus respective standard deviation), measure of brain structure (GM volume/ CT), and analysis software used.

Data extraction for both the global brain volume meta-analysis and the ALE meta-analyses was carried out by LK supervised by JS. Pooled age and BMI per age group were calculated to allow for a better characterization of participants (patients and HCs) included in both the global brain volume meta-analysis and the ALE meta-analyses. Furthermore, risk of bias for all studies that were included in the global brain volume meta-analysis as well as the ALE meta-analyses was assessed using a modified version of the Newcastle-Ottawa Scale (NOS; https://www.ohri.ca/programs/clinical_epidemiology/oxford.asp, Wells et al., 2024). We adapted the NOS to enhance its applicability in the evaluation of the methodological quality of cross-sectional case-control studies. We replaced the original NOS “Exposure” domain with an “Outcome” domain (i.e. brain structure). This corresponds better to the analytical focus of cross-sectional neuroimaging studies, where observed outcomes instead of exposures are central, allowing us to assess how well each study measured and incorporated the outcome of interest. The scoring scheme included 8 items divided into 3 categories (selection, comparability and outcome) resulting in a maximum of 9 stars per study that can potentially be achieved. Details on our modified NOS can be found in the Supplementary Materials under '4 Detailed information on risk of bias assessment’. The NOS rating was performed independently by two reviewers (LK and KO). Any discrepancies between the two reviewers were discussed and resolved by reaching a consensus.

### 2.3 Meta-analysis of global brain volume differences

The statistical analysis of global brain volume differences was conducted using Review Manager (RevMan) 5.4 (The Cochrane Collaboration, 2020). For each stage of disease (AN_acute_/AN_short-rec_/AN_longer-rec_), the reported raw values of GM, WM and CSF, the respective standard deviations, and the number of patients with AN and HCs of each study were entered into the software. We extracted and included reported volume data and its standard deviation from all neuroimaging software tools in our meta-analysis. As CSF is calculated differently depending on the analysis software used, only reported values of CSF determined by the most commonly used analysis method VBM were considered for the meta-analysis of CSF. Random-effects models with 95 % confidence intervals (CIs) and standardized mean differences (SMD) for global GM, WM, and CSF volume across all stages of AN were calculated using Hedges’ g as standardized effect size. Effect sizes ≥ 0.2 can be classified as small, ≥ 0.5 as medium, and ≥ 0.8 as large (Cohen, 1988). For AN_acute_ and AN_short-rec_, subgroup analyses were performed for adults and adolescents to gain further insight into age-related differences. In AN_longer-rec_, only two studies reported results for adolescent patients with AN, making a differentiation between age groups after longer-term recovery difficult. For illustrative purposes and from an exploratory point of view, we nevertheless calculated the analyses separately for adults and adolescents to give a first impression. For all subgroup analyses, a Chi^2^ test of effect sizes (as implemented in RevMan 5.4) was performed to evaluate whether effects differed significantly between adolescent and adult patients.

To quantify heterogeneity between studies, the I^2^ statistic as a measure of the extent of heterogeneity, the Tau^2^ statistic as a measure of between-study variance and Chi^2^ (χ^2^) tests assessing statistically significant heterogeneity were used. For all analyses, two-tailed tests were performed at a 5 % significance level. To make alterations more tangible, a weighted average volume change in percent across all studies was calculated by multiplying the number of patients in each study (or subgroup respectively in case of subgroup analyses) by the respective volume change, summing the results and dividing this value by the total number of patients in that study or subgroup. As not every article included values for all three compartments (GM, WM, and CSF), analyses were calculated with different sample sizes.

### 2.4 Anatomical likelihood estimation (ALE) meta-analysis

In our study, ALE meta-analysis was applied to aggregate findings from single neuroimaging studies reporting regional structural differences in GM volume and CT (collectively referred to as GM) between patients with AN and HCs. We included results from VBM and surface-based studies conducted with all commonly used neuroimaging software packages. The basic principle of ALE meta-analyses is that coordinates for activations or anatomical findings reported in studies are not interpreted as absolute values, since a certain degree of spatial uncertainty cannot be excluded in neuroimaging studies. Instead, the reported coordinates are regarded as “foci” of spatial probability distributions, accounting for spatial uncertainty, and local convergence across studies is assessed (Eickhoff et al., 2009). ALE can overcome common problems of single imaging studies such as being underpowered due to small sample sizes involving the danger of false positive and negative findings, low reproducibility, and overgeneralization of results (Eickhoff et al., 2016). A detailed explanation of the ALE methodology and the specific algorithm applied in this study can be found in the Supplementary Materials under '1 Supplementary Methods'. According to existing guidelines for best practices (Eickhoff et al., 2016, Eklund et al., 2016), a primary voxel-wise threshold of *p* < 0.001 was used to form clusters. By means of 10,000 Monte Carlo simulations, significance of clusters at this voxel height was determined. Threshold-free cluster enhancement (TFCE) correction was applied to correct for multiple comparisons as it is more sensitive towards the detection of smaller, spatially restricted clusters (Han et al., 2019) and eligible in the context of ALE meta-analyses (Frahm et al., 2022). Clusters were considered significant if they reached a TFCE-corrected significance of *p* < 0.05. The JuBrain Anatomy toolbox, version 3.0 (https://github.com/inm7/jubrain-anatomy-toolbox; Eickhoff et al., 2006, Eickhoff et al., 2007, Eickhoff et al., 2005) was used to estimate MNI coordinates (x, y, z), the cluster size, and coverage of anatomical regions. This procedure was repeated for each of the ALE meta-analyses performed.

To increase statistical power and to be able to draw the most meaningful conclusions, we decided to perform only directed contrasts (AN < HC or AN > HC) to answer not only the question of regionality of brain changes, but to make statements about the direction of these alterations (i.e. increase or decrease in the specific brain areas). However, our literature search (see below) revealed only six experiments from five papers reporting increases in AN (AN > HC). Since a simulation by Eickhoff and colleagues (Eickhoff et al., 2016) demonstrated that at least 20 experiments should be included into ALE meta-analyses to achieve reliable results and sufficient power, we only performed ALE analyses for the contrast AN < HC, i.e. for a brain volume loss in AN. We first performed an ALE meta-analysis “GM_loss_acute” which included foci from all experiments reporting a reduction in GM volume or CT in patients with acute AN. By means of subsequent analyses with a different focus, we aimed to gain deeper insight into the underlying pathophysiology of AN. Therefore, we complemented the first analysis with two additional ALE meta-analyses: Firstly, a broader analysis that included foci from experiments with patients with both acute AN and recovered individuals (“GM_loss_acute_and_recovered”) was conducted. Second, a more specific ALE analysis that comprised only the foci from adult patients with acute AN (“GM_loss_acute_adults”) was performed. The number of studies reporting coordinates for recovered patients or adolescents with AN was far below the above-mentioned minimum number of studies for ALE meta-analyses. Hence, we did not perform sub-analyses on adolescent or recovered patients.

### 2.5 Functional and biological contextualization of ALE results

#### 2.5.1 Functional decoding using the Neurosynth data base

Manually searching for studies with corresponding brain regions to interpret regional neuroimaging findings and to gain insight into the functional relevance of these brain structural alterations in AN can be somewhat arbitrary. For a deeper and more objective interpretation of our ALE results, we thus followed a data driven approach using the Neurosynth data base (Yarkoni et al., 2011), which is an extensive collection of more than 14,000 neuroimaging studies, annotated with both imaging coordinates and the frequency of > 1,300 “terms” used in each study, based on an automated data-mining approach. For our functional decoding analyses, we curated a list of 28 both AN-related and general behavioral and cognitive terms based on the Research Domain Criteria framework (https://www.nimh.nih.gov/research/research-funded-by-nimh/rdoc; see Fig. 4). We then employed two different functional decoding approaches, aiming at identifying cognitive and behavioral terms associated to (i) regional clusters of structural brain alterations in AN and (ii) the cortex-wide distribution of AN-related brain alterations.

For the regional analysis, we decoded the clusters from the ALE analysis “GM_loss_acute” using the “reverse inference” and “forward likelihood” methods (see Müller et al., 2013 for an application example of this methodology) as implemented via the BrainMapDecoder in NiMARE (Salo et al., 2023, Salo et al., 2020). Specifically, for each term and cluster, all Neurosynth studies, which reported at least one coordinate within the cluster, were collected. The “forward likelihood” was then determined through a binomial test, assessing whether the probability of term-related activation in the cluster, *P(Activation|Term)*, was higher than the baseline probability to observe activation in the cluster *P(Activation)*. The “reverse probability” was obtained from a chi-squared test, examining the probability of a particular term given activation in the cluster, *P(Term|Activation)*, derived by Bayes’ rule (Lotter et al., 2023, Müller et al., 2013). We applied FDR-correction to the resulting *p* values to correct for multiple comparisons.

For the cortex-wide analyses, we calculated spatial Spearman correlations between (i) the “GM_loss_acute” ALE map and (ii) meta-analytic maps computed for each of the 28 terms using the multilevel kernel density analysis chi-square algorithm in NiMARE (Wager et al., 2007) after parcellating all maps into 100 cortical parcels (Schaefer et al., 2018). Statistical significance was estimated by comparing the observed correlation coefficients to null distributions of correlations to 10,000 spatial autocorrelation-preserved topic null maps (Burt et al., 2020, Markello et al., 2022) and applying FDR-correction. The process was implemented using the JuSpyce toolbox (https://github.com/LeonDLotter/JuSpyce; Lotter and Dukart, 2022), which was described in detail and successfully applied before (Lotter et al., 2023, Lotter et al., 2024a, Lotter et al., 2024b). We decided to perform these analyses on only the cortical part of the “GM_loss_acute” ALE map – rather than the whole-brain level – as we aimed to (i) use the map based on the most original experiments (i.e., VBM and CT experiments combined) and (ii) avoid the risk of a biased meta-analytic whole-brain distribution due to simultaneous inclusion of original experiments reporting whole-brain foci (i.e., VBM) and cortex-only foci (i.e., CT). In correspondence with the cluster-level decoding analyses, we worked with the ALE map of the AN < HC contrast, to be interpreted as the continuous probability of any voxel to belong to a cluster of reduced GM in AN. In consequence, a positive whole-cortex correlation revealed by our spatial colocalization analyses would mean that brain regions strongly associated with a term showed a stronger GM reduction. Conversely, negative correlations reflect that brain areas associated with a topic are less likely to be affected by GM reductions.

#### 2.5.2 Spatial associations with neurotransmitter systems

In a last step, we aimed to further investigate the neurobiological underpinnings of altered brain structure in AN by calculating spatial correlation analyses adjusted for autocorrelation (Burt et al., 2020) between affected brain regions and a set of external in vivo neurotransmitter atlases. Again, the results of our ALE analysis “GM_loss_acute” served as input for these whole-brain analyses, following the workflow described above. Instead of the meta-analytic Neurosynth term maps, 21 nuclear imaging neurotransmitter atlases (Supplementary Table 1) were drawn from JuSpace or neuromaps toolbox (Dukart et al., 2021, Markello et al., 2022) and correlated with the ALE map “GM_loss_acute”. Prior to spatial correlation analyses, the atlases were parcellated and atlas-wise Z-standardized. Here again, for the interpretation of results it is important to keep in mind that in our analyses higher ALE scores reflect a larger GM reduction in patients with AN. Negative correlations can thus be interpreted as indicating that in areas where the density of a neurotransmitter system is high, less GM reduction is found and vice versa.

## 3 Results

### 3.1 Included studies and sample characteristics

In total, 53 papers (19 of which concerned adolescents) reported total whole-brain volume scores of GM, WM, and CSF and were selected for the global brain volume meta-analysis. 40 papers included a total of 1130 patients with AN_acute_ (N = 1150 HCs), 11 papers reported a sample of 229 patients with AN_short-rec_ (N = 233 HCs) and 13 papers included 263 longer-term recovered patients with AN (N = 335 HCs). Supplementary Table 2 gives an overview of all studies included in the global brain volume meta-analysis and the reported brain volume changes.

For the ALE analysis, the literature search yielded 35 eligible papers with 47 experiments reporting MNI or TAL coordinates for 412 foci. The vast majority of experiments reported coordinates on regional brain changes in GM volume (n = 34). As stated above, we decided to include only experiments reporting coordinates for reduced GM volume or CT in AN (i.e., contrast AN < HC, n = 41 experiments). Most of the experiments reporting reduced GM volume or CT focused on adult patients with AN (n = 31) and the acute stage of disease (n = 33). For the first ALE analysis “GM_loss_acute” with n = 33 experiments, 817 patients with acute AN and 857 HCs were included. The subsequent ALE meta-analysis “GM_loss_acute_and_recovered” was conducted with n = 41 experiments including 942 patients with AN and 1021 HCs. The more specific sub-analysis “GM_loss_acute_adults” included 783 adults with AN_acute_ and 839 HCs and foci from n = 25 experiments. Details of all studies included in the ALE meta-analyses and the respective foci extracted from the papers are shown in Supplementary Table 3A and B.

Supplementary Table 4 displays the pooled BMI and age for studies included in the various meta-analyses separated for age group and stage of disease. Adult patients with AN_acute_ included in the global brain volume meta-analysis had a pooled age of 24.16 years (HCs: 24.75 years) and a pooled BMI of 15.71 (HCs: 21.92), whereas adolescents with AN_acute_ had a pooled age of 15.58 years (HCs: 15.93 years) and a pooled BMI of 15.13 (HCs: 20.97). Acutely ill adolescent patients included in the ALE meta-analysis (“GM_loss_acute”) had a similar pooled age (15.21 years; HCs: 15.55 years) and BMI (15.09; HCs: 21.06). Pooled age of adults with acute AN included in the ALE meta-analysis in contrast was slightly higher (26.45 years; HCs: 26.77 years) than in acutely ill adult patients of the global brain-volume meta-analysis, but the pooled BMI (15.57; HCs: 21.81) was comparable in adults with AN_acute_ of both meta-analyses. Please refer to Supplementary Table 4 for details on pooled BMI and age of participants from the global brain volume meta-analyses at different stages of recovery.

The NOS rating showed variable total scores ranging from 4 (only one study) to 9 out of 9 stars, with overall acceptable methodological quality of included studies (mean of 6.9 stars for studies included in the global brain volume meta-analysis; mean of 7.6 stars for studies included in the ALE meta-analyses). The NOS total scores for all studies included in the global brain volume meta-analysis and ALE meta-analyses are presented in Supplementary Table 2 and 3B, respectively. Additionally, the detailed NOS rating for each included study is listed in the Supplementary Materials under '4 Detailed information on risk of bias assessment’.

### 3.2 Global brain volume meta-analysis

**Fig. 2:**
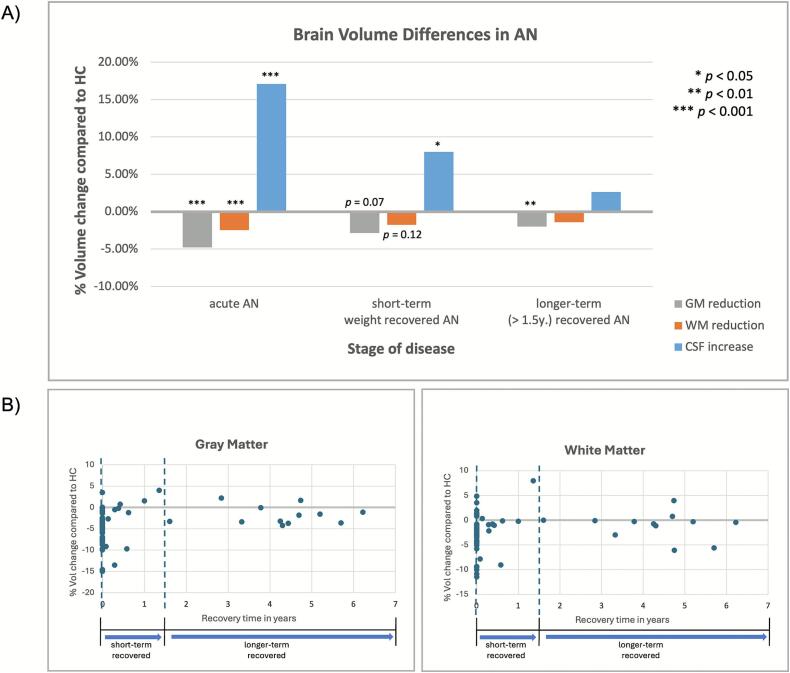
A) Weighted average changes in brain volume (in percent) in GM, WM, and CSF in acute, short-term weight recovered and longer-term recovered patients with AN compared to HCs. B) Time course of brain volume changes at different stages of recovery for GM (left) and WM (right). The x-axis represents the recovery time in years and the y-axis displays the average brain volume difference (in percent) between patients and HCs. *AN,* Anorexia nervosa; *CSF,* Cerebrospinal fluid; *GM,* Gray matter; *HC,* Healthy controls; *WM,* White matter.

The weighted average volume changes in GM, WM, and CSF at the different stadiums of disease (AN_acute_, AN_short-rec_ and AN_longer-rec_) are displayed in Fig. 2A and Supplementary Table 5. The indicated significance levels are based on the RevMan analyses. Highly significant brain volume changes for all three compartments (GM, WM, and CSF) were found in acutely ill patients with AN (Supplementary Fig. 1A–C). Global GM volume was reduced by 4.79 % (SMD and 95 % CI: −0.59 [-0.72; −0.47], *p* < 0.001), with adolescents (−6.31 %) showing a numerically greater percentual volume change than adults (−4.13 %), although the effect sizes did not differ significantly between the two age groups (SMD adolescents: −0.72, SMD adults: −0.55; test for subgroup differences: *p* = 0.27). An average WM volume loss of 2.48 % (SMD and 95 % CI: −0.27 [-0.37; −0.17], *p* < 0.001) was found in acutely ill patients (adolescents: −3.48 %, adults: −1.99 %,) and CSF was increased by 17.11 % (SMD and 95 % CI: 0.80 [0.55; 1.06], *p* < 0.001; adolescents: +16.75 %, adults: +17.22 %). For both compartments, no significant difference in effect sizes between adolescents and adults was observed (test for subgroup differences: WM: *p* = 0.16, CSF: *p* = 0.76). There was moderate to considerable heterogeneity between studies reporting GM (I^2^ = 44 %) and CSF (I^2^ = 71 %) volume scores, while studies reporting WM volumes showed low heterogeneity (I^2^ = 19 %).

After short-term recovery (Supplementary Fig. 2A–C), GM volume was still numerically reduced by on average 2.86 %, even though not statistically different from HCs (SMD and 95 % CI: −0.27 [−0.57; 0.02], *p* = 0.07). Looking at subgroups, the GM reduction in adolescents (−5.43 %) showed a non-significant trend towards being reduced (*p* = .10), while in adults the GM loss of 1.43 % differed no longer from HCs (*p* = .41). A numerical reduction in WM volume by 1.76 % (adolescents: −2.32 %, adults: −1.44 %) was observed, which did not reach significance (SMD and 95 % CI: −0.17 [-0.39; 0.05], *p* =.12). Additionally, a still persistent significant increase in CSF by 7.99 % (adolescents: 6.78 %, adults: 9.11 %) was observed (SMD and 95 % CI: 0.46 [0.05; 0.87], *p* <.05). No significant difference in effect sizes between adolescents and adults was detected (test for subgroup differences: GM: *p* = 0.23, WM: *p* = 0.66, CSF: *p* = 0.61). Moderate to high heterogeneity was found for studies included in the analysis of GM (I^2^ = 49 %) and CSF (I^2^ = 68 %), while studies on WM showed low heterogeneity (I^2^ = 12 %).

In longer-term recovered patients with AN (Supplementary Fig. 3A–C), in the combined analysis including both adolescents and adults global GM volume was significantly reduced by 1.98 % (SMD and 95 % CI: −0.31 [-0.50; −0.11], *p* < 0.01). The more exploratory subgroup analysis differentiating between adults and adolescents with AN_longer-rec_, showed that in the adult subgroup there was a significant reduction in GM volume by −1.48 % (SMD and 95 % CI: −0.27 [-0.51; −0.03], *p* < 0.05) compared to HCs. In the adolescent subgroup as well, a significant GM volume reduction by 3.63 % was found (SMD and 95 % CI: −0.46 [-0.84; −0.08], *p* < 0.05). However, the limited data available for adolescents (two studies including in total only 55 patients and 54 HCs) restricts the interpretability of this finding and it should be viewed as a descriptive result reported for completeness rather than as a robust subgroup effect. WM volume scores were numerically reduced by 1.40 % and CSF volumes increased by 2.63 % in AN_longer-rec_ compared to HCs. However, the volume scores of WM (SMD and 95 % CI: −0.11 [-0.29; 0.06], *p* = 0.20) and CSF (SMD and 95 % CI: 0.16 [-0.09; 0.42], *p* = 0.22) between patients and HCs did not differ significantly. Tests for subgroup differences revealed no significant differences in effects sizes of adolescents and adult patients (test for subgroup differences: GM: *p* = 0.40, WM: *p* = 0.67, CSF: *p* = 0.22). Studies on CSF in AN_longer-rec_ again showed moderate heterogeneity (I^2^ = 37 %), heterogeneity was low for GM (I^2^ = 19 %) and no evidence for heterogeneity was found for WM (I^2^ = 0 %).

The time course of brain volume changes in GM and WM at different stages of recovery is shown in Fig. 2B. In general, differences between patients and HCs decrease as recovery progresses.

### 3.3 ALE meta-analyses

**Fig. 3:**
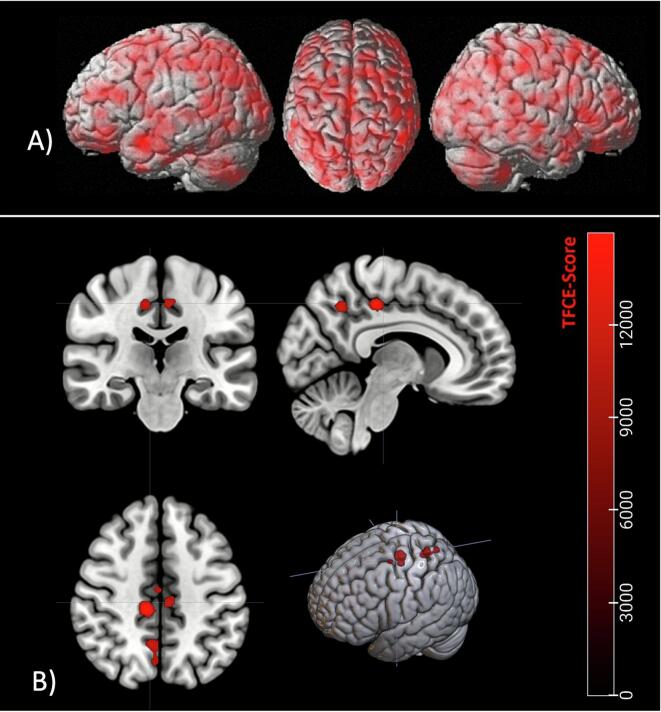
A) Example of an unthresholded Z-map derived from ALE *p* values for the analysis “GM_loss_acute” revealing a widespread distribution of foci from included single studies. B) Results from the directed ALE meta-analysis of structural differences in gray matter volume and cortical thickness in AN displaying TFCE-corrected significant clusters which show reductions in patients with acute AN compared to healthy controls (ALE analysis “GM_loss_acute”). *ALE*, Anatomical likelihood estimation; *AN*, Anorexia nervosa; *GM,* Gray matter; *TFCE*, Threshold-free cluster enhancement.

Overall, foci reported in individual studies were widespread and generally distributed across the brain. As an example, Fig. 3A shows an unthresholded Z-map derived from ALE *p* values for the analysis “GM_loss_acute”. Nevertheless, after applying TFCE-correction to account for multiple testing, significant clusters of local convergence across studies were found for each of the analyses performed. First, the ALE meta-analysis “GM_loss_acute” revealed four significant clusters of reductions in GM volume and CT in patients with acute AN compared to HCs. The identified clusters comprised the bilateral precunei, the right cingulate gyrus (anterior and posterior division), the left cingulate gyrus (posterior division) and the bilateral precentral gyri (Fig. 3B, Table 1). The broader ALE meta-analysis “GM_loss_acute_and_recovered”, which contained foci from studies reporting differences in patients with both acute AN and recovered individuals compared to HCs, detected reduced GM volume in eight significant clusters, including the same brain regions as in the previous analysis. Additionally, the juxtapositional lobule cortex, the left lateral occipital cortex (superior division), and the left cuneal cortex were identified (Supplementary Fig. 4, Supplementary Table 6). The more specific sub-analysis “GM_loss_acute_adults”, which included only foci from adult patients with acute AN, reproduced a very similar pattern and found additional differences in the right lateral occipital cortex (superior division) and the left angular gyrus (Supplementary Fig. 5, Supplementary Table 7).

**Fig. 4:**
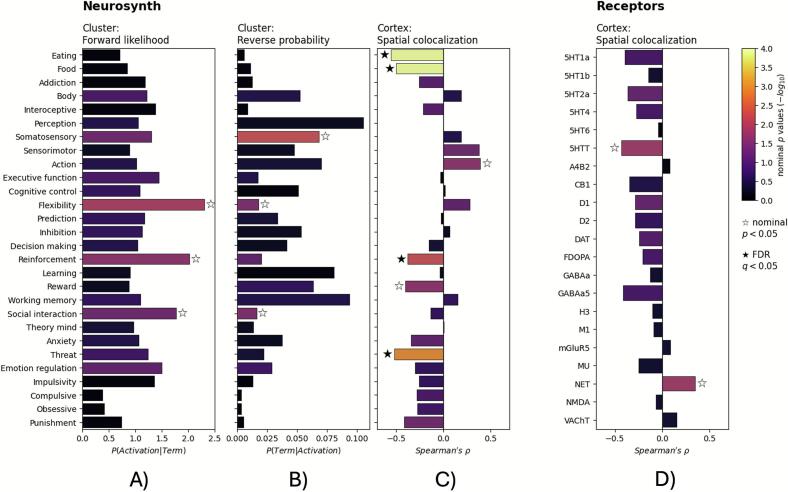
Results of the functional and biological contextualization of ALE results. A) and B): Functional decoding for clusters of GM volume and CT reduction revealed by the ALE analysis “GM_loss_acute” based on forward (A) and reverse (B) inference. C) Spatial correlation between the ALE map of the analysis “GM_loss_acute” and meta-analytic maps for AN-related Neurosynth terms. D) Spatial correlation between the ALE map of the analysis “GM_loss_acute” and neurotransmitter atlases. Bar color: Uncorrected −log10(p) values. AN, Anorexia nervosa; ALE, Anatomical likelihood estimation; CT, Cortical thickness; GM, Gray matter.

**Table 1:** Structural differences in gray matter volume and cortical thickness between patients with acute AN and HCs revealed by the directed ALE analysis “GM_loss_acute” reporting significant clusters (TFCE-corrected) of brain volume loss in AN including MNI coordinates, cluster size and experiments contributing to the cluster.

Cluster	Region	MNI			Cluster size (voxels)	N (experiments contributing to cluster)	References (experiments contributing to cluster)
		x	y	z			
1	Precuneous cortex Cingulate gyrus, posterior division	8 −4 4	−54 −54 −68	42 54 48	138	8	(Bär et al., 2015, Fonville et al., 2014, Gaudio et al., 2011, Martin Monzon et al., 2017, Mishima et al., 2021, Mishima et al., 2021, Nickel et al., 2018, Yu et al., 2024)
2	Cingulate gyrus, posterior division Precentral gyrus	10	−28	44	107	7	(Amianto et al., 2013, Bär et al., 2015, Boghi et al., 2011, Friederich et al., 2012; Gaudio et al., 2017, Nickel et al., 2018, Seitz et al., 2015)
3	Precentral gyrus Cingulate gyrus, posterior division	−6	−24	46	36	7	(Bär et al., 2015, Gaudio et al., 2017, Kohmura et al., 2017, Martin Monzon et al., 2017, Seitz et al., 2015, van Opstal et al., 2015, Yu et al., 2024)
4	Cingulate gyrus, anterior division Cingulate gyrus, posterior division	2	−14	46	11	6	(Bär et al., 2015, Friederich et al., 2012; Gaudio et al., 2017, Kohmura et al., 2017, Martin Monzon et al., 2017, Yu et al., 2024)

Abbreviations: *AN*, Anorexia nervosa; *GM*, Gray matter; *HCs*, Healthy controls; *MNI*, Montreal Neurological Institute; *TFCE*, Threshold-free cluster enhancement.

### 3.4 Contextualization of ALE results

#### 3.4.1 Functional decoding using Neurosynth data base

We found associations between the clusters of reduced GM volume and CT revealed by the ALE meta-analysis “GM_loss_acute” and the Neurosynth terms both for forward and reverse inference at the nominal significance level of *p* < 0.05. The forward likelihood analysis showed an association between brain areas being affected by volume reduction and the terms “flexibility”, “reinforcement”, and “social interaction” (Fig. 4A, Supplementary Table 8). In the reverse probability analysis, an association of affected brain regions and the terms “somatosensory”, “social interaction”, and “flexibility” was observed (Fig. 4B, Supplementary Table 9). However, none of these associations survived FDR-correction.

The spatial colocalization analysis yielded both positive and negative correlations between the ALE map for the analysis “GM_loss_acute” and the meta-analytic maps of AN-related Neurosynth terms (Fig. 4C, Supplementary Table 10). We observed the strongest negative correlations with the terms “eating”, “food”, “threat”, and “reinforcement” (FDR-corrected), suggesting that brain regions associated with these functions are less likely affected by brain volume loss. A further negative association, significant at the uncorrected level, was found with “reward” (nominal *p* < 0.05). Conversely, a nominally significant positive association was detected for the term “action”, indicating that brain areas associated with this term seem to be especially affected by volume reductions (nominal *p* < 0.05).

#### 3.4.2 Spatial associations with neurotransmitter systems

The spatial colocalization analyses with neurotransmitter atlases indicated a nominally significant negative association between the ALE map of the analysis “GM_loss_acute” and the distribution of serotonin transporters (5-HTT) (nominal *p* < 0.05; Fig. 4D, Supplementary Table 11). Thus, brain areas in which the availability of 5-HTT is high tend to be less affected by the volume reduction. Furthermore, a positive association, significant at the uncorrected level, with the noradrenaline transporter (NET) was found (nominal *p* < 0.05; Fig. 4D, Supplementary Table 11), suggesting that brain regions with a higher density of NET are particularly affected by brain volume reductions. After FDR-correction, both correlations were no longer significant. No other significant correlations were observed.

## 4 Discussion

Our meta-analysis aimed to quantify and localize structural brain differences in patients with AN and to evaluate their possible clinical and functional impact by updating and extending previous work in this field. The results of our global brain volume meta-analysis demonstrate profound volume changes in GM, WM, and CSF in the acute stage of disease that decline quickly with recovery. Nevertheless, even after 1.5 years of recovery a significantly lower GM volume in longer-term recovered patients with AN compared to HCs was detected, statistically confirming trends already observed in previous meta-analyses (Seitz et al., 2014, Seitz et al., 2016, Seitz et al., 2018). This makes further research into the underlying pathophysiology and functional consequences even more pressing and longitudinal studies are needed to determine which patients might be threatened by this long-lasting GM loss. However, there is no consensus on the definition of recovery in AN (Bardone-Cone et al., 2018, Solmi et al., 2024). Therefore, the use of this term must be critically examined across different studies, which needs to be considered when interpreting this result.

The ALE meta-analyses revealed mostly globally distributed changes with significant clusters affected more severely including the cingulate gyrus, precentral gyrus, and precuneus. The functional decoding approach indicated that brain regions associated with eating, food, threat, and reinforcement processing were less likely to be affected by volume reductions which could be interpreted as regions of constant use in patients with AN to remain relatively preserved. Preliminary results, significant only at uncorrected alpha levels not surviving FDR-correction, indicated that especially affected regions might be associated with social interaction, flexibility, reinforcement, and somatosensory functions. In contrast, further exploratory, only nominally significant neurotransmitter results suggested brain areas with a higher density of serotonin transporters to be less affected, while regions with a higher availability of noradrenaline transporters seem to be particularly impacted by brain volume loss, potentially helping to explain AN symptomatology.

### 4.1 Global volume changes in GM, WM and CSF

As hypothesized, our global brain volume meta-analysis revealed significantly decreased GM (−4.79 %) and WM (−2.48 %) volumes as well as increases in CSF (+17.11 %) in patients with acute AN. These results are consistent with previous meta-analyses from our group (Seitz et al., 2014, Seitz et al., 2016, Seitz et al., 2018), a meta-analysis by Titova and colleagues (Titova et al., 2013) and systematic reviews by Vidal and colleagues (Vidal et al., 2021) and Kappou and colleagues (Kappou et al., 2021), who all described clear alterations in global brain volumes in acute AN. As in previous meta-analyses investigating the course of disease in AN (Bahnsen et al., 2022, Seitz et al., 2014, Seitz et al., 2016, Seitz et al., 2018, Walton et al., 2022), our data showed a relatively rapid restitution of these differences during recovery. In short-term recovered patients, CSF volume continued to differ significantly from HCs, while only non-significant trends for residual changes in GM and WM in the patient group were found. Our analysis of global brain volumes in patients recovered for more than 1.5 years revealed a remaining significant reduction of GM volume of −1.98 % indicating persistent structural brain alterations even in longer-term recovered patients. Previous meta-analyses (Seitz et al., 2014, Seitz et al., 2016, Seitz et al., 2018) found effects in the same direction, even though not statistically significant. Possibly, the inclusion of four additional recent studies and N = 82 new participants recovered from AN in our current meta-analysis and a subsequent enhancement in statistical power contributed to detecting this effect.

The pathophysiology underlying these brain volume reductions remains largely unclear, although several factors that may contribute to the brain volume loss are discussed in the literature: First, significant positive associations between body mass index and brain volume loss as well as its regeneration during weight recovery were demonstrated in several studies (e.g., Amianto et al., 2013, Bahnsen et al., 2022, Bang et al., 2021, Bernardoni et al., 2016, Geisler et al., 2022, Kaufmann et al., 2020, Mühlau et al., 2007, Seitz et al., 2015, Walton et al., 2022), highlighting the pivotal role of body weight as one of the possibly most influential factors in the context of brain volume reduction. Moreover, astrocyte loss is discussed to be relevant in the context of brain volume reductions. Astrocytes are important for e.g., nourishing neurons, forming of the blood–brain-barrier, neurotransmitter reuptake and synapse formation as well as for cognitive abilities like learning and memory (Barres, 2008, Bélanger et al., 2011, Fields et al., 2015). In consequence, a loss of astrocytes could help explain the well-documented neuropsychological deficits in the acute stage of AN (Miles et al., 2020, Stedal et al., 2021). This assumption is supported by animal studies using the well-established activity-based anorexia (ABA) model which mimics AN through a combination of food reduction and running wheel access showing reduced learning and memory in chronically starved ABA animals (Paulukat et al., 2016). A drastic reduction of astrocytes in the cerebral cortex and corpus callosum, but no change in the mean number of neurons, and a reduced glial fibrillary acid protein (GFAP) mRNA expression and cell proliferation rate was found in ABA animals compared to controls (Frintrop et al., 2019). Longitudinally, the starvation-induced alterations in ABA animals also largely disappeared after refeeding hinting at a reversibility of astrocyte depletion through fast weight recovery (Frintrop et al., 2019). These findings suggest that astrocyte rather than neuronal cell reductions could be responsible for the brain volume loss in acute AN, as astrocytes appear to regrow quickly, potentially explaining the rapid restoration of brain volume losses during weight recovery which was demonstrated by the current as well as previous meta-analyses of our group (Seitz et al., 2014, Seitz et al., 2016, Seitz et al., 2018) and other authors (Bahnsen et al., 2022, Walton et al., 2022). Also, serum markers of cell damage have been studied in this context. Patients’ serum GFAP (astrocytes), tau protein, and neurofilament light (NF-L) chain levels (both neurons) were found to be increased during the acute stage of disease indicating both astroglial and neuronal damage (Hellerhoff et al., 2021). While GFAP and NF-L normalized after partial weight recovery in this study by Hellerhoff and colleagues, tau protein levels remained to be significantly elevated indicating only partial restitution of neurons after weight restoration (Hellerhoff et al., 2021). Wentz and colleagues demonstrated increased serum NF-L concentrations even 30 years after disease onset, suggesting persistent neuronal injury (Wentz et al., 2021). This points to also partial neuronal damage, even if the cells themselves appear to be able to survive. As astrocytes support neurons with regard to energy, neurotransmitter reuptake and synapse formation (Barres, 2008, Bélanger et al., 2011, Fields et al., 2015), an intimate interplay between both cell types can be expected, potentially also explaining impaired functioning. Another recent study by Hellerhoff and colleagues also detected a negative association between serum NF-L levels and cortical thickness in several brain regions suggesting axonal damage processes as a possible cause of cortical thinning in AN (Hellerhoff et al., 2023). Besides, Zhou and colleagues showed that genes associated with normal patterns of cortical thinning during childhood and adolescence are primarily expressed in astrocytes, microglia, as well as excitatory and inhibitory neurons (Zhou et al., 2023).

Furthermore, hormones appear to play a significant role among potential factors influencing or mediating brain volume loss in AN. Decreased thyroid hormone and/or elevated cortisol levels have been linked to brain volume loss in patients with AN (Castro-Fornieles et al., 2009, Chui et al., 2008, Nogal et al., 2008). Likewise gonadal hormones like estradiol are clearly altered in AN (see Haines, 2023 for an overview) and could be related to brain volume loss in patients. Nogal and colleagues found enhanced hypogonadotropic hypogonadism in patients with AN to be associated with increased width of cortical sulci as an indirect measure of trophic changes of the brain (Nogal et al., 2008). Additionally, in a study by Mainz and colleagues increases in gray matter volumes after weight recovery were correlated with increases in follicle-stimulating hormone levels (Mainz et al., 2012). Moreover, a lack of brain-derived neurotrophic factor and leptin was found in AN (Brandys et al., 2011, Hebebrand et al., 2007, Karageorgiou et al., 2020, Keeler et al., 2022) and could potentially be associated with brain structural alterations. For instance, a negative association between hypoleptinemia and a greater reduction of rostral-medial amygdala nuclei volumes was revealed by Wronski and colleagues (Wronski et al., 2023). Occasionally, disturbances in micro- and macronutrient profiles (e.g., polyunsaturated fatty acids) and consequently, lipid depletion or altered lipid metabolism in the brain are discussed as potentially relevant factors in the context of structural brain alterations in AN (Bernardoni et al., 2018, Shih et al., 2016), as there is evidence for an association between omega-3 fatty acids and GM volumes, WM resilience, and microstructural integrity in healthy individuals (McNamara et al., 2018). Lastly, several previous studies using different blood and urine markers have found no evidence supporting dehydration as an explanatory factor for brain volume changes in AN, rendering this potential explanation unlikely (e.g., Bernardoni et al., 2016, King et al., 2015, Vogel et al., 2016).

Looking at subgroup differences, in line with previous meta-analyses by our group (Seitz et al., 2014, Seitz et al., 2016, Seitz et al., 2018), our study continued to identify numerically greater percentual volume changes in adolescent patients with acute AN than in adults, although effect sizes did not differ significantly between both age groups. One possible explanation for this finding might be the increased brain plasticity during adolescence that could implicate a greater susceptibility for starvation effects. Subgroup analyses also hint at a different speed of recovery of brain volumes in adolescents and adults: The drastic GM volume alterations of the acute stage of disease appear to ameliorate more quickly in adults, being improved by more than half after short-term recovery. In contrast, in adolescents the recovery of GM volume seems to take longer, showing merely an improvement after short-term recovery with a persisting numerical GM volume reduction of −5.43 %. However, this observation did not reach significance and remained at trend-level, possibly due to limited statistical power of the subgroup size. Again, one reason for this could be that the brain might be more vulnerable towards brain damage during adolescence, as this is a critical period of ongoing developmental processes (Mills et al., 2021) which might be more severely affected by AN in adolescents. This is reflected in recent findings by Moreau and colleagues (Moreau et al., 2025) who described more pronounced structural brain changes in early-onset AN compared to typical-onset cases, hinting at heightened vulnerability during earlier developmental stages. Notably, it should also be considered that neurodevelopment during adolescence is characterized by normative, dynamic processes including cortical thinning due to mechanisms like synaptic pruning (see e.g., He et al., 2025, Parker et al., 2020). These processes could potentially interact with disease-related structural changes in adolescents with AN informing the interpretation of altered GM volume within this age group. We believe that the study designs including healthy adolescents without AN of the same age should control for most effects of normal developmental trajectories in our findings. However, the fact that adolescent patients had numerically greater percentual brain volume reduction should be interpreted incorporating the generally larger brain volumes and ongoing developmental brain restructuring in this age group.

After longer-term recovery, the joint GM volume reduction was clearly decreased (−1.98 %). Whereas adolescents’ case numbers were too low to interpret them separately with only two studies and N = 55 adolescent patients being recovered for more than 1.5 years, from our results in adults we can infer that little further improvement of GM volume reduction was detected from short-term rehabilitation to longer-term recovery, potentially hinting at enduring brain alterations. This could implicate that adult patients with AN show different dynamics of recovery from adolescents. One possible explanation for this could be the oftentimes longer duration of disease in adult patients compared to adolescents. Being ill and severely underweight for a long period of time could thus lead to persisting brain changes which were detected even 1.5 years after recovery in this meta-analysis. This could correspond to brain volume reductions in underweight adult patients with AN being correlated with duration of illness (Boghi et al., 2011, Fonville et al., 2014). In this context, clinical factors such as age of onset, illness duration, and details on recovery history (e.g., number of remittance phases) would be relevant to consider directly within a mega-analysis. However, this was not feasible in the present work due to our methodology. Future research should systematically assess and report these variables to allow for a detailed analysis of clinical characteristics.

For clinical practice, these results further emphasize that gaining weight as quickly as possible could be crucial to prevent long-term damage to the brain which could possibly be involved in negative outcomes, relapses, chronicity, and residual AN psychopathology. Especially in the context of severe and enduring AN, a term used to describe a group of patients with an illness duration of more than seven years who have experienced several failed treatment attempts (Broomfield et al., 2021, Ramos et al., 2022, Touyz et al., 2013), this seems particularly important. A recent study by Kaufmann and colleagues (Kaufmann et al., 2023), for example, demonstrated reduced intrinsic connectivity and changes in network topology in patients with severe AN (mean illness duration of 5.88 years). These alterations persisted even after treatment, hinting at remaining changes in information processing among patients who have experienced severe illness, despite weight restoration (Kaufmann et al., 2023).

Another interesting finding of our global brain volume meta-analysis is that WM volumes appear to improve faster and more completely than GM reductions being only significantly altered in the acute stage of disease. WM changes have also been investigated using diffusion tensor imaging studies hinting at an altered microarchitecture of WM tracts in patients with acute AN (see e.g., Barona et al., 2019, Meneguzzo et al., 2019, Zhang et al., 2020). However, studies report heterogeneous results regarding increases or decreases in WM microstructural integrity (Kappou et al., 2021), and while some studies found WM microarchitectural differences to be largely reversible upon weight gain (e.g., Bang et al., 2018, Maier et al., 2022, von Schwanenflug et al., 2019), which is in accordance with the results from our meta-analysis, others found persistent differences after weight recovery in some measures (e.g., Frieling et al., 2012, Griffiths et al., 2021, Shott et al., 2016).

In sum, brain structural alterations could potentially help explain underlying psychopathology, rigidity, memory deficits, and difficulties in psychotherapy of patients with AN. Therefore, it is of great importance that future studies comprise longitudinal data, more participants, and account for age and duration of illness to broaden the understanding of potentially distinct alterations in brain structure, and to clarify which patients are mostly affected and whether the recovery process in adolescent and adult patients truly varies.

### 4.2 Regional changes in GM volume and CT

In our three-level ALE meta-analyses, we observed rather widespread and spatially diffuse patterns of reduced GM volume and CT throughout the entire brain. This fits well with recent large-scale studies (Bahnsen et al., 2022, Walton et al., 2022) reporting widespread reductions of CT and GM volumes. However, through our ALE meta-analytic approach we were also able to detect significant clusters of altered brain structure even more affected by brain volume loss. Although the identified clusters were relatively small, they can help shed light on regions that are particularly affected in patients with AN. These clusters together with the cortex-wide distribution of AN-related brain alterations served as input for the subsequent further contextualization by means of functional decoding and spatial colocalization analyses.

The first ALE analysis “GM_loss_acute” including all foci from papers identified by our comprehensive literature search reporting a reduction in GM volume and CT in patients with acute AN, revealed four significant clusters. These clusters involved the bilateral precunei, the right anterior and bilateral posterior division of the cingulate gyrus as well as the bilateral precentral gyri. These are regions that showed to be significantly reduced in previous large-scale studies, reviews, and meta-analyses, too (Sader et al., 2022, Su et al., 2021, Tose et al., 2024, Vidal et al., 2021, Walton et al., 2022, Zhang et al., 2018). However, in contrast to the study of Walton and colleagues (Walton et al., 2022) in which alterations in CT and subcortical volumes were analyzed separately, the regions identified by our ALE meta-analysis were only found in Walton’s analysis of changes in CT, whereas their analysis of reduced subcortical volumes revealed different regions (accumbens, amygdala, caudate, hippocampus, pallidum, putamen and thalamus) that were not found in our meta-analysis. In contrast to the ALE meta-analysis by Sader and colleagues (Sader et al., 2022), we decided not to perform meta-analyses of GM increases in patients with AN as the available literature did not meet the required minimal number of studies recommended by Eickhoff and colleagues (Eickhoff et al., 2016). In the subsequent, broader ALE analysis “GM_loss_acute_and_recovered” in which foci from recovered patients with AN were added and in the most specific sub-analysis “GM_loss_acute_adults” including only foci from papers reporting reduced GM volume and CT in adults with acute AN, the same significant regions as in the first ALE meta-analysis were discovered. This reflects a high degree of concordance between the analyses, even if either a wider or a more specific contrast is chosen which hints at an overall great stability of our findings in patients with acute AN, making it unlikely that the effects might be driven by foci reported in papers on only recovered or only adolescent patients. However, this does not allow for separate conclusions about clusters of altered brain structure in recovered or adolescent patients. For this, ALE analyses including only foci of these patient groups would be needed, which unfortunately could not be carried out due to the insufficient number of studies. Additionally, in the latter two ALE meta-analyses, further smaller clusters and additional regions were detected comprising among others the superior division of the bilateral lateral occipital cortices, the left cuneal cortex and the left angular gyrus. The reasons underlying the finding that in both the broader ALE analysis “GM_loss_acute_and_recovered” and in the more specific sub-analysis “GM_loss_acute_adults” a larger number of regions with significantly altered GM were found remain unclear. The results of the ALE analysis “GM_loss_acute_adults”, in which less participants were included compared to the other two ALE analyses, might indicate that the brains of adult patients with AN are differently affected by brain volume loss compared to adolescent patients. This could be interpreted in different ways. It could hint at the additional brain areas being indicative of a longer illness duration as adult patients often tend to be suffering from AN for a longer period of time than adolescents. According to this, these brain regions could potentially reflect neurobiological markers for chronicity of the disease. It might also be the case that the group of adult patients or their brain changes might be more homogenous than the group of adolescents or their brains, potentially due to developmental processes happening during adolescence (Mills et al., 2021). Due to this homogeneity in adults, the ALE analysis might detect a higher degree of local convergence of foci across studies which results in more significant clusters for this group. Unfortunately, we could not conduct separate ALE meta-analyses with foci of adolescent patients only as not enough papers reported coordinates of structural brain alterations in this age group. Future studies should aim to fill this gap.

### 4.3 Functional relevance of structural brain alterations

#### 4.3.1 Associations of structural brain changes with AN-related cognitive and functional terms

Our functional decoding approach and the spatial colocalization analyses allowed us to systematically link volumetric changes to cognitive and biological function, an analysis that is novel in research in AN. The spatial colocalization analysis revealed significant negative associations between our ALE meta-analytic map of reduced brain volume (ALE analysis “GM_loss_acute”) and meta-analytic maps for the Neurosynth terms “eating”, “food”, and “threat”, all of which survived FDR-correction. Possibly, the permanent cognitive preoccupation with food and eating of patients with acute AN and the subjective feeling of threat that accompanies it engages the respective brain areas associated with these terms and might, thus, partially prevent brain volume loss in these regions. This is in line with previous findings demonstrating that food stimuli are experienced particularly aversive and threatening in AN (see e.g., Joos et al., 2011b, Levinson and Byrne, 2015, Levinson and Williams, 2020, Uher et al., 2004). Moreover, fMRI studies found aberrant neural responses to food cues in patients with AN compared to HCs (Gizewski et al., 2010, Joos et al., 2011b, Kerr et al., 2017), including an increased activation of medial prefrontal regions during food stimuli processing, potentially due to efforts to control and avoid food (Cowdrey et al., 2011, Kim et al., 2012, Rothemund et al., 2011, Uher et al., 2004), and an elevated amygdala reactivity (Joos et al., 2011b). A study by Wronski and colleagues investigated associations between food- and weight-related mental rumination and amygdala volumes in AN (Wronski et al., 2023). They detected decreased right accessory basal and cortical nuclei volumes to be predictive for an increased frequency of weight-related rumination but observed no relationship between rostral-medial amygdala nuclei volumes and food-related rumination (Wronski et al., 2023). To our knowledge, other structural MRI studies that directly correlate volumes of relevant brain regions with eating- or food-related preoccupation measures in AN are lacking so far, limiting the body of research on this topic to studies from a functional perspective (see e.g., Brooks et al., 2012, Seidel et al., 2018) or studies drawing indirect conclusions about the functional relevance of brain areas showing volume reductions.

Spatial colocalization analyses indicated further negative associations with the terms “reinforcement” and “reward”, suggesting that brain areas associated with these terms are less likely affected by volume reductions. However, only the association with the term “reinforcement” survived FDR-correction. An additional spatial colocalization analysis also hinted at a positive association with the term “action”, suggesting that brain regions associated with this term could be especially affected by brain volume loss. However, this finding did not survive multiple comparison correction and must be regarded as exploratory. Furthermore, our functional decoding approach revealed associations with the terms “flexibility”, “social interaction”, “reinforcement”, and “somatosensory”, which again did not survive FDR-correction and should therefore be considered as preliminary and hypothesis-generating. Interestingly, the association with “social interaction” and “flexibility” was found both in the forward likelihood analysis and the reverse probability analysis, potentially highlighting the relevance of this term.

While the results of our functional decoding analysis are preliminary and exploratory, they suggest initial associations between altered brain structure and terms that are highly relevant in psychotherapeutic treatments of AN. Accordingly, our findings may be interpreted not only from a neurobiological perspective, but also in light of their potential clinical relevance. In this context, recent literature for example underscores the benefits of interventions such as Cognitive Remediation Therapy (CRT) through targeting neuroplasticity-related deficiencies and potentially fostering neuroplastic changes to improve treatment outcomes and patient prognosis (see Keeler et al., 2023 for an overview).

Overall, this data-driven approach offers a more objective interpretation of our meta-analytic results than traditional meta-analyses, which often relied on reverse literature searches of identified brain areas and findings from single studies. By integrating data from all studies linked to AN related terms included in the Neurosynth database, our study takes a step forward and enables us to interpret the associations revealed by our functional decoding approach from a clinical but at the same time more objective perspective. Similar approaches have been applied in other medical conditions, including major depressive disorder (Miller et al., 2015), post-traumatic stress disorder (Pankey et al., 2022), attention deficit hyperactivity disorder, addiction, and borderline personality disorder (Long et al., 2022, Pan et al., 2023). Our results may serve as a starting point for future meta-analyses in AN to validate and refine this functional decoding approach.

#### 4.3.2 Associations between altered brain structure in AN and neurotransmitter systems

Results of our spatial colocalization analysis for neurotransmitter systems did not survive multiple comparison correction and might thus only present initial indications for a negative association between our ALE meta-analytic map of reduced brain volume and the density of serotonin transporters and a positive relationship with noradrenaline transporter availability. These associations could suggest that brain regions with greater serotonin transporter density may appear more resistant to brain volume reduction, while areas with higher noradrenaline transporter availability could be particularly affected. In the literature, both neurotransmitters were discussed as relevant in the context of AN. While some researchers hypothesized the importance of the noradrenaline system (Nunn et al., 2012), findings remain inconsistent (e.g., Hu et al., 2007, Urwin et al., 2002, Urwin et al., 2003), and overall evidence for its involvement in AN pathogenesis is limited. The role of noradrenaline therefore remains unclear and possibly secondary (see Pruccoli et al., 2021 for an overview). Serotonin, in contrast, appears to play an important role in the disease: Many studies observed a disbalance in the serotonin system in acutely ill (e.g., Audenaert et al., 2003, Gauthier et al., 2014) and recovered patients (e.g., Bailer et al., 2004, Frank et al., 2002). Although preliminary, the results of our meta-analysis could potentially help foster research on biological interventions, as serotonergic pathways are being discussed in the context of pharmacological interventions for AN (Frank, 2014, Frank, 2020, Keeler et al., 2021, Strumila et al., 2022). However, the use of selective serotonin reuptake inhibitors (SSRIs) in acutely starved patients did not have the same antidepressive or anxiolytic effects seen in normal weight patients (see e.g., Attia et al., 1998, Ferguson et al., 1999, Strober et al., 1999; see Frank, 2020, Marvanova and Gramith, 2018 for an overview), and even though clinical trials assessing the therapeutic potential of agonistic and antagonistic drug treatments targeting the serotonin system are emerging, little evidence for beneficial effects in AN was shown to date (see Frank, 2020 for a review).

### 4.4 Strengths and limitations

Our comprehensive meta-analysis including 1130 acutely ill patients has the power to contribute to a deeper understanding of brain structural alterations in AN by combining global and regional aspects of brain volume reductions with functional associations. However, a series of limitations apply that are important to consider. First, both types of meta-analyses combine imaging studies that used different scanner types, protocols as well as analysis techniques, and included patient samples with some variability in clinical characteristics, treatment approaches, and geographic origin. This methodological and sample-related diversity inherently involves a risk of heterogeneity between studies, as also reflected by the I^2^ statistics in some contrasts of our global brain volume meta-analyses. Moreover, the criteria for recovery were different in the separate studies as no broad consensus on recovery exists for AN. Thus, we cannot exclude partially altered eating behavior or relatively lower body weight even in the recovered patient population, that might have influenced above findings. In addition to that, our meta-analysis did not allow to control for duration of illness, age of onset or BMI which could also influence the results. Future mega-analyses incorporating individual participant data are needed to disentangle these clinical factors in more depth. Furthermore, interpretations must be made with caution due to the currently still small number of studies in longer-term recovered adolescents with AN. In addition, there were not enough studies to calculate separate ALE meta-analyses for adolescents or recovered patients. We hope that future meta-analyses will be able to perform these analyses once more research has been done in these groups. Besides, including a combination of coordinates for GM volume and CT into our ALE meta-analyses is not standard and should be validated in future studies. Lastly, our meta-analysis does not allow to draw conclusions about causality or the direction of effects. Even though it seems likely that the brain structural alterations are a consequence of the disease, as the brain volume loss is especially pronounced in the acute stage of disease and decreases quickly during recovery, we cannot rule out that some brain alterations did exist before the onset of AN. In particular, the differences between patients and HCs found after longer-term recovery, while appearing to reflect a “scar” of the disease, may have existed prior to disease onset and could represent a predisposition to AN. To clarify this, we would need prospective studies, which, however, are very rare in the neuroimaging field.

## 5 Conclusion

The results of our global brain volume meta-analysis demonstrate that patients with acute AN show remarkably decreased GM and WM and increased CSF. These dramatic volume changes are gradually reduced during recovery. However, significant persistent brain volume reductions in GM in longer-term recovered patients with AN were found, turning significant in our meta-analysis for the first time. Even though reductions in GM volume and CT seem to be widespread and of global nature, consistent patterns of structurally altered brain regions particularly affected including the cingulate gyrus, precentral gyrus and precuneus were detected by the ALE meta-analyses. Functional decoding methods suggest relative preservation from brain volume loss in regions associated with eating, food, threat, and reinforcement. These results, shedding new light on volumetric brain changes in AN, can help us decipher the underlying pathophysiology and the connection between brain and function, though further validation is needed to substantiate these initial observations. Overall, an important implication for clinical practice that can be derived from our meta-analysis is the critical necessity for rapid weight gain in the treatment of AN to prevent persistent brain damages, as from a clinical perspective, treatment resistance or chronicity which are common in AN could be a consequence of continuous structural brain abnormalities.

## CRediT authorship contribution statement

**Lara Keller:** Writing – original draft, Visualization, Validation, Software, Project administration, Methodology, Investigation, Formal analysis, Data curation, Conceptualization. **Leon D. Lotter:** Writing – review & editing, Visualization, Supervision, Software, Methodology, Formal analysis. **Claudia R. Eickhoff:** Writing – review & editing, Supervision, Software, Formal analysis. **Simon B. Eickhoff:** Writing – review & editing, Supervision, Software. **Katharina Otten:** Writing – review & editing, Validation. **Beate Herpertz-Dahlmann:** Writing – review & editing, Supervision, Resources, Funding acquisition. **Jochen Seitz:** Writing – review & editing, Supervision, Resources, Funding acquisition, Conceptualization.

## Declaration of competing interest

The authors declare that they have no known competing financial interests or personal relationships that could have appeared to influence the work reported in this paper.

## Data Availability

Data will be made available on request.
