# Supplementary material for: Structural brain alterations in anorexia nervosa: a global brain volume and anatomical likelihood estimation (ALE) meta-analysis combined with a functional decoding approach
Source: Neuroimage Clin. 2026 Jan 18;49:103950. doi: 10.1016/j.nicl.2026.103950 (PMC12874596; doi:10.1016/j.nicl.2026.103950)
Supplement: Supplementary Data 1 [file mmc1.pdf]

Supplementary Materials

Structural brain alterations in anorexia nervosa: A global brain volume and anatomical likelihood estimation (ALE) meta-analysis combined with a functional decoding approach

Lara Keller, Leon D. Lotter, Claudia R. Eickhoff, Simon B. Eickhoff, Katharina Otten, Beate Herpertz-Dahlmann, Jochen Seitz

Content

1 Supplementary Methods 2
2 Supplementary Tables 3
3 Supplementary Figures 34
4 Detailed information on risk of bias assessment 45
5 References Supplement 54

1 Supplementary Methods

Additional information on ALE methodology

The ALE algorithm used for this meta-analysis was developed by Eickhoff and colleagues (Eickhoff et al., 2009; Eickhoff et al., 2012). First, MNI or TAL coordinates from neuroimaging studies representing the peak locations of significant differences in brain structure between patients with AN and HCs were extracted from the papers identified by our literature search. If papers reported foci for both GM volume and CT, all foci were combined to one single "pseudo experiment" ensuring that every individual participant was only counted once. In the next step, all foci in each experiment were modeled as Gaussian probability distributions to account for the spatial uncertainty associated with the reported foci. The full width at half maximum (FWHM) of these distributions is determined based on between-subject (i.e. the sample size) and between-template (i.e. the stereotactic space) variations. The latter was addressed by converting all coordinates to MNI-152 space as a common stereotactic space to ensure template consistency across studies (Lancaster et al., 2007). As the extent of spatial uncertainty depends highly on the sample size, the ALE algorithm accounts for between-subject variance by applying tighter Gaussian distributions for studies with more participants. By this means, studies with a larger sample size are given more weight and should have increased localizing power in the ALE meta-analysis (Eickhoff et al., 2009). Afterwards, the probabilities of all foci of an experiment were integrated into a single 3D volume leading to a modeled effect map for each experiment (Eickhoff et al., 2009). Afterwards, all modeled effect maps were used to calculate anatomical likelihood estimation scores and combined in a meta-analytic ALE map which highlights regions with consistent local convergence across studies. The ALE map indicates the probability for each voxel that the true center of at least one reported coordinate is located at exactly this position. Next, permutation testing was performed to determine the statistical significance of the observed convergence. It was tested whether the meta-analytic ALE map differed significantly from random spatial permutations by comparing the meta-analytic map with a null distribution which assumes a missing spatial correlation between experiments included in the ALE meta-analysis. As a result, regions showing convergence across studies which is higher than it would have been expected based on the simulation of a random spatial correlation were identified (Eickhoff et al., 2009).

2 Supplementary Tables

Supplementary Table 1. Sources of neurotransmitter maps used for spatial association analyses between anatomical likelihood estimation (ALE) results and neurotransmitter systems.

		**Atlases**				
**Target system**	**Target function**	**Name**	**Tracer**	**Subjects (N)**	**Meag age (years)**	**Source**
**Serotonin**	serotonin receptor 1a	5HT1a	Cumi101	8	28.40	(Beliveau et al., 2017)
	serotonin receptor 1b	5HT1b	P943	65	33.70	(Gallezot et al., 2010)
	serotonin receptor 2a	5HT2a	Cimbi-36	29	22.60	(Beliveau et al., 2017)
	serotonin receptor 4	5HT4	SB207145	59	25.90	(Beliveau et al., 2017)
	serotonin receptor 6	5HT6	GSK215083	30	36.60	(Radhakrishnan et al., 2018)
	Serotonin transporter	5HTT	DASB	100	25.10	(Beliveau et al., 2017)
**Cannabinoid**	cannabinoid receptor 1	CB1	OMAR	77	30.00	(Normandin et al., 2015)
**Dopamine**	dopamine receptor 1	D1	SCH23390	13	33.00	(Kaller et al., 2017)
	dopamine receptor 2	D2	FLB-457	55	32.50	(Sandiego et al., 2015)
	dopamine transporter	DAT	FP-CIT	174	61.00	(Dukart et al., 2018)
	dopamine synthesis	FDOPA	Fluorodopa	12	NA	(Gómez et al., 2018)
**GABA**	GABA receptor A	GABAa	Flumazenil	10	26.60	(Kaulen et al., 2022)
	GABA receptor A5	GABAa5	RO154513	10	25.40	(Lukow et al., 2022)
**Histamine**	histamine receptor 3	H3	GSK189254	8	31.70	(Gallezot et al., 2017)
**Acetylcholine**	α4β2 nicotinic receptor	a4b2	Flubatine	30	33.50	(Hillmer et al., 2016)
	muscarinic receptor 1	M1	LSN3172176	24	40.50	(Naganawa et al., 2021)
	vesicular Ach transporter	VachT	FEOBV	18	66.80	(Aghourian et al., 2017)
**Glutamate**	metabotropic receptor 5	mGluR5	ABP688	73	19.90	(Smart et al., 2019)
	NMDA receptor	NMDA	GE-179	29	41.00	(Galovic et al., 2021)
**Endorphins**	μ receptor	MU	Carfentanil	204	32.30	(Kantonen et al., 2020)
**Noradrenaline**	noradrenaline transporter	NET	MRB	77	33.40	(Ding et al., 2010)

Supplementary Table 2. Overview of all studies included in the global brain volume meta-analysis and the reported brain volume changes in patients with AN_{acute} (A), $AN_{short\text{-}rec}$ (B) and $AN_{longer\text{-}rec}$ (C) compared to healthy controls.

**(A) AN acute** (acutely ill)									
**Study**	**N patients / HCs**	**Age group**	**Age (SD) patients / HCs**	**BMI (SD) patients / HCs**	**Illness duration (SD) patients**	**NOS total score**	**Method**	**GM change**	**WM change**
**Amianto et al., 2013 (Amianto et al., 2013)**	17 / 14	adults	20 y (4) / 24 y (3)	16.00 (1.00) / 21.00 (2.00)	13.0 (8.0) months	7	VBM	-0.2%	-1.4%
**Bär et al., 2015 (Bär et al., 2015)**	26 / 26	adults	22.96 y (4.97) / 24 y (1.92)	16.97 (1.46) / 21.72 (1.5)	22.4 (14.80) months	8	VBM	-4.1%	-0.2%
**Boghi et al., 2011 (Boghi et al., 2011)**	21 / 27	adults	29 y (10) / 30.8 y (8.7)	15.5 (1.75) / 21.9 (1.5)	11.3 (12.10) years	7	VBM	2.0%	-7.7%
**Bomba et al., 2013 (Bomba et al., 2013)**	11 / 8	adolescents	13.6 y (2.8) / 13.3 y (2.4)	12.18 (0.87) / 19.87 (1.45)	14.45 (10.92) months	6	VBM	-10.9%	-6.5%
**Boto et al., 2017** [a] **(Boto et al., 2017)**	20 / 10	adults	27.9 y (10.3) / 26.5 y (3.4)	14.50 (2.20) / 21.8 (3.2)	NA	4	MorphoBox	-7.1%	-6.7%
**Brooks et al., 2011 (Brooks et al., 2011)**	14 / 21	adults	26 y (1.9) / 26 y (2.1)	15.6 (0.4) / 21.4 (0.5)	9.2 (1.90) years	7	VBM	-4.0%	3.5%
**Burkert et al., 2015 (Burkert et al., 2015)**	21 / 21	adults	21.6 y (5.7) / 21.7 y (5.7)	15.8 (1.3) / 20.2 (1.5)	4.4 (3.3) years	8	Freesurfer	-8.8%	-3.4%
**Castro-Fornieles et al., 2009 (Castro-Fornieles et al., 2009)**	12 / 9	adolescents	14.5 y (1.5) / 14.6 y (3.2)	14.8 (2) / NA (NA)	8.3 (3.1) months	6	VBM	-7.7%	-2.7%
**Curzio et al., 2020 (Curzio et al., 2020)**	24 / 24	adolescents	15.2 y (2) / 15.4 y (2.2)	14.5 (1.7) / 21 (2)	16.1 (15.7) months	6	VBM	-2.2%	-2.1%
**D'Agata et al., 2015 (D'Agata et al., 2015)**	21 / 17	adults	21 y (5) / 23 y (4)	16.1 (0.9) / 21.5 (2.3)	less than 2 years	7	VBM	-1.9%	-0.6%
**Doose et al., 2023 (Doose et al., 2023)**	30 / 30	adolescents	16.1 y (2.2) / 16.2 y (1.8)	14.1 (1.4) / 20.8 (2.1)	11.4 (9.0) months	8	Freesurfer	-8.2%	-1.8%

**Favaro et al., 2015 (Favaro et al., 2015)**	38 / 38	adults	26.1 y (7.2) / 25.3 y (6.3)	15.8 (1.8) / 21.7 (2.9)	78.6 (81.30) months	6	Freesurfer	-3.1%	1.1%
**Fonville et al., 2014 (Fonville et al., 2014)**	31 / 31	adults	23 y (10) / 25 y (4)	15.8 (1.4) / 21.8 (1.8)	7 (10) years	9	VBM	-1.5%	1.1%
**Frank et al., 2013a (Frank et al., 2013a)**	19 / 24	adults	23.1 y (5.8) / 27.4 y (6.3)	16 (1.1) / 21.6 (1.3)	NA	7	VBM	-0.3%	-1.8%
**Frank et al., 2013b (Frank et al., 2013b)**	19 / 22	adolescents	15.4 y (1.4) / 14.8 y (1.8)	16.2 (1.1) / 21.3 (1.9)	NA	6	VBM	2.1%	0.2%
**Friederich et al., 2012 (Friederich et al., 2012)**	12 / 14	adults	24.3 y (6.2) / 25.6 y (3.7)	15.9 (1.6) / 21.1 (1.5)	6.3 (4.40) years	8	VBM	-4.6%	0.1%
**Fujisawa et al., 2015 (Fujisawa et al., 2015)**	20 / 14	adolescents	14.2 y (1.8) / 14.9 y (1.6)	14.35 (2.084) / NA (NA)	23.55 (17.02) months	7	VBM	-10.0%	n.a.
**Gaudio et al., 2011 (Gaudio et al., 2011)**	16 / 16	adolescents	15.2 y (1.7) / 15.1 y (1.5)	14.2 (1.4) / 20.2 (1.6)	5.3 (3.2) months	6	VBM	-8.2%	n.a.
**Gaudio et al., 2017 (Gaudio et al., 2017)**	14 / 15	adolescents	15.7 y (1.6) / 16.3 y (1.5)	16.2 (1.2) / 21.1 (1.9)	4.9 (1.8) months	8	VBM	-2.6%	-0.9%
**Geisler et al., 2022 (Geisler et al., 2022)**	96 / 96	adolescents	16.3 y (3.3) / 17.2 y (3.3)	14.7 (1.4) / 20.7 (2.00)	1.89 (1.67) years	8	Freesurfer	n.a.	-2.4%
**Halls et al., 2022 (Halls et al., 2022)**	57 / 68	adults	19.4 y (2.83) / 19.41 y (3.37)	16.38 (1.39) / 22.89 (3.36)	3.69 (2.81) years	7	Freesurfer	-3.1%	-1.8%
**Joos et al., 2010 (Joos et al., 2010)**	12 / 18	adults	25 y (4.8) / 26.9 y (5.7)	16 (1.2) / 21.2 (2)	4.7 (3.60) years	8	VBM	-4.0%	-2.8%
**Katzman et al., 1996 (Katzman et al., 1996)**	13 / 8	adolescents	15.2 y (1.2) / 15.4 y (1.5)	15.6 (1.7) / 22.9 (2.3)	11.3 (6.0) months	6	BIS	-6.2%	-3.6%
**Kaufmann et al., 2020 (Kaufmann et al., 2020)**	24 / 30	adults	22.3 y (4.1) / 24.2 y (3.4)	14.25 (1.08) / 20.81 (1.77)	5.59 (4.30) years	8	Freesurfer	-5.1%	-2.8%
**Khalsa et al., 2016 (Khalsa et al., 2016)**	12 / 30	adults	19.4 y (7.3) / 22.5 y (4.8)	16.2 (2) / 22.6 (3.4)	5.4 years	5	VBM	0.1%	-3.7%
**King et al., 2015 (King et al., 2015)**	40 / 40	adolescents	15.9 y (2.5) / 16.2 y (2.9)	14.8 (1.3) / 20.8 (2.7)	18.0 (26.0) months	8	Freesurfer	-4.5%	n.a.

**Kohmura et al., 2017 (Kohmura et al., 2017)**	23 / 29	adults	28.5 y (6.7) / 28.2 y (7.0)	13.2 (1.5) / 21.5 (3.3)	10.5 (6.20) years	7	VBM	-8.2%	0.2%
**Lloyd et al., 2023[b] (Lloyd et al., 2023) – study 1**	23 / 20	adults	19.26 y (2.49) / 20.7 y (2.98)	17.36 (0.92) (N=8); BMI percentile: 3.92 (7.42) (N=15) / 21.47 (1.67) (N=13); BMI percentile: 35.26 (16.7) (N=7)	52.48 (37.72) months	6	Freesurfer	-4.9%	-0.8%
**Lloyd et al., 2023[b] (Lloyd et al., 2023) – study 2**	26 / 25	adults	19.35 y (3.49) / 19.36 y (3.01)	15.46 (2.25) (N=13); BMI percentile: 7.64 (6.77) (N=13) / 21.24 (1.61) (N=9); BMI percentile: 50.72 (19.23) (N=16)	38.19 (28.42) months	6	Freesurfer	-3.7%	-0.7%
**Lloyd et al., 2023[b] (Lloyd et al., 2023) – study 3**	16 / 13	adults	26.88 y (6.93) / 22.08 y (2.47)	15.32 (1.66) (N=14); BMI percentile: 7.65 (3.32) (N=2) / 21.51 (2.33) (N=11); BMI percentile: 17.75 (16.48) (N=2)	144.5 (82.34) months	6	Freesurfer	-2.6%	4.9%
**Lloyd et al., 2023[b] (Lloyd et al., 2023) – study 4**	30 / 35	adults	26.93 (6.07) / 25.86 y (5.35)	16.00 (1.65) (N=25); BMI percentile: 7.62 (7.11) (N=5) / BMI: 21.00 (1.60) (N=32); BMI percentile: 22.13 (5.45) (N=3)	118.53 (79.39) months	6	Freesurfer	-5.3%	-2.0%
**Mishima et al., 2021 (Mishima et al., 2021)**	35 / 35	adults	36.3 y (10) / 36 y (9.6)	14.2 (2.5) / 21 (2.9)	15.7 (9.00) years	8	VBM	-6.5%	-3.9%
**Myrvang et al., 2018 (Myrvang et al., 2018)**	30 / 28	adolescents	15.8 y (1.7) / 16.2 y (1.9)	16.3 (1.6) / 21.8 (3.1)	NA	7	Freesurfer	-7.7%	-4.5%
**Nickel et al., 2018 (Nickel et al., 2018)**	34 / 41	adults	23.8 y (4.3) / 23.6 y (3.8)	16.1 (1.4) / 22.3 (2.4)	6.6 (3.70) years	7	VBM	-6.3%	-5.5%
**Olivo et al., 2018 (Olivo et al., 2018)**	22 / 38	adolescents	14.9 y (1.6) / 14.7 y (1.3)	19.3 (2) / 20.7 (2.3)	0.66 (0.4) years	8	VBM	-0.5%	-1.4%
**Roberto et al., 2011 (Roberto et al., 2011)**	32 / 21	adults	26.9 y (6.4) / 25 y (3.2)	16.03 (1.59) / 20.82 (1.22)	10.15 (6.23) years	9	VBM	-5.2%	-2.3%
**Scaife et al., 2017 (Scaife et al., 2017)**	12 / 16	adults	29.4 y (6) / 24.3 y (5.7)	15.4 (1.9) / 21.2 (2)	10.3 (5.2) years	5	FSL-VBM	-6.3%	-0.4%
**Seitz et al., 2015 (Seitz et al., 2015)**	56 / 50	adolescents	15.5 y (1.7) / 15.8 y (1.7)	15.13 (1.37) / 21.37 (3.32)	11.43 (8.31) months	7	Freesurfer	-7.1%	-4.7%
**Suchan et al., 2010 (Suchan et al., 2010)**	15 / 15	adults	26.8 y (8.4) / 29.5 y (8.2)	16.00 (1.30) / 22.00 (2.10)	5.5 (5.00) years	6	VBM	0.0%	-3.3%
**Swayze et al., 2003 (Swayze et al., 2003)**	18 / 18	adults	25.1 y (7.3) / 26 y (7.6)	13.4 (2.1) / 24.2 (3.3)	NA	6	BRAINS	-4.2%	-11.0%

**Via et al., 2014 (Via et al., 2014)**	19 / 19	adults	28.4 y (9.6) / 28.6 y (8.6)	17.03 (1.09) / 21.09 (1.8)	78.32 (72.37) months	8	VBM	-1.4%	-3.5%
**Yu et al., 2024 (Yu et al., 2024)**	64 / 56	adults	21.78 y (2.1) / 22.72 y (0.62)	16.62 (1.80) / 24.91 (4.72)	NA	6	VBM	-4.0%	-0.5%
**Yue et al., 2018 (Yue et al., 2018)**	35 / 20	adults	19.3 y (4.1) / 21.4 y (3.3)	15.34 (1.96) / 20.92 (2.3)	33.23 (32.24) months	6	Freesurfer	-6.0%	n.a.

(B) AN short-rec										
**Study**	**N patients / HCs**	**Age group**	**Age (SD) patients / HCs**	**BMI (SD) patients / HCs**	**Illness duration (SD) patients**	**NOS total score**	**Method**	**Recovery length**	**GM change**	**WM change**
**Bomba et al., 2015 (Bomba et al., 2015)**	11 / 8	adolescents	13.6 y (2.8) / 13.3 y (2.4)	18.87 (1.86) / 19.87 (1.45)	14.45 (10.92) months	6	VBM	weight recovered	-4.3%	-2.9%
**Brodrick et al., 2021[a] (Brodrick et al., 2021)**	32 / 41	adults	29.4 y (8.3) / 26.7 y (6.2)	22.09 (2.33) / 23.17 (3.12)	NA	6	Freesurfer	7.5 months	-1.2%	-0.1%
**Castro-Fornieles et al., 2009 (Castro-Fornieles et al., 2009)**	12 / 9	adolescents	14.5 y (1.5) + 7 months until weight restoration / 14.6 y (3.2) + 7 months until follow-up	18.8 (0.4) / NA (NA)	8.3 (3.1) months	6	VBM	7 months post admission	-1.6%	-0.8%
**Frank et al., 2013a (Frank et al., 2013a)**	24 / 24	adults	30.3 y (8.1) / 27.4 y (6.3)	20.8 (2.4) / 21.6 (1.3)	NA	7	VBM	at least 1.0 y (no mean reported)	0.9%	-0.8%
**Katzman et al., 1997 (Katzman et al., 1997)**	6 / 16	adolescents	17 y (1.4) / 17.4 y (3.5)	23 (3.7) / 22.5 (3.4)	22.5 (10.3) months	7	BIS	16.3 (3.7) months	-4.2%	-0.5%
**Kaufmann et al., 2020 (Kaufmann et al., 2020)**	26 / 30	adults	22.3 y (4.1) + 22.0 (16.3) weeks until end of treatment / 24.2 y (3.4) + 25.9 (8.7) weeks until follow-up	18.41 (0.45) / 20.38 (1.56)	5.59 (4.30) years	8	Freesurfer	weight recovered	0.7%	-1.0%
**Khalsa et al., 2016 (Khalsa et al., 2016)**	20 / 30	adults	22.4 y (3.9) / 22.5 y (4.8)	20.2 (1.6) / 22.6 (3.4)	6.6 years	5	VBM	weight recovered	1.4%	0.0%
**Lázaro et al., 2013 (Lázaro et al., 2013)**	35 / 17	adolescents	16.3 y (1.3) / 16.7 y (1.5)	19.3 (1.1) / NA (NA)	NA	7	VBM	weight recovered	0.1%	-0.5%
**Mainz et al., 2012 (Mainz et al., 2012)**	18 / 19	adolescents	15.7 y (1.5) + on average 105 (39) days until weight restoration/ 15.6 y (1.9)	18.1 (1) / 21.8 (2.7)	mean duration of illness < 1 year	8	VBM	weight recovered	-12.1%	0.7%
**Roberto et al., 2011 (Roberto et al., 2011)**	32 / 21	adults	26.9 y (6.4) + 50.3 (19.1) days until weight restoration /	20.01 (0.59) / 20.6 (1.17)	10.15 (6.23) years	9	VBM	weight recovered	-4.2%	-1.2%

			25 y (3.2) + 51.7 (37.7) days until follow-up							
**Swayze et al., 2003 (Swayze et al., 2003)**	13 / 18	adults	25.1 y (7.3) + on average 107,2 (39,7) days until weight restoration / 26 y (7,6)	19.99 (NA) / 24.2 (3.3)	NA	6	BRAINS	weight recovered	-1.9%	-3.5%

(C) AN longer-rec										
**Study**	**N patients / HCs**	**Age group**	**Age (SD) patients / HCs**	**BMI (SD) patients / HCs**	**Illness duration (SD) patients**	**NOS total score**	**Method**	**Recovery length (mean)**	**GM change**	**WM change**
**Bang et al., 2016 (Bang et al., 2016)**	22 / 22	adults	27.3 y (5.1) / 26.1 y (4.6)	20.39 (1.66) / 21.85 (1.76)	32.86 (27.47) months	8	VBM	4.3 y	-1.3%	1.9%
**Favaro et al., 2015 (Favaro et al., 2015)**	20 / 38	adults	26.3 y (7.1) / 25.3 y (6.3)	19.6 (1.6) / 21.7 (2.9)	45.7 (65.0) months	6	Freesurfer	3.8 y	-0.1%	-0.2%
**Friederich et al., 2012 (Friederich et al., 2012)**	13 / 14	adults	25 y (4.8) / 25.6 y (3.7)	19.5 (1.4) / 21.1 (1.5)	5.7 (3.60) years	8	VBM	5.7 y	-0.9%	-2.9%
**Joos et al., 2011 (Joos et al., 2011)**	5 / 18	adults	29.6 y (5.1) / 26.9 y (5.7)	19.9 (1.5) / 21.2 (2)	7.2 (6.0) years	5	VBM	5.2 y	-1.6%	-0.3%
**King et al., 2015 (King et al., 2015)**	34 / 34	adolescents	22.7 y (2.9) / 22.3 y (2.8)	21 (1.9) / 21.6 (2.1)	NA	8	Freesurfer	4.4 y	-3.8%	n.a.
**Lambe et al., 1997 (Lambe et al., 1997)**	12 / 18	adults	18.9 y (6.9) / 19.9 y (7.1)	20.5 (2.3) / 21.9 (3.4)	14.0 (10.0) months	8	BIS	4.7 y	-2.6%	-0.3%
**Mühlau et al., 2007 (Mühlau et al., 2007)**	22 / 37	adults	23.7 y (6) / 24.7 y (4.3)	19.7 (1.6) / 20.7 (1.8)	5.2 (5.10) years	6	VBM	1.6 y	-3.3%	0.0%
**Nickel et al., 2018 (Nickel et al., 2018)**	24 / 41	adults	27.1 y (7) / 23.6 y (3.8)	20.6 (1.3) / 22.3 (2.4)	7.2 (4.7) years	7	VBM	4.3 y	-3.2%	-0.7%
**Oliva et al., 2020 (Oliva et al., 2020)**	15 / 15	adults	25.9 y (6.2) / 25.2 y (1)	20.1 (2.04) / 21.32 (2.45)	38.4 (38) months	7	VBM	6.2 y	-1.1%	-0.4%
**Pfuhl et al., 2016 (Pfuhl et al., 2016)**	31 / 31	adults	22.5 y (3) / 22.5 y (2.9)	21.09 (1.91) / 21.34 (2.18)	NA	8	Freesurfer	4.8 y	n.a.	-6.1%
**Scaife et al., 2017 (Scaife et al., 2017)**	14 / 16	adults	27 y (6.5) / 24.3 y (5.7)	20.9 (1.6) / 21.2 (2)	5.8 (4.2) years	5	FSL-VBM	4.7 y	-1.9%	0.8%
**Wagner et al., 2006 (Wagner et al., 2006)**	30 / 31	adults	25.6 y (6.3) / 26.8 y (7.3)	21.2 (NA) / 21.9 (2)	NA	5	VBM	2.8 y	0.2%	-2.0%
**Zucker et al., 2017 (Zucker et al., 2017)**	21 / 20	adolescents	17.5 y (1.7) / 17.4 y (1.9)	20.01 (2.61) / 21.34 (3.0)	3.14 (2.03) years	8	VBM	3.3 y	-0.5%	0.0%

For VBM studies, brain volume scores corrected for total intracranial volume were used to calculate the volume change in percent, whereas for Freesurfer studies, uncorrected volume scores were utilized. [a] Reported brain volume scores were used to determine the volume change in percent but were not included in the RevMan analysis as no standard deviation for the volume scores was provided in the article. [b] Since the study included both adolescents and adults, the classification of participants as "adults" was based on the mean age. BMI information reported for participants over 19 years, BMI percentile information presented for participants under 19 years. Abbreviations: *AN,* Anorexia nervosa; *BIS,* Brain Imaging Software; *BRAINS,* Brain Research: Analysis of Images, Networks, and Systems; *GM,* Gray matter; *NOS*, Newcastle-Ottawa Scale; *WM,* White matter; *HCs,* Healthy controls; *y,* Years; *n.a.,* Not available; *VBM, V*oxel-based morphometry; *short-rec,* Short-term weight recovered; *longer-rec,* Longer-term recovered (> 1.5 years).

Supplementary Table 3. Overview of all studies eligible for the ALE meta-analysis as revealed by our literature search. Supplementary Table 3 (A) provides the extracted MNI or TAL coordinates for each experiment and further study characteristics. Supplementary Table 3 (B) displays details for a further characterization of the included samples (age, BMI, illness duration) and the rating of methodological quality assessed via the modified Newcastle-Ottawa scale (NOS).

3 (A) - Experiment	n total	X	Y	Z	Space	Contrast	n AN	n HC	Age group	AN status	Measure	Software
Amianto 2013 (Amianto et al., 2013)	31	12	-38	52	MNI	AN>HC	17	14	adults	acute	gmv	FSL-VBM
	31	-6	-17	64	MNI	AN<HC	17	14	adults	acute	gmv	FSL-VBM
	31	-36	-48	-36	MNI	AN<HC	17	14	adults	acute	gmv	FSL-VBM
	31	52	-70	-28	MNI	AN<HC	17	14	adults	acute	gmv	FSL-VBM
	31	10	-18	0	MNI	AN<HC	17	14	adults	acute	gmv	FSL-VBM
	31	22	-18	48	MNI	AN<HC	17	14	adults	acute	gmv	FSL-VBM
	31	36	-70	-4	MNI	AN<HC	17	14	adults	acute	gmv	FSL-VBM
	31	-24	-22	-32	MNI	AN<HC	17	14	adults	acute	gmv	FSL-VBM
	31	39	-30	38	MNI	AN<HC	17	14	adults	acute	gmv	FSL-VBM
	31	-28	-6	-22	MNI	AN<HC	17	14	adults	acute	gmv	FSL-VBM
Bär 2015 (Bär et al., 2015)	52	8	-43	42	MNI	AN<HC	26	26	adults	acute	gmv	VBM8 - SPM8
	52	5	-61	43	MNI	AN<HC	26	26	adults	acute	gmv	VBM8 - SPM8
	52	-5	-26	44	MNI	AN<HC	26	26	adults	acute	gmv	VBM8 - SPM8
	52	12	-26	41	MNI	AN<HC	26	26	adults	acute	gmv	VBM8 - SPM8
	52	2	0	56	MNI	AN<HC	26	26	adults	acute	gmv	VBM8 - SPM8
	52	8	15	61	MNI	AN<HC	26	26	adults	acute	ct	Freesurfer
	52	-23	23	51	MNI	AN<HC	26	26	adults	acute	ct	Freesurfer
	52	-31	-87	13	MNI	AN<HC	26	26	adults	acute	ct	Freesurfer
	52	36	-49	57	MNI	AN<HC	26	26	adults	acute	ct	Freesurfer
	52	-37	1	48	MNI	AN<HC	26	26	adults	acute	ct	Freesurfer
	52	40	-76	28	MNI	AN<HC	26	26	adults	acute	ct	Freesurfer
	52	47	-27	56	MNI	AN<HC	26	26	adults	acute	ct	Freesurfer
	52	-20	-60	19	MNI	AN<HC	26	26	adults	acute	ct	Freesurfer
	52	39	3	40	MNI	AN<HC	26	26	adults	acute	ct	Freesurfer
	52	-31	-55	62	MNI	AN<HC	26	26	adults	acute	ct	Freesurfer
	52	57	-35	15	MNI	AN<HC	26	26	adults	acute	ct	Freesurfer
Boghi 2011 (Boghi et al., 2011)	48	43	-15	22	TAL	AN<HC	21	27	adults	acute	gmv	SPM 2
	48	47	-54	44	TAL	AN<HC	21	27	adults	acute	gmv	SPM 2
	48	40	-62	-14	TAL	AN<HC	21	27	adults	acute	gmv	SPM 2
	48	5	10	6	TAL	AN<HC	21	27	adults	acute	gmv	SPM 2
	48	2	-3	-11	TAL	AN<HC	21	27	adults	acute	gmv	SPM 2
	48	-28	-53	-8	TAL	AN<HC	21	27	adults	acute	gmv	SPM 2
	48	-32	-42	-3	TAL	AN<HC	21	27	adults	acute	gmv	SPM 2

	48	34	-43	-43	TAL	AN<HC	21	27	adults	acute	gmv	SPM 2
	48	-3	5	6	TAL	AN<HC	21	27	adults	acute	gmv	SPM 2
	48	0	-37	66	TAL	AN<HC	21	27	adults	acute	gmv	SPM 2
	48	2	-30	53	TAL	AN<HC	21	27	adults	acute	gmv	SPM 2
	48	-42	0	33	TAL	AN<HC	21	27	adults	acute	gmv	SPM 2
	48	44	-86	7	TAL	AN<HC	21	27	adults	acute	gmv	SPM 2
	48	-47	-52	7	TAL	AN<HC	21	27	adults	acute	gmv	SPM 2
	48	-3	-2	-10	TAL	AN<HC	21	27	adults	acute	gmv	SPM 2
	48	40	26	11	TAL	AN<HC	21	27	adults	acute	gmv	SPM 2
	48	-16	-59	-26	TAL	AN<HC	21	27	adults	acute	gmv	SPM 2
	48	55	-11	46	TAL	AN<HC	21	27	adults	acute	gmv	SPM 2
	48	-19	-68	41	TAL	AN<HC	21	27	adults	acute	gmv	SPM 2
Bomba 2015 (Bomba et al., 2015)	19	26	-30	61	MNI	AN<HC	11	8	adolescents	acute	gmv	SPM5
	19	23	-82	-45	MNI	AN<HC	11	8	adolescents	acute	gmv	SPM5
	19	-33	-30	45	MNI	AN<HC	11	8	adolescents	acute	gmv	SPM5
	19	29	-64	48	MNI	AN<HC	11	8	adolescents	acute	gmv	SPM5
	19	-33	-27	4	MNI	AN<HC	11	8	adolescents	acute	gmv	SPM5
Brooks 2011 (Brooks et al., 2011)	35	-3	-52	-16	TAL	AN<HC	14	21	adults	acute	gmv	SPM5
	35	24	-36	4	TAL	AN<HC	14	21	adults	acute	gmv	SPM5
	35	31	13	-12	TAL	AN<HC	14	21	adults	acute	gmv	SPM5
	35	-25	-93	4	TAL	AN<HC	14	21	adults	acute	gmv	SPM5
	35	4	-45	32	TAL	AN<HC	14	21	adults	acute	gmv	SPM5
	35	34	45	28	TAL	AN>HC	14	21	adults	acute	gmv	SPM5
Cascino 2020 (Cascino et al., 2020)	57	-52.9	4.3	-13.7	TAL	AN<HC	22	35	adults	acute	ct	Freesurfer
	57	13.2	-77.2	-1.3	TAL	AN<HC	22	35	adults	acute	ct	Freesurfer
	57	-8	-7.4	47	TAL	AN>HC	22	35	adults	acute	ct	Freesurfer
	57	8.9	-2.9	49.3	TAL	AN>HC	22	35	adults	acute	ct	Freesurfer
Castro-Fornieles 2009 - 1 (Castro-Fornieles et al., 2009)	21	68	-32	3	MNI	AN<HC	12	9	adolescents	acute	gmv	VBM2 - SPM5
	21	32	-50	69	MNI	AN<HC	12	9	adolescents	acute	gmv	VBM2 - SPM5
	21	-45	-48	61	MNI	AN<HC	12	9	adolescents	acute	gmv	VBM2 - SPM5
Castro-Fornieles 2009 - 2 (Castro-Fornieles et al., 2009)	21	3	-10	54	MNI	AN<HC	12	9	adolescents	short-rec	gmv	VBM2 - SPM5
Castro-Fornieles 2021 (Castro-Fornieles et al., 2021)	43	45	-21.4	40.4	TAL	AN<HC	15	28	adults	longer-rec	ct	Freesurfer
Collantoni 2024 (Collantoni et al., 2024)	90	20.9	62	3.1	TAL	AN<HC	38	52	adolescents	short-rec	ct	Freesurfer
	90	-23.1	-69.3	29.1	TAL	AN<HC	38	52	adolescents	short-rec	ct	Freesurfer
D'Agata2015 (D’Agata et al., 2015)	38	-24	-54	-26	MNI	AN<HC	21	17	adults	acute	gmv	FSL-VBM
	38	24	-50	-26	MNI	AN<HC	21	17	adults	acute	gmv	FSL-VBM
	38	-4	-16	64	MNI	AN<HC	21	17	adults	acute	gmv	FSL-VBM
De la Cruz 2021 (de la Cruz et al., 2021)	48	18	-77	43	MNI	AN<HC	22	26	adults	acute	ct	Freesurfer
	48	44	-60	22	MNI	AN<HC	22	26	adults	acute	ct	Freesurfer

	48	-15	-63	29	MNI	AN<HC	22	26	adults	acute	ct	Freesurfer
	48	-31	-75	33	MNI	AN<HC	22	26	adults	acute	ct	Freesurfer
Favaro 2015 (Favaro et al., 2015)	76	-20	-62	62	MNI	AN<HC	38	38	adults	acute	ct	Freesurfer
	76	-17	6	64	MNI	AN<HC	38	38	adults	acute	ct	Freesurfer
	76	21	-44	60	MNI	AN<HC	38	38	adults	acute	ct	Freesurfer
	76	22	-69	19	MNI	AN<HC	38	38	adults	acute	ct	Freesurfer
	76	33	14	50	MNI	AN<HC	38	38	adults	acute	ct	Freesurfer
	76	20	-67	-6	MNI	AN<HC	38	38	adults	acute	ct	Freesurfer
	76	20	17	58	MNI	AN<HC	38	38	adults	acute	ct	Freesurfer
	76	53	-46	25	MNI	AN<HC	38	38	adults	acute	ct	Freesurfer
Fonville 2014 (Fonville et al., 2014)	62	26	-56	-34	MNI	AN<HC	31	31	adults	acute	gmv	FSL-VBM
	62	28	-50	-16	MNI	AN<HC	31	31	adults	acute	gmv	FSL-VBM
	62	-28	-56	-36	MNI	AN<HC	31	31	adults	acute	gmv	FSL-VBM
	62	-20	-66	34	MNI	AN<HC	31	31	adults	acute	gmv	FSL-VBM
	62	-2	-54	54	MNI	AN<HC	31	31	adults	acute	gmv	FSL-VBM
	62	30	-90	28	MNI	AN<HC	31	31	adults	acute	gmv	FSL-VBM
	62	8	-84	26	MNI	AN<HC	31	31	adults	acute	gmv	FSL-VBM
	62	-24	-92	20	MNI	AN<HC	31	31	adults	acute	gmv	FSL-VBM
	62	20	12	46	MNI	AN<HC	31	31	adults	acute	gmv	FSL-VBM
	62	12	-68	24	MNI	AN<HC	31	31	adults	acute	gmv	FSL-VBM
Frank 2013a - 1 (Frank et al., 2013a)	43	-6	29	-26	MNI	AN>HC	19	24	adults	acute	gmv	VBM8 - SPM8
	43	30	14	-12	MNI	AN>HC	19	24	adults	acute	gmv	VBM8 - SPM8
	43	42	9	4	MNI	AN>HC	19	24	adults	acute	gmv	VBM8 - SPM8
Frank 2013a - 2 (Frank et al., 2013a)	48	-20	-3	18	MNI	AN<HC	24	24	adults	short-rec	gmv	VBM8 - SPM8
	48	20	0	12	MNI	AN<HC	24	24	adults	short-rec	gmv	VBM8 - SPM8
	48	23	0	15	MNI	AN<HC	24	24	adults	short-rec	gmv	VBM8 - SPM8
	48	21	-3	19	MNI	AN<HC	24	24	adults	short-rec	gmv	VBM8 - SPM8
	48	-6	29	-26	MNI	AN>HC	24	24	adults	short-rec	gmv	VBM8 - SPM8
	48	42	9	4	MNI	AN>HC	24	24	adults	short-rec	gmv	VBM8 - SPM8
Frank 2013b (Frank et al., 2013b)	41	-33	60	9	MNI	AN>HC	19	22	adolescents	acute	gmv	VBM8 - SPM8
	41	20	-34	-15	MNI	AN>HC	19	22	adolescents	acute	gmv	VBM8 - SPM8
	41	27	-10	-36	MNI	AN>HC	19	22	adolescents	acute	gmv	VBM8 - SPM8
	41	-32	-7	-41	MNI	AN>HC	19	22	adolescents	acute	gmv	VBM8 - SPM8
	41	29	-27	-8	MNI	AN>HC	19	22	adolescents	acute	gmv	VBM8 - SPM8
	41	-33	-19	-11	MNI	AN>HC	19	22	adolescents	acute	gmv	VBM8 - SPM8
	41	41	5	7	MNI	AN>HC	19	22	adolescents	acute	gmv	VBM8 - SPM8
	41	18	-10	-29	MNI	AN>HC	19	22	adolescents	acute	gmv	VBM8 - SPM8
	41	-21	-13	-27	MNI	AN>HC	19	22	adolescents	acute	gmv	VBM8 - SPM8
	41	-5	30	-26	MNI	AN>HC	19	22	adolescents	acute	gmv	VBM8 - SPM8

	41	-9	30	-26	MNI	AN>HC	19	22	adolescents	acute	gmv	VBM8 - SPM8
	41	-6	53	-24	MNI	AN>HC	19	22	adolescents	acute	gmv	VBM8 - SPM8
Friederich 2012 - 1 (Friederich et al., 2012)	26	37	3	-16	MNI	AN<HC	12	14	adults	acute	gmv	SPM5
	26	28	1	-12	MNI	AN<HC	12	14	adults	acute	gmv	SPM5
	26	-8	-8	-13	MNI	AN<HC	12	14	adults	acute	gmv	SPM5
	26	-18	0	-12	MNI	AN<HC	12	14	adults	acute	gmv	SPM5
	26	-38	-1	-18	MNI	AN<HC	12	14	adults	acute	gmv	SPM5
	26	6	-15	42	MNI	AN<HC	12	14	adults	acute	gmv	SPM5
	26	-6	4	54	MNI	AN<HC	12	14	adults	acute	gmv	SPM5
	26	11	-25	45	MNI	AN<HC	12	14	adults	acute	gmv	SPM5
Friederich 2012 - 2 (Friederich et al., 2012)	27	6	16	27	MNI	AN<HC	13	14	adults	longer-rec	gmv	SPM5
	27	5	-2	64	MNI	AN<HC	13	14	adults	longer-rec	gmv	SPM5
	27	51	7	15	MNI	AN<HC	13	14	adults	longer-rec	gmv	SPM5
	27	58	11	14	MNI	AN<HC	13	14	adults	longer-rec	gmv	SPM5
Fujisawa 2015 (Fujisawa et al., 2015)	34	-52	30	24	MNI	AN<HC	20	14	adolescents	acute	gmv	SPM8
	34	58	30	20	MNI	AN<HC	20	14	adolescents	acute	gmv	SPM8
Gaudio 2011 (Gaudio et al., 2011)	32	-7	-41	31	TAL	AN<HC	16	16	adolescents	acute	gmv	SPM2
	32	0	-54	40	TAL	AN<HC	16	16	adolescents	acute	gmv	SPM2
	32	-38	-48	43	TAL	AN<HC	16	16	adolescents	acute	gmv	SPM2
Gaudio 2017 (Gaudio et al., 2017)	29	-32	2	55	MNI	AN<HC	14	15	adolescents	acute	gmv	VBM8 - SPM8
	29	-26	-7	64	MNI	AN<HC	14	15	adolescents	acute	gmv	VBM8 - SPM8
	29	3	-12	46	MNI	AN<HC	14	15	adolescents	acute	gmv	VBM8 - SPM8
	29	-3	-25	46	MNI	AN<HC	14	15	adolescents	acute	gmv	VBM8 - SPM8
	29	11	-30	46	MNI	AN<HC	14	15	adolescents	acute	gmv	VBM8 - SPM8
	29	-48	-22	45	MNI	AN<HC	14	15	adolescents	acute	gmv	VBM8 - SPM8
	29	-53	-13	48	MNI	AN<HC	14	15	adolescents	acute	gmv	VBM8 - SPM8
	29	29	-9	67	MNI	AN<HC	14	15	adolescents	acute	gmv	VBM8 - SPM8
	29	47	-78	10	MNI	AN<HC	14	15	adolescents	acute	gmv	VBM8 - SPM8
	29	45	-84	0	MNI	AN<HC	14	15	adolescents	acute	gmv	VBM8 - SPM8
	29	48	-78	-11	MNI	AN<HC	14	15	adolescents	acute	gmv	VBM8 - SPM8
	29	26	-27	58	MNI	AN<HC	14	15	adolescents	acute	gmv	VBM8 - SPM8
	29	41	-19	49	MNI	AN<HC	14	15	adolescents	acute	gmv	VBM8 - SPM8
	29	60	24	7	MNI	AN<HC	14	15	adolescents	acute	gmv	VBM8 - SPM8
	29	51	35	0	MNI	AN<HC	14	15	adolescents	acute	gmv	VBM8 - SPM8
	29	45	-73	-38	MNI	AN<HC	14	15	adolescents	acute	gmv	VBM8 - SPM8
	29	51	-63	-36	MNI	AN<HC	14	15	adolescents	acute	gmv	VBM8 - SPM8
	29	39	-66	-47	MNI	AN<HC	14	15	adolescents	acute	gmv	VBM8 - SPM8
	29	-38	-67	-47	MNI	AN<HC	14	15	adolescents	acute	gmv	VBM8 - SPM8
	29	-47	-67	-38	MNI	AN<HC	14	15	adolescents	acute	gmv	VBM8 - SPM8

	29	-45	-58	-44	MNI	AN<HC	14	15	adolescents	acute	gmv	VBM8 - SPM8
	29	63	-54	-6	MNI	AN<HC	14	15	adolescents	acute	gmv	VBM8 - SPM8
	29	-41	-18	54	MNI	AN<HC	14	15	adolescents	acute	gmv	VBM8 - SPM8
	29	-14	2	-20	MNI	AN<HC	14	15	adolescents	acute	gmv	VBM8 - SPM8
	29	-54	-69	-5	MNI	AN<HC	14	15	adolescents	acute	gmv	VBM8 - SPM8
	29	56	12	-20	MNI	AN<HC	14	15	adolescents	acute	gmv	VBM8 - SPM8
	29	50	17	-12	MNI	AN<HC	14	15	adolescents	acute	gmv	VBM8 - SPM8
	29	27	11	54	MNI	AN<HC	14	15	adolescents	acute	gmv	VBM8 - SPM8
Joos 2010 (Joos et al., 2010)	30	12	54	-1	MNI	AN<HC	12	18	adults	acute	gmv	SPM8
	30	-2	19	32	MNI	AN<HC	12	18	adults	acute	gmv	SPM8
	30	55	-2	13	MNI	AN<HC	12	18	adults	acute	gmv	SPM8
	30	-10	-59	31	MNI	AN<HC	12	18	adults	acute	gmv	SPM8
	30	-37	-65	31	MNI	AN<HC	12	18	adults	acute	gmv	SPM8
	30	-50	-34	60	MNI	AN<HC	12	18	adults	acute	gmv	SPM8
	30	42	-73	-56	MNI	AN<HC	12	18	adults	acute	gmv	SPM8
Kaufmann 2020 - 1 (Kaufmann et al., 2020)	54	-34.60	-69.70	45.70	MNI	AN<HC	24	30	adults	acute	ct	Freesurfer
	54	11.00	-86.40	34.80	MNI	AN<HC	24	30	adults	acute	ct	Freesurfer
Kaufmann 2020 - 2 (Kaufmann et al., 2020)	56	15.30	23.10	53.40	MNI	AN<HC	26	30	adults	short-rec	ct	Freesurfer
Kohmura 2017 (Kohmura et al., 2017)	52	48	-36	12	MNI	AN<HC	23	29	adults	acute	gmv	VBM 8 - SPM8
	52	51	-36	-11	MNI	AN<HC	23	29	adults	acute	gmv	VBM 8 - SPM8
	52	54	-40	9	MNI	AN<HC	23	29	adults	acute	gmv	VBM 8 - SPM8
	52	3	14	37	MNI	AN<HC	23	29	adults	acute	gmv	VBM 8 - SPM8
	52	0	-16	45	MNI	AN<HC	23	29	adults	acute	gmv	VBM 8 - SPM8
	52	0	53	10	MNI	AN<HC	23	29	adults	acute	gmv	VBM 8 - SPM8
	52	-39	-64	43	MNI	AN<HC	23	29	adults	acute	gmv	VBM 8 - SPM8
	52	-29	-79	39	MNI	AN<HC	23	29	adults	acute	gmv	VBM 8 - SPM8
	52	-45	-70	33	MNI	AN<HC	23	29	adults	acute	gmv	VBM 8 - SPM8
	52	-57	-19	-9	MNI	AN<HC	23	29	adults	acute	gmv	VBM 8 - SPM8
	52	-59	-12	-24	MNI	AN<HC	23	29	adults	acute	gmv	VBM 8 - SPM8
	52	-56	3	-23	MNI	AN<HC	23	29	adults	acute	gmv	VBM 8 - SPM8
	52	-26	44	19	MNI	AN<HC	23	29	adults	acute	gmv	VBM 8 - SPM8
	52	-56	-3	3	MNI	AN<HC	23	29	adults	acute	gmv	VBM 8 - SPM8
	52	-54	6	18	MNI	AN<HC	23	29	adults	acute	gmv	VBM 8 - SPM8
	52	-29	-31	48	MNI	AN<HC	23	29	adults	acute	gmv	VBM 8 - SPM8
Lenhart 2022 (Lenhart et al., 2022)	40	-62	-8	-11	MNI	AN<HC	22	18	adolescents	acute	gmv	CAT12 - SPM12
	40	-54	-8	-17	MNI	AN<HC	22	18	adolescents	acute	gmv	CAT12 - SPM12
	40	-59	-36	48	MNI	AN<HC	22	18	adolescents	acute	gmv	CAT12 - SPM12
	40	-35	38	-14	MNI	AN<HC	22	18	adolescents	acute	gmv	CAT12 - SPM12
	40	-27	35	-12	MNI	AN<HC	22	18	adolescents	acute	gmv	CAT12 - SPM12

	40	-21	-65	-15	MNI	AN<HC	22	18	adolescents	acute	gmv	CAT12 - SPM12
	40	-6	-62	17	MNI	AN<HC	22	18	adolescents	acute	gmv	CAT12 - SPM12
	40	-30	-81	32	MNI	AN<HC	22	18	adolescents	acute	gmv	CAT12 - SPM12
	40	-3	-6	8	MNI	AN<HC	22	18	adolescents	acute	gmv	CAT12 - SPM12
	40	8	-20	17	MNI	AN<HC	22	18	adolescents	acute	gmv	CAT12 - SPM12
	40	-24	11	-26	MNI	AN<HC	22	18	adolescents	acute	gmv	CAT12 - SPM12
	40	-30	6	-33	MNI	AN<HC	22	18	adolescents	acute	gmv	CAT12 - SPM12
	40	-33	-74	-48	MNI	AN<HC	22	18	adolescents	acute	gmv	CAT12 - SPM12
	40	38	-18	15	MNI	AN<HC	22	18	adolescents	acute	gmv	CAT12 - SPM12
	40	60	-14	-9	MNI	AN<HC	22	18	adolescents	acute	gmv	CAT12 - SPM12
	40	56	-48	45	MNI	AN<HC	22	18	adolescents	acute	gmv	CAT12 - SPM12
	40	57	-6	38	MNI	AN<HC	22	18	adolescents	acute	gmv	CAT12 - SPM12
	40	44	-15	39	MNI	AN<HC	22	18	adolescents	acute	gmv	CAT12 - SPM12
	40	56	11	26	MNI	AN<HC	22	18	adolescents	acute	gmv	CAT12 - SPM12
	40	57	-14	-26	MNI	AN<HC	22	18	adolescents	acute	gmv	CAT12 - SPM12
	40	26	-11	-18	MNI	AN<HC	22	18	adolescents	acute	gmv	CAT12 - SPM12
	40	20	-2	-15	MNI	AN<HC	22	18	adolescents	acute	gmv	CAT12 - SPM12
	40	20	-48	-14	MNI	AN<HC	22	18	adolescents	acute	gmv	CAT12 - SPM12
	40	36	-45	-45	MNI	AN<HC	22	18	adolescents	acute	gmv	CAT12 - SPM12
Leppanen 2019 (Leppanen et al., 2019)	100	-22.9	-64.2	29.7	MNI	AN<HC	46	54	adults	acute	ct	Freesurfer
	100	-14.4	-41	67.1	MNI	AN<HC	46	54	adults	acute	ct	Freesurfer
	100	-5.6	34.4	2.6	MNI	AN<HC	46	54	adults	acute	ct	Freesurfer
	100	11.7	-52.2	64.9	MNI	AN<HC	46	54	adults	acute	ct	Freesurfer
	100	21.3	-88.4	18.2	MNI	AN<HC	46	54	adults	acute	ct	Freesurfer
	100	18.1	-70.8	35.7	MNI	AN<HC	46	54	adults	acute	ct	Freesurfer
Martin Monzon 2017 (Martin Monzon et al., 2017)	20	-18.5	1.5	-24	MNI	AN<HC	10	10	adolescents	acute	gmv	SPM12
	20	-13.5	9	19.5	MNI	AN<HC	10	10	adolescents	acute	gmv	SPM12
	20	-3	36	0	MNI	AN<HC	10	10	adolescents	acute	gmv	SPM12
	20	-10.39	-15.85	38.24	MNI	AN<HC	10	10	adolescents	acute	gmv	SPM12
	20	0	-46.83	17.57	MNI	AN<HC	10	10	adolescents	acute	gmv	SPM12
	20	-39	-1.5	9	MNI	AN<HC	10	10	adolescents	acute	gmv	SPM12
	20	45	1.5	7.5	MNI	AN<HC	10	10	adolescents	acute	gmv	SPM12
	20	-30	-40.5	1.5	MNI	AN<HC	10	10	adolescents	acute	gmv	SPM12
	20	-9	-94.5	-9	MNI	AN<HC	10	10	adolescents	acute	gmv	SPM12
	20	19.50	-99	-9	MNI	AN<HC	10	10	adolescents	acute	gmv	SPM12
	20	-36	36	-12	MNI	AN<HC	10	10	adolescents	acute	gmv	SPM12
	20	36	39	-10.5	MNI	AN<HC	10	10	adolescents	acute	gmv	SPM12
	20	-26.19	-76.87	31.09	MNI	AN<HC	10	10	adolescents	acute	gmv	SPM12
	20	29.53	-76.87	31.09	MNI	AN<HC	10	10	adolescents	acute	gmv	SPM12

	20	-4.5	-57	51	MNI	AN<HC	10	10	adolescents	acute	gmv	SPM12
	20	7.5	-51	64.5	MNI	AN<HC	10	10	adolescents	acute	gmv	SPM12
	20	-45.63	34.71	22.62	MNI	AN<HC	10	10	adolescents	acute	gmv	SPM12
	20	40.82	48.56	21.08	MNI	AN<HC	10	10	adolescents	acute	gmv	SPM12
	20	-15	57	-12	MNI	AN<HC	10	10	adolescents	acute	gmv	SPM12
	20	16.5	58.5	-10.5	MNI	AN<HC	10	10	adolescents	acute	gmv	SPM12
	20	-4.5	-11.54	14.98	MNI	AN<HC	10	10	adolescents	acute	gmv	SPM12
	20	7.5	-3	6	MNI	AN<HC	10	10	adolescents	acute	gmv	SPM12
	20	25.5	1.5	-21	MNI	AN<HC	10	10	adolescents	acute	gmv	SPM12
	20	15	9	21	MNI	AN<HC	10	10	adolescents	acute	gmv	SPM12
	20	30	-37.5	6	MNI	AN<HC	10	10	adolescents	acute	gmv	SPM12
Mishima 2021 (Mishima et al., 2021)	70	3	-69	46	MNI	AN<HC	35	35	adults	acute	gmv	CAT12
	70	-38	-54	51	MNI	AN<HC	35	35	adults	acute	gmv	CAT12
	70	21	-69	-26	MNI	AN<HC	35	35	adults	acute	gmv	CAT12
	70	44	18	32	MNI	AN<HC	35	35	adults	acute	gmv	CAT12
	70	-26	-6	62	MNI	AN<HC	35	35	adults	acute	gmv	CAT12
	70	64	-16	-8	MNI	AN<HC	35	35	adults	acute	gmv	CAT12
	70	24	-26	-15	MNI	AN<HC	35	35	adults	acute	gmv	CAT12
	70	-52	0	-27	MNI	AN<HC	35	35	adults	acute	gmv	CAT12
	70	-16	-68	57	MNI	AN<HC	35	35	adults	acute	ct	CAT12
	70	66	-39	-4	MNI	AN<HC	35	35	adults	acute	ct	CAT12
	70	49	-24	57	MNI	AN<HC	35	35	adults	acute	ct	CAT12
	70	6	-69	49	MNI	AN<HC	35	35	adults	acute	ct	CAT12
	70	60	-28	44	MNI	AN<HC	35	35	adults	acute	ct	CAT12
	70	-22	8	61	MNI	AN<HC	35	35	adults	acute	ct	CAT12
	70	55	-52	40	MNI	AN<HC	35	35	adults	acute	ct	CAT12
	70	20	34	46	MNI	AN<HC	35	35	adults	acute	ct	CAT12
	70	-7	-50	53	MNI	AN<HC	35	35	adults	acute	ct	CAT12
	70	-8	-86	35	MNI	AN<HC	35	35	adults	acute	ct	CAT12
	70	-7	-68	10	MNI	AN<HC	35	35	adults	acute	ct	CAT12
	70	34	-49	-8	MNI	AN<HC	35	35	adults	acute	ct	CAT12
Mühlau 2007 (Mühlau et al., 2007)	59	0	0	0	MNI	AN<HC	22	37	adults	longer-rec	gmv	SPM2
	59	-11	24	23	MNI	AN<HC	22	37	adults	longer-rec	gmv	SPM2
	59	-31	47	28	MNI	AN<HC	22	37	adults	longer-rec	gmv	SPM2
	59	-64	-47	-1	MNI	AN<HC	22	37	adults	longer-rec	gmv	SPM2
	59	49	12	-1	MNI	AN<HC	22	37	adults	longer-rec	gmv	SPM2
	59	50	3	-2	MNI	AN<HC	22	37	adults	longer-rec	gmv	SPM2
	59	32	-56	54	MNI	AN<HC	22	37	adults	longer-rec	gmv	SPM2
	59	22	-59	55	MNI	AN<HC	22	37	adults	longer-rec	gmv	SPM2

	59	12	-27	41	MNI	AN<HC	22	37	adults	longer-rec	gmv	SPM2
	59	-50	-64	-9	MNI	AN<HC	22	37	adults	longer-rec	gmv	SPM2
	59	-25	55	-3	MNI	AN<HC	22	37	adults	longer-rec	gmv	SPM2
	59	-23	-99	1	MNI	AN<HC	22	37	adults	longer-rec	gmv	SPM2
	59	-50	16	3	MNI	AN<HC	22	37	adults	longer-rec	gmv	SPM2
	59	5	55	-5	MNI	AN<HC	22	37	adults	longer-rec	gmv	SPM2
	59	7	37	26	MNI	AN<HC	22	37	adults	longer-rec	gmv	SPM2
	59	-5	35	-10	MNI	AN<HC	22	37	adults	longer-rec	gmv	SPM2
	59	9	42	8	MNI	AN<HC	22	37	adults	longer-rec	gmv	SPM2
	59	11	6	39	MNI	AN<HC	22	37	adults	longer-rec	gmv	SPM2
Nickel 2018 (Nickel et al., 2018)	75	38	-22	-4	MNI	AN<HC	34	41	adults	acute	gmv	CAT12 - SPM12
	75	-24	40	28	MNI	AN<HC	34	41	adults	acute	gmv	CAT12 - SPM12
	75	56	24	4	MNI	AN<HC	34	41	adults	acute	gmv	CAT12 - SPM12
	75	-16	-10	65	MNI	AN<HC	34	41	adults	acute	ct	CAT12 - SPM12
	75	-22	24	37	MNI	AN<HC	34	41	adults	acute	ct	CAT12 - SPM12
	75	17	17	60	MNI	AN<HC	34	41	adults	acute	ct	CAT12 - SPM12
	75	21	-6	60	MNI	AN<HC	34	41	adults	acute	ct	CAT12 - SPM12
	75	-38	5	39	MNI	AN<HC	34	41	adults	acute	ct	CAT12 - SPM12
	75	30	8	53	MNI	AN<HC	34	41	adults	acute	ct	CAT12 - SPM12
	75	-6	17	64	MNI	AN<HC	34	41	adults	acute	ct	CAT12 - SPM12
	75	-54	23	17	MNI	AN<HC	34	41	adults	acute	ct	CAT12 - SPM12
	75	-40	-62	20	MNI	AN<HC	34	41	adults	acute	ct	CAT12 - SPM12
	75	-15	-40	43	MNI	AN<HC	34	41	adults	acute	ct	CAT12 - SPM12
	75	-12	-52	38	MNI	AN<HC	34	41	adults	acute	ct	CAT12 - SPM12
	75	13	-30	46	MNI	AN<HC	34	41	adults	acute	ct	CAT12 - SPM12
	75	17	-70	46	MNI	AN<HC	34	41	adults	acute	ct	CAT12 - SPM12
	75	8	-55	44	MNI	AN<HC	34	41	adults	acute	ct	CAT12 - SPM12
	75	38	23	9	MNI	AN<HC	34	41	adults	acute	ct	CAT12 - SPM12
Oliva 2020 (Oliva et al., 2020)	30	-42	36	-17	MNI	AN<HC	15	15	adults	longer-rec	gmv	CAT12 - SPM12
Phillipou 2018 (Phillipou et al., 2018)	53	-54	0	-20	MNI	AN<HC	26	27	adults	acute	gmv	SPM12
	53	57	-21	-14	MNI	AN<HC	26	27	adults	acute	gmv	SPM12
	53	-46	6	28	MNI	AN<HC	26	27	adults	acute	gmv	SPM12
	53	-14	-40	48	MNI	AN<HC	26	27	adults	acute	gmv	SPM12
	53	24	-8	52	MNI	AN<HC	26	27	adults	acute	gmv	SPM12
	53	23	-42	51	MNI	AN<HC	26	27	adults	acute	gmv	SPM12
	53	-47	-45	42	MNI	AN<HC	26	27	adults	acute	gmv	SPM12
	53	-5	-24	-9	MNI	AN<HC	26	27	adults	acute	gmv	SPM12
	53	-3	11	-15	MNI	AN<HC	26	27	adults	acute	gmv	SPM12
	53	21	-63	-41	MNI	AN<HC	26	27	adults	acute	gmv	SPM12

Seitz 2015 (Seitz et al., 2015)	106	47	-59.6	44.2	TAL	AN<HC	56	50	adolescents	acute	gmv	Freesurfer
	106	35.2	-14.8	19.3	TAL	AN<HC	56	50	adolescents	acute	gmv	Freesurfer
	106	5.2	-33.7	40.9	TAL	AN<HC	56	50	adolescents	acute	gmv	Freesurfer
	106	53.2	-28	0.6	TAL	AN<HC	56	50	adolescents	acute	gmv	Freesurfer
	106	39.2	9	54.7	TAL	AN<HC	56	50	adolescents	acute	gmv	Freesurfer
	106	21.1	-47.9	-5.2	TAL	AN<HC	56	50	adolescents	acute	gmv	Freesurfer
	106	54.1	-59.2	4.9	TAL	AN<HC	56	50	adolescents	acute	gmv	Freesurfer
	106	31.2	47.2	7.4	TAL	AN<HC	56	50	adolescents	acute	gmv	Freesurfer
	106	44.1	-23.8	60.2	TAL	AN<HC	56	50	adolescents	acute	gmv	Freesurfer
	106	55.1	-36.2	17.6	TAL	AN<HC	56	50	adolescents	acute	gmv	Freesurfer
	106	32.4	33.3	-8.4	TAL	AN<HC	56	50	adolescents	acute	gmv	Freesurfer
	106	48.9	8.2	-23.7	TAL	AN<HC	56	50	adolescents	acute	gmv	Freesurfer
	106	10.9	23.4	56.1	TAL	AN<HC	56	50	adolescents	acute	gmv	Freesurfer
	106	25.2	-97.5	0	TAL	AN<HC	56	50	adolescents	acute	gmv	Freesurfer
	106	23.3	43.9	23.4	TAL	AN<HC	56	50	adolescents	acute	gmv	Freesurfer
	106	19.2	-81.5	38,2	TAL	AN<HC	56	50	adolescents	acute	gmv	Freesurfer
	106	64.1	-34.7	-13.3	TAL	AN<HC	56	50	adolescents	acute	gmv	Freesurfer
	106	53.2	-21.4	17.8	TAL	AN<HC	56	50	adolescents	acute	gmv	Freesurfer
	106	44	-65.9	25.9	TAL	AN<HC	56	50	adolescents	acute	gmv	Freesurfer
	106	33.6	7.4	26.4	TAL	AN<HC	56	50	adolescents	acute	gmv	Freesurfer
	106	55	-2.5	22.8	TAL	AN<HC	56	50	adolescents	acute	gmv	Freesurfer
	106	11.4	32.7	-18.5	TAL	AN<HC	56	50	adolescents	acute	gmv	Freesurfer
	106	17.6	38.3	-18.2	TAL	AN<HC	56	50	adolescents	acute	gmv	Freesurfer
	106	17	-92.8	1.6	TAL	AN<HC	56	50	adolescents	acute	gmv	Freesurfer
	106	56	-29.3	43.7	TAL	AN<HC	56	50	adolescents	acute	gmv	Freesurfer
	106	47.3	-71.4	9.2	TAL	AN<HC	56	50	adolescents	acute	gmv	Freesurfer
	106	51.9	-45.1	31.4	TAL	AN<HC	56	50	adolescents	acute	gmv	Freesurfer
	106	23	-60.3	39.1	TAL	AN<HC	56	50	adolescents	acute	gmv	Freesurfer
	106	38.5	-13.1	56.9	TAL	AN<HC	56	50	adolescents	acute	gmv	Freesurfer
	106	22.4	-6.9	49.4	TAL	AN<HC	56	50	adolescents	acute	gmv	Freesurfer
	106	23.2	-25.9	-17.9	TAL	AN<HC	56	50	adolescents	acute	gmv	Freesurfer
	106	34,6	-67.4	40	TAL	AN<HC	56	50	adolescents	acute	gmv	Freesurfer
	106	17.4	-95.2	14.2	TAL	AN<HC	56	50	adolescents	acute	gmv	Freesurfer
	106	-9.8	-34.8	53.4	TAL	AN<HC	56	50	adolescents	acute	gmv	Freesurfer
	106	-13.3	-76.5	45.6	TAL	AN<HC	56	50	adolescents	acute	gmv	Freesurfer
	106	-59.5	-54.7	3.1	TAL	AN<HC	56	50	adolescents	acute	gmv	Freesurfer
	106	-50.4	-20.5	55.1	TAL	AN<HC	56	50	adolescents	acute	gmv	Freesurfer
	106	-32.8	-33.5	-24	TAL	AN<HC	56	50	adolescents	acute	gmv	Freesurfer
	106	-36.2	-16.2	20.3	TAL	AN<HC	56	50	adolescents	acute	gmv	Freesurfer

	106	-51.4	6.3	-14	TAL	AN<HC	56	50	adolescents	acute	gmv	Freesurfer
	106	-55.9	-41.9	42	TAL	AN<HC	56	50	adolescents	acute	gmv	Freesurfer
	106	-28.4	-93.3	-2.7	TAL	AN<HC	56	50	adolescents	acute	gmv	Freesurfer
	106	-49.6	-7.7	36.7	TAL	AN<HC	56	50	adolescents	acute	gmv	Freesurfer
	106	-56.1	-12.5	1.7	TAL	AN<HC	56	50	adolescents	acute	gmv	Freesurfer
	106	-30	-71.4	-8.6	TAL	AN<HC	56	50	adolescents	acute	gmv	Freesurfer
	106	-15.4	44.7	-18.3	TAL	AN<HC	56	50	adolescents	acute	gmv	Freesurfer
	106	-39.2	5.5	49.8	TAL	AN<HC	56	50	adolescents	acute	gmv	Freesurfer
	106	-52.1	-36	1.4	TAL	AN<HC	56	50	adolescents	acute	gmv	Freesurfer
	106	-4.4	-41.9	28.3	TAL	AN<HC	56	50	adolescents	acute	gmv	Freesurfer
	106	-33.6	-65.2	41.3	TAL	AN<HC	56	50	adolescents	acute	gmv	Freesurfer
	106	-36.4	-17.4	9.5	TAL	AN<HC	56	50	adolescents	acute	gmv	Freesurfer
	106	-31.9	-24.6	51.7	TAL	AN<HC	56	50	adolescents	acute	gmv	Freesurfer
	106	-36	-51.9	59.8	TAL	AN<HC	56	50	adolescents	acute	gmv	Freesurfer
	106	-14.2	-87.8	24.5	TAL	AN<HC	56	50	adolescents	acute	gmv	Freesurfer
	106	-7.2	29.8	-17.1	TAL	AN<HC	56	50	adolescents	acute	gmv	Freesurfer
	106	-41.1	-52.2	-18.7	TAL	AN<HC	56	50	adolescents	acute	gmv	Freesurfer
	106	-43.5	-57.1	38.6	TAL	AN<HC	56	50	adolescents	acute	gmv	Freesurfer
	106	-59.7	-49.5	27.6	TAL	AN<HC	56	50	adolescents	acute	gmv	Freesurfer
	106	-48.2	-30.7	3	TAL	AN<HC	56	50	adolescents	acute	gmv	Freesurfer
	106	-23.6	-86	12.9	TAL	AN<HC	56	50	adolescents	acute	gmv	Freesurfer
	106	-38.8	-63	29	TAL	AN<HC	56	50	adolescents	acute	gmv	Freesurfer
	106	-36.2	-14.5	-29.5	TAL	AN<HC	56	50	adolescents	acute	gmv	Freesurfer
	106	-43.2	7	-35	TAL	AN<HC	56	50	adolescents	acute	gmv	Freesurfer
	106	-13.7	-101.7	3.9	TAL	AN<HC	56	50	adolescents	acute	gmv	Freesurfer
	106	-30.4	-56.8	36.9	TAL	AN<HC	56	50	adolescents	acute	gmv	Freesurfer
Suchan 2010 (Suchan et al., 2010)	30	-48	-66	9	MNI	AN<HC	15	15	adults	acute	gmv	SPM5
	30	-56	-50	15	MNI	AN<HC	15	15	adults	acute	gmv	SPM5
Tose 2024 (Tose et al., 2024)	205	−38	−50	−30	MNI	AN<HC	103	102	adults	acute	gmv	CAT12 - SPM12
	205	−2	−23	47	MNI	AN<HC	103	102	adults	acute	gmv	CAT12 - SPM12
	205	−3	39	−22	MNI	AN<HC	103	102	adults	acute	gmv	CAT12 - SPM12
	205	−50	−60	28	MNI	AN<HC	103	102	adults	acute	gmv	CAT12 - SPM12
	205	46	−4	3	MNI	AN<HC	103	102	adults	acute	gmv	CAT12 - SPM12
	205	−62	−10	−10	MNI	AN<HC	103	102	adults	acute	gmv	CAT12 - SPM12
	205	−51	4	39	MNI	AN<HC	103	102	adults	acute	gmv	CAT12 - SPM12
	205	−36	−24	60	MNI	AN<HC	103	102	adults	acute	gmv	CAT12 - SPM12
	205	0	−14	8	MNI	AN<HC	103	102	adults	acute	gmv	CAT12 - SPM12
	205	−32	−40	45	MNI	AN<HC	103	102	adults	acute	gmv	CAT12 - SPM12
Van Opstal 2015 (van Opstal et al., 2015)	20	8	22	30	MNI	AN<HC	10	10	adults	acute	gmv	FSL-VBM

	20	-14	-16	48	MNI	AN<HC	10	10	adults	acute	gmv	FSL-VBM
Yu 2024 (Yu et al., 2024)	120	0	-12	57	MNI	AN<HC	64	56	adults	acute	gmv	CAT12 - SPM12
	120	3	-3	51	MNI	AN<HC	64	56	adults	acute	gmv	CAT12 - SPM12
	120	-2	-6	72	MNI	AN<HC	64	56	adults	acute	gmv	CAT12 - SPM12
	120	-6	-4	4	MNI	AN<HC	64	56	adults	acute	gmv	CAT12 - SPM12
	120	-2	-16	14	MNI	AN<HC	64	56	adults	acute	gmv	CAT12 - SPM12
	120	-15	-10	15	MNI	AN<HC	64	56	adults	acute	gmv	CAT12 - SPM12
	120	-4	64	8	MNI	AN<HC	64	56	adults	acute	gmv	CAT12 - SPM12
	120	-4	45	28	MNI	AN<HC	64	56	adults	acute	gmv	CAT12 - SPM12
	120	-10	40	15	MNI	AN<HC	64	56	adults	acute	gmv	CAT12 - SPM12
	120	-51	44	-10	MNI	AN<HC	64	56	adults	acute	gmv	CAT12 - SPM12
	120	-54	32	-10	MNI	AN<HC	64	56	adults	acute	gmv	CAT12 - SPM12
	120	-46	40	3	MNI	AN<HC	64	56	adults	acute	gmv	CAT12 - SPM12
	120	48	45	9	MNI	AN<HC	64	56	adults	acute	gmv	CAT12 - SPM12
	120	51	30	-2	MNI	AN<HC	64	56	adults	acute	gmv	CAT12 - SPM12
	120	-6	16	-15	MNI	AN<HC	64	56	adults	acute	gmv	CAT12 - SPM12
	120	-9	38	-18	MNI	AN<HC	64	56	adults	acute	gmv	CAT12 - SPM12
	120	-16	20	-21	MNI	AN<HC	64	56	adults	acute	gmv	CAT12 - SPM12
	120	-36	50	19	MNI	AN<HC	64	56	adults	acute	ct	CAT12 - SPM12
	120	-43	39	21	MNI	AN<HC	64	56	adults	acute	ct	CAT12 - SPM12
	120	-46	22	20	MNI	AN<HC	64	56	adults	acute	ct	CAT12 - SPM12
	120	-43	-51	15	MNI	AN<HC	64	56	adults	acute	ct	CAT12 - SPM12
	120	-38	-58	20	MNI	AN<HC	64	56	adults	acute	ct	CAT12 - SPM12
	120	-51	-46	3	MNI	AN<HC	64	56	adults	acute	ct	CAT12 - SPM12
	120	-10	-23	48	MNI	AN<HC	64	56	adults	acute	ct	CAT12 - SPM12
	120	46	-33	44	MNI	AN<HC	64	56	adults	acute	ct	CAT12 - SPM12
	120	44	-26	41	MNI	AN<HC	64	56	adults	acute	ct	CAT12 - SPM12
	120	10	-55	39	MNI	AN<HC	64	56	adults	acute	ct	CAT12 - SPM12

Abbreviations: *AN, A*norexia nervosa; *ct*, cortical thickness; *gmv*, gray matter volume; *HC,* Healthy controls; *MNI,* Montreal Neurological Institutes; *sd,* Standard deviation; *TAL,* Talairach.

3 (B) - Study	N patients / HCs	Age group	AN status	Measure	Age (SD) patients / HCs in years	BMI (SD) patients / HCs	Mean illness duration (SD) patients	NOS total score
Amianto 2013 (Amianto et al., 2013)	17 / 14	adults	acute	vol	20.0 (4.0) / 24.0 (3.0)	16.00 (1.00) / 21.00 (2.00)	13.0 (8.0) months	9
Bär 2015 (Bär et al., 2015)	26 / 26	adults	acute	ct, vol	22.96 (4.97) / 24.00 (1.92)	16.97 (1.46) / 21.72 (1.5)	22.4 (14.80) months	8
Boghi 2011 (Boghi et al., 2011)	21 / 27	adults	acute	vol	29.0 (10.0) / 30.8 (8.7)	15.5 (1.75) / 21.9 (1.5)	11.3 (12.10) years	8
Bomba 2015 (Bomba et al., 2015)	11 / 8	adolescents	acute	vol	13.63 (2.77) / 13.25 (2.43)	12.76 (0.84) / 19.87 (1.45)	14.45 (10.92) months	6
Brooks 2011 (Brooks et al., 2011)	14 / 21	adults	acute	vol	26.0 (1.9) / 26.0 (2.1)	15.6 (0.4) / 21.4 (0.5)	9.2 (1.90) years	9
Cascino 2020 (Cascino et al., 2020)	22 / 35	adults	acute	ct	28.63 (9.76) / 26.77 (5.24)	16.37 (1.56) / 21.13 (1.96)	12.46 (8.63) - no unit reported	6
Castro-Fornieles 2009 - 1 (Castro-Fornieles et al., 2009)	12 / 9	adolescents	acute	vol	14.5 (1.5) / 14.6 (3.2)	14.8 (2) / NA (NA)	8.3 (3.1) months	6
Castro-Fornieles 2009 - 2 (Castro-Fornieles et al., 2009)	12 / 9	adolescents	short-rec	vol	14.5 (1.5) / 14.6 (3.2)	18.8 (0.4) / NA (NA)	NA	6
Castro-Fornieles 2021 (Castro-Fornieles et al., 2021)	15 / 28	adults	longer-rec	ct	35.6 (2.5) / 36.5 (3.0)	21.1 (1.9) / 22.2 (2.1)	NA	7
Collantoni 2024 (Collantoni et al., 2024)	28 / 52	adolescents	short-rec	ct	15.54 (1.91) / 15.74 (1.65)	BMI-SDS: -1.01 (0.49) / 0.16 (0.87)	16.24 (9.88) months	7
D'Agata 2015 (D'Agata et al., 2015)	21 / 17	adults	acute	vol	21.0 (5.0) / 23.0 (4.0)	16.1 (0.9) / 21.5 (2.3)	less than 2 years	9
De la Cruz 2021 (de la Cruz et al., 2021)	22 / 26	adults	acute	ct	23.8 (7.2) / 25.2 (6.6)	15.1 (1.4) / 24 (3.2)	NA	7
Favaro 2015 (Favaro et al., 2015)	38 / 38	adults	acute	ct	26.1 (7.2) / 25.3 (6.3)	15.8 (1.8) / 21.7 (2.9)	78.6 (81.30) months	8
Fonville 2014 (Fonville et al., 2014)	31 / 31	adults	acute	vol	23.0 (10.0) / 25.0 (4.0)	15.8 (1.4) / 21.8 (1.8)	7.0 (10.0) years	9
Frank 2013a - 1 (Frank et al., 2013a)	19 / 24	adults	acute	vol	23.1 (5.8) / 27.4 (6.3)	16 (1.1) / 21.6 (1.3)	NA	8
Frank 2013a - 2 (Frank et al., 2013a)	24 / 24	adults	short-rec	vol	30.3 (8.1) / 27.4 (6.3)	20.8 (2.4) / 21.6 (1.3)	NA	8

Frank 2013b (Frank et al., 2013b)	19 / 22	adolescents	acute	vol	15.4 (1.4) / 14.8 (1.8)	16.2 (1.1) / 21.3 (1.9)	NA	8
Friederich 2012 - 1 (Friederich et al., 2012)	12 / 14	adults	acute	vol	24.3 (6.2) / 25.6 (3.7)	15.9 (1.6) / 21.1 (1.5)	6.3 (4.40) years	9
Friederich 2012 - 2 (Friederich et al., 2012)	13 / 14	adults	longer-rec	vol	25.0 (4.8) / 25.6 (3.7)	19.5 (1.4) / 21.1 (1.5)	5.7 (3.60) years	9
Fujisawa 2015 (Fujisawa et al., 2015)	20 / 14	adolescents	acute	vol	14.15 (1.81) / 14.93 (1.59)	14.35 (2.084) / NA (NA)	23.55 (17.02) months	7
Gaudio 2011 (Gaudio et al., 2011)	16 / 16	adolescents	acute	vol	15.2 (1.7) / 15.1 (1.5)	14.2 (1.4) / 20.2 (1.6)	5.3 (3.2) months	8
Gaudio 2017 (Gaudio et al., 2017)	14 / 15	adolescents	acute	vol	15.7 (1.6) / 16.3 (1.5)	16.2 (1.2) / 21.1 (1.9)	4.9 (1.8) months	8
Joos 2010 (Joos et al., 2010)	12 / 18	adults	acute	vol	25.0 (4.8) / 26.9 (5.7)	16 (1.2) / 21.2 (2)	4.7 (3.60) years	8
Kaufmann 2020 - 1 (Kaufmann et al., 2020)	24 / 30	adults	acute	ct	22.25 (4.08) / 24.15 (3.42)	14.25 (1.08) / 20.81 (1.77)	5.59 (4.30) years	8
Kaufmann 2020 - 2 (Kaufmann et al., 2020)	26 / 30	adults	short-rec	ct	22.25 (4.08) / 24.15 (3.42)	18.41 (0.45) / 20.38 (1.56)	5.59 (4.30) years	8
Kohmura 2017 (Kohmura et al., 2017)	23 / 29	adults	acute	vol	28.5 (6.7) / 28.2 (7.0)	13.2 (1.5) / 21.5 (3.3)	10.5 (6.20) years	8
Lenhart 2022 (Lenhart et al., 2022)	22 / 18	adolescents	acute	vol	15.8 (1.2) / 17.7 (0.7)	15.4 (1.4) / 21.2 (1.0)	9.4 (6.8) months	8
Leppanen 2019 (Leppanen et al., 2019)	46 / 54	adults	acute	ct	27.51 (9.24) / 26.35 (4.47)	15.73 (1.41) / 21.49 (1.97)	11.39 (9.22) years	7
Martin Monzon 2017 (Martin Monzon et al., 2017)	10 / 10	adolescents	acute	vol	16.10 (0.33) / NA (NA)	16.31 (0.33) / 21.06 (0.85)	< 3 years	8
Mishima 2021 (Mishima et al., 2021)	35 / 35	adults	acute	vol	36.3 (10.0) / 36.0 (9.6)	14.2 (2.5) / 21 (2.9)	15.7 (9.00) years	8
Mühlau 2007 (Mühlau et al., 2007)	22 / 37	adults	longer-rec	vol	23.7 (6.0) / 24.7 (4.3)	19.7 (1.6) / 20.7 (1.8)	5.2 (5.10) years	7
Nickel 2018 (Nickel et al., 2018)	34 / 41	adults	acute	vol, ct	23.8 (4.3) / 23.6 (3.8)	16.1 (1.4) / 22.3 (2.4)	6.6 (3.70) years	7
Oliva 2020 (Oliva et al., 2020)	15 / 15	adults	longer-rec	vol	25.87 (6.15) / 25.2 (1.01)	20.1 (2.04) / 21.32 (2.45)	38.4 (38.00) months	7

Phillipou 2018<br>(Phillipou et al., 2018)	26 / 27	adults	acute	vol	22.81 (6.67) /<br>22.46 (3.16)	16.63 (1.19) /<br>22.6 (3.53)	6.42 (7.43) years	9
Seitz 2015<br>(Seitz et al., 2015)	56 / 50	adolescents	acute	vol	15.47 (1.73) /<br>15.75 (1.71)	15.13 (1.37) /<br>21.37 (3.32)	11.43 (8.31) months	7
Suchan 2010<br>(Suchan et al., 2010)	15 / 15	adults	acute	vol	26.8 (8.4) /<br>29.5 (8.2)	16.00 (1.30) /<br>22.00 (2.10)	5.5 (5.00) years	6
Tose 2024<br>(Tose et al., 2024)	103 / 102	adults	acute	vol	33.11 (12.17) /<br>31.31 (11.04)	14.74 (2.25) /<br>20.84 (2.59)	NA	7
Van Opstal 2015<br>(van Opstal et al., 2015)	10 / 10	adults	acute	vol	22.1 (3.3) /<br>20.8 (0.52)	15.6 (1.02) /<br>20.3 (1.5)	3.54 (2.30) years	6
Yu 2024<br>(Yu et al., 2024)	64 / 56	adults	acute	vol, ct	21.78 y (2.1) /<br>22.72 y (0.62)	16.62 (1.80) /<br>24.91 (4.72)	NA	8

Abbreviations: *AN, A*norexia nervosa; *HC,* Healthy controls; *SD,* Standard deviation; *ct,* Cortical thickness; *vol,* Volume; *NOS*, Newcastle-Ottawa Scale.

Supplementary Table 4. Pooled age and BMI for participants (patients and HCs) included in the different meta-analyses separated by age group. As only few studies on recovered patients were included in the ALE meta-analysis "GM_loss_acute_and_recovered" and no ALE sub-analysis in only recovered patients could be calculated due to the low number of studies, only scores for the main analysis "GM_loss_acute" are shown here.

		**Global brain volume meta-analysis**						**ALE meta-analysis**	
		AN_{acute}		$AN_{short\text{-}rec}$		$AN_{longer\text{-}rec}$		GM_loss_acute	
**Group**		Pooled age	Pooled BMI	Pooled age	Pooled BMI	Pooled age	Pooled BMI	Pooled age	Pooled BMI
**Patients**	**Adults**	24.16	15.71	26.54	20.33	25.15	20.44	26.45	15.57
	**Adolescents**	15.58	15.13	15.74	19.18	20.71	20.62	15.21	15.09
**HCs**	**Adults**	24.75	21.92	25.39	22.11	24.60	21.55	26.77	21.81
	**Adolescents**	15.93	20.97	15.97	21.70	20.49	21.50	15.55	21.06

Abbreviations: *GM*, Gray matter; *HCs,* Healthy controls; *short-rec,* Short-term weight recovered; *longer-rec,* Longer-term recovered (>1.5 years).

Supplementary Table 5. Average brain volume changes in patients with AN at different stages of disease compared to HCs as revealed by the global brain volume meta-analysis.

	**AN_{acute}**			**$AN_{short-rec}$**			**$AN_{longer-rec}$**		
	All	Adults	Adolescents	All	Adults	Adolescents	All	Adults	Adolescents
**Gray matter**									
**N patients/ HCs**	1014/ 1044	707/ 742	307/ 302	197/ 192	115/ 123	82/ 69	232/ 304	177/ 250	55/ 54
**%Vol**	- 4.79 %	- 4.13 %	- 6.31%	- 2.86 %	- 1.43 %	- 5.43 %	- 1.98 %	- 1.48 %	- 3.63 %
p	< 0.001	< 0.001	< 0.001	0.07	0.41	0.10	0.002	0.03	0.02
**White matter**									
**N patients/ HCs**	999/ 1050	672/ 722	327/ 328	197/ 192	115/ 123	82/ 69	229/ 301	208/281	21/ 20
**%Vol**	- 2.48 %	-1.99 %	- 3.48 %	- 1.76 %	- 1.44 %	- 2.32 %	- 1.40 %	- 1.24 %	- 2.97 %
p	< 0.001	< 0.001	< 0.001	0.12	0.30	0.31	0.20	0.27	0.44
**Cerebrospinal fluid**									
**N patients/ HCs**	476/ 516	361/ 392	115/ 124	171/ 162	89/ 93	82/ 69	178/ 232	157/ 212	21/ 20
**%Vol**	+ 17.11 %	+ 17.22 %	+ 16.75 %	+ 7.99 %	+ 9.11 %	+ 6.78 %	+ 2.63 %	+ 3.17 %	- 1.42 %
p	< 0.001	< 0.001	0.009	0.03	0.29	0.06	0.22	0.16	0.68

Abbreviations: *HCs,* Healthy controls; *%Vol,* Volume change in percent compared to healthy controls; *short-rec,* Short-term weight recovered; *longer-rec* Longer-term recovered (>1.5 years).

Supplementary Table 6. Structural differences in gray matter volume and cortical thickness between all patients with AN (acutely ill and recovered) and healthy controls revealed by the directed sub-analysis "GM_loss_acute_and_recovered" reporting significant clusters (TFCE-corrected) of brain volume loss in AN including MNI coordinates, cluster size and experiments contributing to the cluster.

**Cluster**	**Region**	**MNI**			**Cluster size (voxels)**	**N (experiments contributing to cluster)**	**References (experiments contributing to cluster)**
		**x**	**y**	**z**			
**1**	Cingulate gyrus, posterior division<br>Precentral gyrus	10	-28	44	134	7	(Amianto et al., 2013; Bär et al., 2015; Friederich et al., 2012 - 1; Gaudio et al., 2017; Mühlau et al., 2007; Nickel et al., 2018; Seitz et al., 2015)
**2**	Precentral gyrus<br>Cingulate gyrus, posterior division	-6	-24	46	61	8	(Bär et al., 2015; Friederich et al., 2012 - 1; Gaudio et al., 2017; Kohmura et al., 2017; Martin Monzon et al., 2017; Seitz et al., 2015; van Opstal et al., 2015; Yu et al., 2024)
**3**	Precentral gyrus<br>Juxtapositional lobule cortex	-4<br>0	-16<br>-12	64<br>58	49	10	(Amianto et al., 2013; Bär et al., 2015; Boghi et al., 2011; Castro-Fornieles et al., 2009 - 2; D'Agata et al., 2015; Friederich et al., 2012 - 2; Gaudio et al., 2017; Kohmura et al., 2017; Nickel et al., 2018; Yu et al., 2024)
**4**	Precuneous cortex<br>Cingulate gyrus, posterior division	8	-54	42	37	8	(Bär et al., 2015; Brooks et al., 2011; Fonville et al., 2014; Gaudio et al., 2011; Martin Monzon et al., 2017; Mishima et al., 2021; Nickel et al., 2018; Yu et al., 2024)
**5**	Cingulate gyrus, anterior division<br>Cingulate gyrus, posterior division	2	-14	46	37	8	(Bär et al., 2015; Castro-Fornieles et al., 2009 - 2; Friederich et al., 2012 - 1; Gaudio et al., 2017; Kohmura et al., 2017; Martin Monzon et al., 2017; Yu et al., 2024; Yu et al., 2024)
**6**	Juxtapositional lobule cortex<br>Precentral gyrus	2	-2	54	30	6	(Bär et al., 2015; Castro-Fornieles et al., 2009 - 2; Friederich et al., 2012 - 1; Friederich et al., 2012 - 2; Gaudio et al., 2017; Yu et al., 2024)
**7**	Precuneous cortex	-2	-54	52	21	6	(Bär et al., 2015; Fonville et al., 2014; Gaudio et al., 2011; Martin Monzon et al., 2017; Mishima et al., 2021; Nickel et al., 2018)
**8**	Lateral occipital cortex, superior division<br>Cuneal cortex	-22	-66	32	10	7	(Bär et al., 2015; Collantoni et al., 2024; de la Cruz et al., 2021; Fonville et al., 2014; Joos et al., 2010; Leppanen et al., 2019; Martin Monzon et al., 2017)

Abbreviations: *AN*, Anorexia nervosa; *GM*, Gray matter; *HCs*, Healthy controls; *MNI*, Montreal Neurological Institute; *TFCE*, Threshold-free cluster enhancement.

Supplementary Table 7. Structural differences in gray matter volume and cortical thickness between adult patients with acute AN and healthy controls revealed by the directed sub-analysis "GM_loss_acute_adults" reporting significant clusters (TFCE-corrected) of brain volume loss in AN including MNI coordinates, cluster size and experiments contributing to the cluster.

Cluster	Region	MNI			Cluster size (voxels)	N (experiments contributing to cluster)	References (experiments contributing to cluster)
		x	y	z			
**1**	Precuneous cortex<br>Cingulate gyrus, posterior division	8<br>4	-54<br>-68	42<br>48	161	7	(Bär et al., 2015; Brooks et al., 2011; Fonville et al., 2014; Mishima et al., 2021; Mishima et al., 2021; Nickel et al., 2018; Yu et al., 2024)
**2**	Precuneous cortex<br>Lateral occipital cortex, superior division	-20	-64	32	69	6	(Bär et al., 2015; de la Cruz et al., 2021; Fonville et al., 2014; Joos et al., 2010; Leppanen et al., 2019; Nickel et al., 2018)
**3**	Lateral occipital cortex, superior division<br>Precuneous cortex	16<br>18	-72<br>-70	44<br>36	47	4	(de la Cruz et al., 2021; Leppanen et al., 2019; Mishima et al., 2021; Nickel et al., 2018)
**4**	Precentral gyrus<br>Cingulate gyrus, posterior division	12	-28	44	46	4	(Amianto et al., 2013; Bär et al., 2015; Friederich et al., 2012 - 1; Nickel et al., 2018)
**5**	Precentral gyrus<br>Juxtapositional lobule cortex	-4	-16	64	42	5	(Amianto et al., 2013; Boghi et al., 2011; D'Agata et al., 2015; Nickel et al., 2018; Yu et al., 2024)
**6**	Juxtapositional lobule cortex<br>Precentral gyrus	2	-2	54	20	3	(Bär et al., 2015; Friederich et al., 2012 - 1; Yu et al., 2024)
**7**	Cingulate gyrus, posterior division<br>Precuneous cortex	8	-42	40	17	4	(Bär et al., 2015; Brooks et al., 2011; Nickel et al., 2018; Yu et al., 2024)
**8**	Angular gyrus<br>Lateral occipital cortex, superior division	-38	-60	20	1	NA	NA
**9**	Precentral gyrus<br>Juxtapositional lobule cortex	-10	-22	48	1	NA	NA

Abbreviations: *AN*, Anorexia nervosa; *GM*, Gray matter; *HCs*, Healthy controls; *MNI*, Montreal Neurological Institute; *TFCE*, Threshold-free cluster enhancement.

Supplementary Table 8. Functional decoding results for forward likelihood.

	likelihoodForward	pForward	qForward
**term**			
**flexibility**	2.299564	0.012842	0.278889
**reinforcement**	2.024184	0.019921	0.278889
**social interaction**	1.773895	0.040639	0.355502
**somatosensory**	1.298502	0.054760	0.355502
**emotion regulation**	1.500876	0.063483	0.355502
**executive function**	1.446357	0.117080	0.546372
**body**	1.213940	0.151772	0.607088
**threat**	1.240375	0.196280	0.627132
**prediction**	1.173262	0.221819	0.627132
**inhibition**	1.131664	0.240563	0.627132
**working memory**	1.096277	0.246373	0.627132
**cognitive control**	1.087996	0.300346	0.686571
**perception**	1.053513	0.327367	0.686571
**anxiety**	1.062665	0.343286	0.686571
**decision making**	1.040149	0.372981	0.696231
**action**	1.016013	0.417886	0.728021
**theory mind**	0.962374	0.442013	0.728021
**sensorimotor**	0.893723	0.671055	0.988580
**learning**	0.905023	0.695114	0.988580
**reward**	0.876946	0.706128	0.988580
**impulsivity**	1.357504	1.000000	1.000000
**compulsive**	0.377431	1.000000	1.000000
**eating**	0.708028	1.000000	1.000000
**interoceptive**	1.383914	1.000000	1.000000
**addiction**	1.179981	1.000000	1.000000
**food**	0.841647	1.000000	1.000000
**obsessive**	0.414529	1.000000	1.000000
**punishment**	0.740714	1.000000	1.000000

Supplementary Table 9. Functional decoding results for reverse inference.

	probReverse	pReverse	qReverse
**term**			
**somatosensory**	0.068604	0.005196	0.145488
**social interaction**	0.016269	0.032389	0.360706
**flexibility**	0.018026	0.038647	0.360706
**reinforcement**	0.020310	0.062096	0.434674
**emotion regulation**	0.029059	0.170808	0.956525
**reward**	0.063379	0.292043	1.000000
**body**	0.052527	0.367367	1.000000
**executive function**	0.017460	0.389899	1.000000
**working memory**	0.093754	0.392029	1.000000
**action**	0.070563	0.478892	1.000000
**prediction**	0.033568	0.571397	1.000000
**threat**	0.022168	0.589050	1.000000
**sensorimotor**	0.047918	0.607765	1.000000
**anxiety**	0.037401	0.615991	1.000000
**perception**	0.105539	0.661964	1.000000
**theory mind**	0.013654	0.674466	1.000000
**inhibition**	0.053313	0.799004	1.000000
**decision making**	0.041501	0.828451	1.000000
**learning**	0.081158	0.877936	1.000000
**cognitive control**	0.051000	0.964089	1.000000
**impulsivity**	0.012769	1.000000	1.000000
**compulsive**	0.003284	1.000000	1.000000
**eating**	0.005661	1.000000	1.000000
**interoceptive**	0.008787	1.000000	1.000000
**addiction**	0.012487	1.000000	1.000000
**food**	0.011282	1.000000	1.000000
**obsessive**	0.003217	1.000000	1.000000
**punishment**	0.005342	1.000000	1.000000

Supplementary Table 10. Results for spatial correlations between ALE map "GM_loss_acute" and Neurosynth meta-analytic maps for AN-related topics.

	rho	p	q
**eating**	-0.554191	0.0001	0.001400
**food**	-0.499862	0.0001	0.001400
**threat**	-0.519304	0.0010	0.009333
**reinforcement**	-0.377402	0.0054	0.037800
**action**	0.391839	0.0226	0.123200
**reward**	-0.400036	0.0264	0.123200
**sensorimotor**	0.378182	0.0514	0.172978
**punishment**	-0.417438	0.0522	0.172978
**anxiety**	-0.341374	0.0556	0.172978
**flexibility**	0.279652	0.0650	0.182000
**interoceptive**	-0.213117	0.0912	0.232145
**addiction**	-0.254809	0.1074	0.250600
**compulsive**	-0.281116	0.1470	0.316615
**obsessive**	-0.276892	0.1702	0.340400
**working memory**	0.148983	0.2730	0.461689
**emotion regulation**	-0.300834	0.2880	0.461689
**impulsivity**	-0.256502	0.2942	0.461689
**social interaction**	-0.137690	0.2968	0.461689
**somatosensory**	0.184974	0.3166	0.464520
**body**	0.190759	0.3318	0.464520
**decision making**	-0.152187	0.5132	0.684267
**learning**	-0.040336	0.7062	0.898800
**inhibition**	0.065287	0.8316	0.976059
**prediction**	-0.023594	0.8860	0.976059
**executive function**	-0.030183	0.8966	0.976059
**theory mind**	0.007741	0.9256	0.976059
**cognitive control**	0.017546	0.9412	0.976059
**perception**	-0.000780	0.9864	0.986400

Supplementary Table 11. Results for spatial correlations between ALE map “GM_loss_acute” and neurotransmitter atlases.

	rho	p	q
**5HTT**	-0.431551	0.0160	0.191100
**NET**	0.349055	0.0182	0.191100
**5HT2a**	-0.361656	0.0606	0.333900
**D1**	-0.284332	0.0636	0.333900
**DAT**	-0.244404	0.1032	0.424667
**FDOPA**	-0.203444	0.1444	0.424667
**5HT1a**	-0.397360	0.1652	0.424667
**GABAa5**	-0.413777	0.1702	0.424667
**5HT4**	-0.271035	0.1820	0.424667
**D2**	-0.282664	0.2388	0.501480
**CB1**	-0.348563	0.3440	0.656727
**MU**	-0.248533	0.5626	0.712271
**5HT1b**	-0.147087	0.5674	0.712271
**GABAa**	-0.127393	0.5710	0.712271
**VAChT**	0.150243	0.5714	0.712271
**NMDA**	-0.064482	0.5748	0.712271
**mGluR5**	0.086829	0.5766	0.712271
**M1**	-0.087381	0.6252	0.729400
**H3**	-0.101602	0.7830	0.851600
**A4B2**	0.079424	0.8176	0.851600
**5HT6**	-0.044200	0.8516	0.851600

3 Supplementary Figures

Supplementary Figure 1. Meta-analyses of studies in acutely ill patients reporting volume scores for gray matter (A), white matter (B) and cerebrospinal fluid (C).

	AN acute			HC				Std. Mean Difference
Study or Subgroup	Mean	SD	Total	Mean	SD	Total	Weight	IV, Random, 95% CI
**1.1.1 GM adults**								
Amianto et al., 2013	558	44	17	559	20	14	2.0%	-0.03 [-0.73, 0.68]
Bär et al., 2015	646.66	57.59	26	701.07	61.63	26	2.6%	-0.90 [-1.47, -0.33]
Boghi et al., 2011	673	49	21	673	49	27	2.6%	0.00 [-0.57, 0.57]
Brooks et al., 2011	88.16	9.54	14	91.8	10.04	21	2.1%	-0.36 [-1.04, 0.32]
Burkert et al., 2015	647	56.8	21	709.4	64.3	21	2.3%	-1.01 [-1.66, -0.36]
D'Agata et al., 2015	595	44	21	596	34	17	2.3%	-0.02 [-0.66, 0.61]
Favaro et al., 2015	606,267.227	48,371.3449	38	625,779.26	40,929.92	38	3.3%	-0.43 [-0.89, 0.02]
Fonville et al., 2014	804,637.8	47,037.5	31	833,791.7	38,115.3	31	2.9%	-0.67 [-1.19, -0.16]
Frank et al., 2013a	651.8	37.9	19	629.7	62.1	24	2.4%	0.41 [-0.20, 1.02]
Friederich et al., 2012	692.8	59.6	12	752	71.9	14	1.7%	-0.86 [-1.67, -0.05]
Halls et al., 2022	644,339	43,517.48	57	665,213.69	50,980.11	68	3.9%	-0.43 [-0.79, -0.08]
Joos et al., 2010	0.478	0.018	12	0.498	0.017	18	1.7%	-1.12 [-1.91, -0.33]
Kaufmann et al., 2020	505.95	31.01	24	533.36	42.26	30	2.7%	-0.72 [-1.27, -0.16]
Khalsa et al., 2016	717	91	12	779	127	30	2.1%	-0.51 [-1.19, 0.16]
Kohmura et al., 2017	572.4	41.3	23	634.2	44.9	29	2.4%	-1.40 [-2.02, -0.79]
Lloyd et al., 2023 - study 1	645,360.908	36,792.44205	23	678,332.713	57,870.9376	20	2.4%	-0.68 [-1.30, -0.06]
Lloyd et al., 2023 - study 2	635,095.957	46,860.06893	26	659,712.685	40,680.7681	25	2.7%	-0.55 [-1.11, 0.01]
Lloyd et al., 2023 - study 3	628,004.287	59,413.76256	16	644,644.692	39,963.9215	13	1.9%	-0.31 [-1.05, 0.42]
Lloyd et al., 2023 - study 4	610,266.355	33,812.1236	30	644,334.573	51,067.2116	35	3.0%	-0.77 [-1.27, -0.26]
Mishima et al., 2021	561.21	47.97	35	595.82	35.3	35	3.1%	-0.81 [-1.30, -0.32]
Nickel et al., 2018	678.6	60.4	34	721.7	55	41	3.2%	-0.74 [-1.21, -0.27]
Roberto et al., 2011	647.63	62.07	32	679.93	53.31	21	2.7%	-0.54 [-1.10, 0.02]
Scaife et al., 2017	40	4.5	12	43	1.1	16	1.7%	-0.96 [-1.75, -0.16]
Suchan et al., 2010	656.1	70.9	15	673.2	76.1	15	2.0%	-0.23 [-0.94, 0.49]
Swayze et al., 2003	763.55	61.04	18	782.81	77.09	18	2.2%	-0.27 [-0.93, 0.39]
Via et al., 2014	643.13	48.73	19	652.07	43.93	19	2.3%	-0.19 [-0.83, 0.45]
Yue et al., 2018	637.98	48.51	35	678.92	41.43	20	2.6%	-0.88 [-1.45, -0.30]
Yu et al., 2024	685.74	44.38	64	704.98	49.15	56	3.9%	-0.41 [-0.77, -0.05]
**Subtotal (95% CI)**			**707**			**742**	**70.5%**	**-0.55 [-0.68, -0.41]**
Heterogeneity: Tau² = 0.04; Chi² = 41.19, df = 27 (P = 0.04); I² = 34%								
Test for overall effect: Z = 7.93 (P < 0.00001)								
**1.1.2 GM adolescents**								
Bomba et al., 2013	586,215	60,592	11	689,829	64,033	8	1.1%	-1.60 [-2.67, -0.52]
Castro-Fornieles et al., 2009	637.9	62.5	12	746.9	26	9	1.0%	-2.07 [-3.18, -0.96]
Curzio et al., 2020	650	40	24	680	340	24	2.6%	-0.12 [-0.69, 0.44]
Doose et al., 2023	608,743.94	49,564.22	30	663,476.12	77,043.36	30	2.8%	-0.83 [-1.36, -0.30]
Frank et al., 2013b	662.62	44.88	19	667.38	68.33	22	2.4%	-0.08 [-0.69, 0.53]
Fujisawa et al., 2015	686.08	66.2	20	762.11	35.02	14	1.8%	-1.33 [-2.09, -0.57]
Gaudio et al., 2011	697	68	16	753	61	16	1.9%	-0.85 [-1.57, -0.12]
Gaudio et al., 2017	642	50	14	674	32	15	1.8%	-0.75 [-1.50, 0.01]
Katzman et al., 1996	317.407	18.831	13	321.858	29.524	8	1.5%	-0.18 [-1.07, 0.70]
King et al., 2015	81,661.49	7,798.71	40	85,513.91	5,933.21	40	3.3%	-0.55 [-1.00, -0.10]
Myrvang et al., 2018	662,812.9	56,607.4	30	717,920.5	59,586.9	28	2.7%	-0.94 [-1.48, -0.39]
Olivo et al., 2018	747.17	61.2	22	754.46	73	38	2.8%	-0.10 [-0.63, 0.42]
Seitz et al., 2015	588.45	47.4	56	633.27	42.25	50	3.6%	-0.99 [-1.39, -0.58]
**Subtotal (95% CI)**			**307**			**302**	**29.5%**	**-0.72 [-0.99, -0.44]**
Heterogeneity: Tau² = 0.14; Chi² = 28.86, df = 12 (P = 0.004); I² = 58%								
Test for overall effect: Z = 5.17 (P < 0.00001)								
**Total (95% CI)**			**1014**			**1044**	**100.0%**	**-0.59 [-0.72, -0.47]**
Heterogeneity: Tau² = 0.07; Chi² = 71.95, df = 40 (P = 0.001); I² = 44%								
Test for overall effect: Z = 9.34 (P < 0.00001)								
Test for subgroup differences: Chi² = 1.21, df = 1 (P = 0.27), I² = 17.5%								

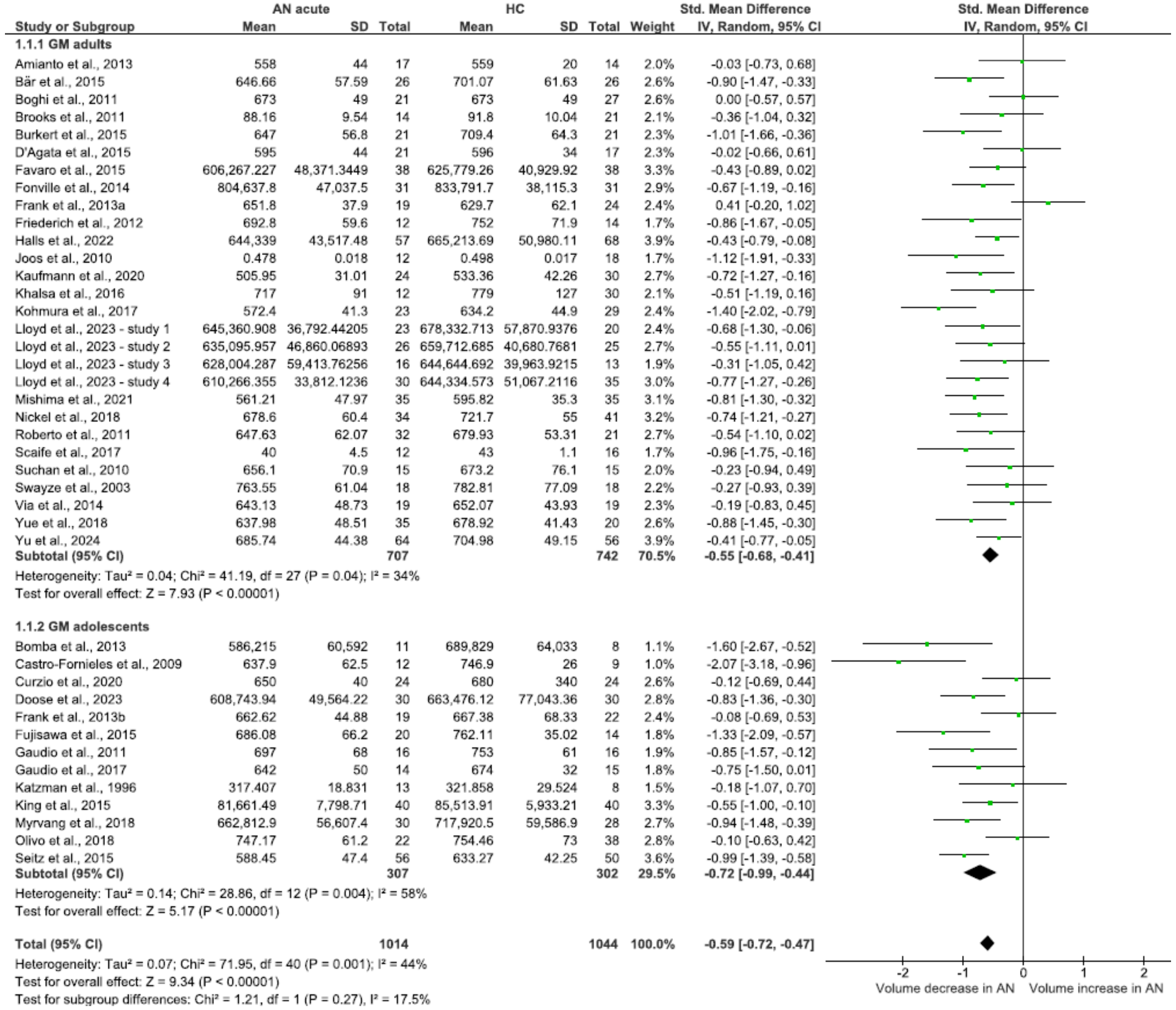

A

Study or Subgroup	AN acute Mean	AN acute SD	AN acute Total	HC Mean	HC SD	HC Total	Weight	Std. Mean Difference IV, Random, 95% CI
**1.2.1 WM adults**								
Amianto et al., 2013	482	31	17	489	26	14	1.8%	-0.24 [-0.95, 0.47]
Bär et al., 2015	469.36	54.59	26	489.2	51.22	26	2.7%	-0.37 [-0.92, 0.18]
Boghi et al., 2011	383	32	21	423	34	27	2.2%	-1.19 [-1.81, -0.57]
Brooks et al., 2011	54.09	4.23	14	52.25	5.54	21	1.9%	0.35 [-0.33, 1.04]
Burkert et al., 2015	455.4	66	21	471.3	54.3	21	2.3%	-0.26 [-0.87, 0.35]
D'Agata et al., 2015	496	34	21	490	54	17	2.1%	0.13 [-0.51, 0.77]
Favaro et al., 2015	433,766.679	56,689.3333	38	429,071.34	47,002.49	38	3.7%	0.09 [-0.36, 0.54]
Fonville et al., 2014	712,710.2	32,691.4	31	719,545.3	29,275.7	31	3.2%	-0.22 [-0.72, 0.28]
Frank et al., 2013a	503.8	41	19	494	44.3	24	2.3%	0.22 [-0.38, 0.83]
Friederich et al., 2012	450.9	46.9	12	466.8	68.3	14	1.5%	-0.26 [-1.03, 0.52]
Halls et al., 2022	446,351.16	49,469.46	57	454,645.4	43,570.36	68	5.2%	-0.18 [-0.53, 0.17]
Joos et al., 2010	0.308	0.018	12	0.317	0.02	18	1.6%	-0.46 [-1.20, 0.29]
Kaufmann et al., 2020	438.1	35.55	24	450.82	51.94	30	2.8%	-0.28 [-0.82, 0.26]
Khalsa et al., 2016	434	47	12	490	100	30	1.9%	-0.62 [-1.30, 0.06]
Kohmura et al., 2017	521.1	52.5	23	528.7	48.6	29	2.7%	-0.15 [-0.70, 0.40]
Lloyd et al., 2023 - study 1	427,961.328	38,293.16672	23	431,434.971	42,506.9574	20	2.4%	-0.08 [-0.68, 0.51]
Lloyd et al., 2023 - study 2	425,647.447	42,965.83633	26	428,863.395	46,864.1234	25	2.7%	-0.07 [-0.62, 0.48]
Lloyd et al., 2023 - study 3	440,925.251	51,728.14676	16	420,428.782	37,158.6699	13	1.6%	0.43 [-0.31, 1.18]
Lloyd et al., 2023 - study 4	427,729.752	42,611.22353	30	436,671.537	39,983.3893	35	3.3%	-0.21 [-0.70, 0.27]
Mishima et al., 2021	462.94	41.76	35	477.9	36.89	35	3.5%	-0.38 [-0.85, 0.10]
Nickel et al., 2018	479	38.9	34	504.6	43.7	41	3.5%	-0.61 [-1.07, -0.14]
Roberto et al., 2011	479.22	51.42	32	488.22	53.68	21	2.7%	-0.17 [-0.72, 0.38]
Scaife et al., 2017	37.1	1.3	12	37.5	1.4	16	1.6%	-0.29 [-1.04, 0.47]
Suchan et al., 2010	453.5	47.3	15	481.3	50.9	15	1.7%	-0.55 [-1.28, 0.18]
Swayze et al., 2003	452.37	45.06	18	499.05	73.52	18	1.9%	-0.75 [-1.43, -0.07]
Via et al., 2014	457.6	35.22	19	474.19	34.54	19	2.1%	-0.47 [-1.11, 0.18]
Yu et al., 2024	474.01	45.68	64	470.21	39.9	56	5.1%	0.09 [-0.27, 0.45]
**Subtotal (95% CI)**			**672**			**722**	**70.1%**	**-0.22 [-0.34, -0.10]**
Heterogeneity: Tau² = 0.02; Chi² = 32.38, df = 26 (P = 0.18); I² = 20%								
Test for overall effect: Z = 3.60 (P = 0.0003)								
**1.2.2 WM adolescents**								
Bomba et al., 2013	465,369	44,689	11	521,839	23,645	8	0.9%	-1.44 [-2.48, -0.39]
Castro-Fornieles et al., 2009	449.8	49.8	12	499.8	47.6	9	1.1%	-0.98 [-1.91, -0.06]
Curzio et al., 2020	440	30	24	460	30	24	2.5%	-0.66 [-1.24, -0.07]
Doose et al., 2023	417,805.9	44,712.72	30	425,248.448	49,350.15	30	3.1%	-0.16 [-0.66, 0.35]
Frank et al., 2013b	459.79	44.91	19	472.28	45.53	22	2.2%	-0.27 [-0.89, 0.35]
Gaudio et al., 2017	466	55	14	481	39	15	1.7%	-0.31 [-1.04, 0.43]
Geisler et al., 2022	419.4	40	96	429.6	42.1	96	6.8%	-0.25 [-0.53, 0.04]
Katzman et al., 1996	212.825	16.733	13	209.868	26.779	8	1.2%	0.14 [-0.75, 1.02]
Myrvang et al., 2018	417,027.1	47,223	30	436,765	46,027.6	28	3.0%	-0.42 [-0.94, 0.10]
Olivo et al., 2018	388.61	31.2	22	395.94	40.22	38	2.9%	-0.19 [-0.72, 0.33]
Seitz et al., 2015	416.93	35.29	56	437.42	40.07	50	4.6%	-0.54 [-0.93, -0.15]
**Subtotal (95% CI)**			**327**			**328**	**29.9%**	**-0.37 [-0.53, -0.21]**
Heterogeneity: Tau² = 0.00; Chi² = 10.61, df = 10 (P = 0.39); I² = 6%								
Test for overall effect: Z = 4.42 (P < 0.00001)								
**Total (95% CI)**			**999**			**1050**	**100.0%**	**-0.27 [-0.37, -0.17]**
Heterogeneity: Tau² = 0.02; Chi² = 45.43, df = 37 (P = 0.16); I² = 19%								
Test for overall effect: Z = 5.26 (P < 0.00001)								
Test for subgroup differences: Chi² = 2.02, df = 1 (P = 0.16), I² = 50.4%								

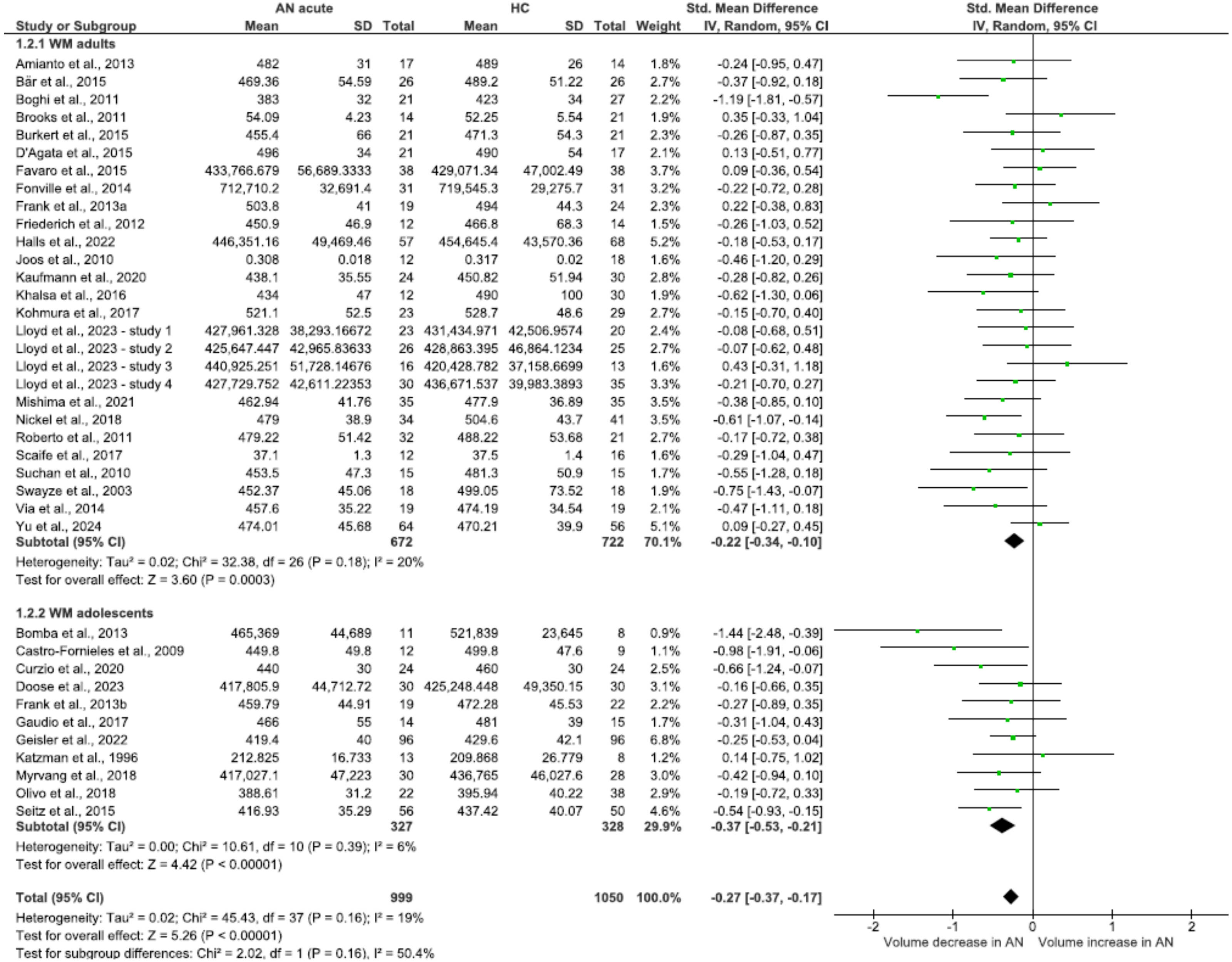

B

Study or Subgroup	AN acute Mean	AN acute SD	AN acute Total	HC Mean	HC SD	HC Total	Weight	Std. Mean Difference IV, Random, 95% CI
**1.3.1 CSF adults**								
Boghi et al., 2011	315	39	21	301	59	27	5.1%	0.27 [-0.30, 0.84]
D'Agata et al., 2015	210	21	21	188	19	17	4.6%	1.07 [0.38, 1.76]
Fonville et al., 2014	37,956.5	7,764.1	31	33,639.6	10,572.3	31	5.4%	0.46 [-0.05, 0.96]
Frank et al., 2013a	246.3	33.1	19	226.8	30.6	24	4.9%	0.60 [-0.01, 1.22]
Friederich et al., 2012	276.8	50.2	12	252.8	40	14	4.1%	0.52 [-0.27, 1.30]
Joos et al., 2010	0.214	0.032	12	0.186	0.027	18	4.2%	0.94 [0.16, 1.71]
Khalsa et al., 2016	443	60	12	458	129	30	4.6%	-0.13 [-0.80, 0.54]
Kohmura et al., 2017	244.8	35.7	23	198.3	21	29	4.8%	1.61 [0.98, 2.25]
Mishima et al., 2021	311.47	62.82	35	251.76	46.78	35	5.4%	1.07 [0.56, 1.57]
Nickel et al., 2018	376.9	53.7	34	341.8	36.2	41	5.5%	0.77 [0.30, 1.24]
Roberto et al., 2011	276.57	47.61	32	229.05	45.24	21	5.0%	1.00 [0.42, 1.59]
Scaife et al., 2017	22.2	2.3	12	19.5	1.6	16	3.9%	1.36 [0.52, 2.20]
Suchan et al., 2010	749.2	91.1	15	753.3	98.1	15	4.4%	-0.04 [-0.76, 0.67]
Swayze et al., 2003	187.63	56.7	18	96.51	26.48	18	4.0%	2.01 [1.20, 2.83]
Yu et al., 2024	257.05	55.22	64	222.77	33.38	56	5.9%	0.74 [0.36, 1.11]
**Subtotal (95% CI)**			**361**			**392**	**71.8%**	**0.79 [0.54, 1.05]**
Heterogeneity: Tau² = 0.15; Chi² = 37.32, df = 14 (P = 0.0007); I² = 62%								
Test for overall effect: Z = 6.08 (P < 0.00001)								
**1.3.2 CSF adolescents**								
Bomba et al., 2013	315,480	55,098	11	221,033	24,736	8	2.8%	2.00 [0.84, 3.16]
Castro-Fornieles et al., 2009	216.5	34.2	12	163.5	27	9	3.3%	1.62 [0.60, 2.64]
Curzio et al., 2020	252	18	24	242	15	24	5.0%	0.59 [0.01, 1.17]
Frank et al., 2013b	230.74	24.16	19	252.35	49.23	22	4.8%	-0.53 [-1.16, 0.09]
Gaudio et al., 2017	210	22	14	194	18	15	4.3%	0.78 [0.02, 1.54]
Katzman et al., 1996	77.319	14.322	13	45.961	6.584	8	2.7%	2.50 [1.28, 3.71]
Olivo et al., 2018	165.37	37.01	22	156.28	35.13	38	5.3%	0.25 [-0.28, 0.78]
**Subtotal (95% CI)**			**115**			**124**	**28.2%**	**0.91 [0.23, 1.59]**
Heterogeneity: Tau² = 0.66; Chi² = 33.23, df = 6 (P < 0.00001); I² = 82%								
Test for overall effect: Z = 2.61 (P = 0.009)								
**Total (95% CI)**			**476**			**516**	**100.0%**	**0.80 [0.55, 1.06]**
Heterogeneity: Tau² = 0.25; Chi² = 72.34, df = 21 (P < 0.00001); I² = 71%								
Test for overall effect: Z = 6.13 (P < 0.00001)								
Test for subgroup differences: Chi² = 0.09, df = 1 (P = 0.76), I² = 0%								

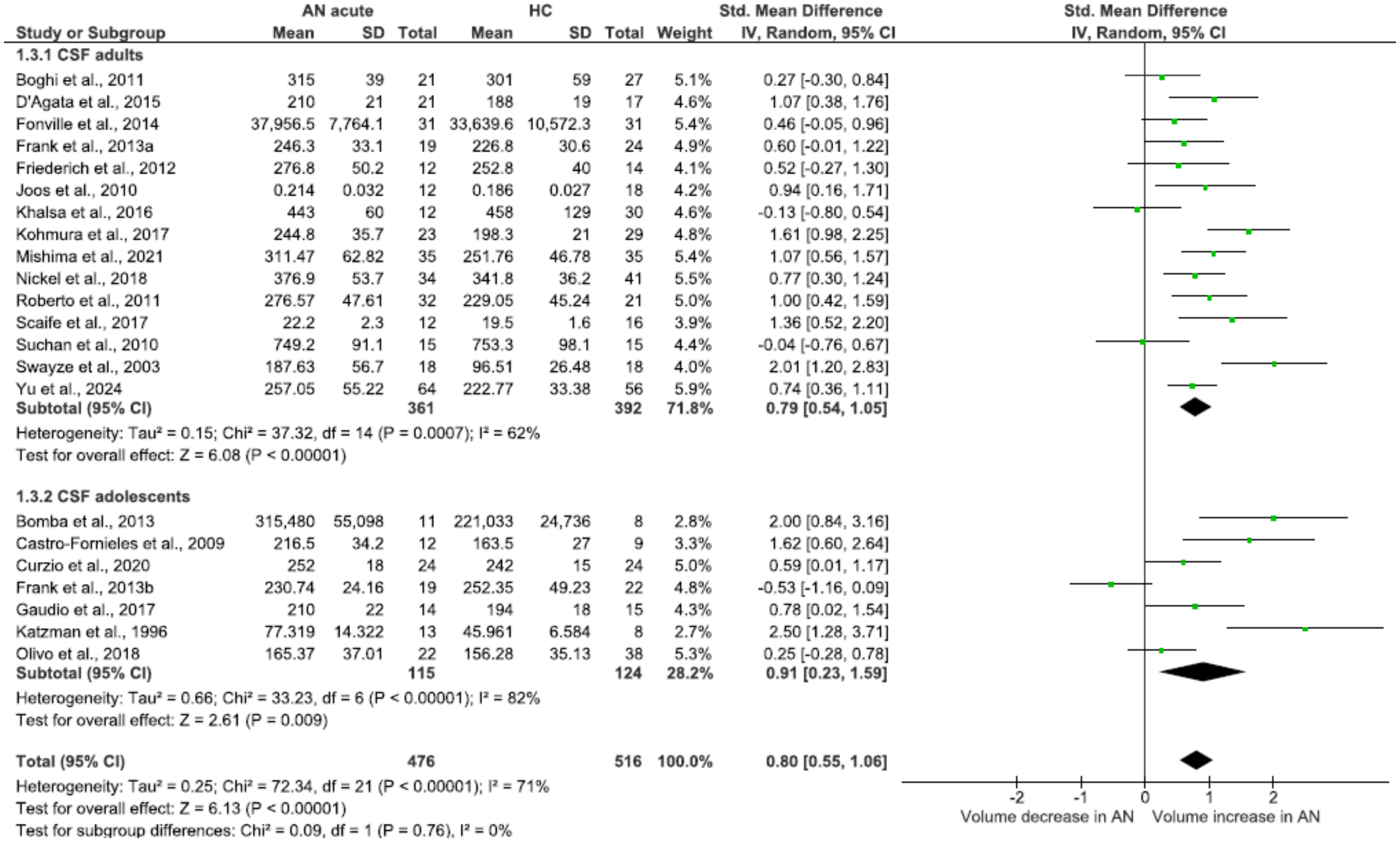

C

Supplementary Figure 2. Meta-analyses of studies in short-term weight recovered patients reporting volume scores for gray matter (A), white matter (B) and cerebrospinal fluid (C).

	AN short-rec			HC				Std. Mean Difference
**Study or Subgroup**	**Mean**	**SD**	**Total**	**Mean**	**SD**	**Total**	**Weight**	**IV, Random, 95% CI**
**2.1.1 GM adults**								
Frank et al., 2013a	639.4	49.5	24	629.7	62.1	24	12.0%	0.17 [-0.40, 0.74]
Kaufmann et al., 2020	535.52	38.83	26	531.65	39.21	30	12.7%	0.10 [-0.43, 0.62]
Khalsa et al., 2016	727	63	20	779	127	30	11.8%	-0.48 [-1.06, 0.09]
Roberto et al., 2011	662.64	69.71	32	680.91	52.35	21	12.2%	-0.28 [-0.84, 0.27]
Swayze et al., 2003	778.7	63.74	13	782.81	77.09	18	9.6%	-0.06 [-0.77, 0.66]
**Subtotal (95% CI)**			**115**			**123**	**58.3%**	**-0.11 [-0.37, 0.15]**
Heterogeneity: Tau² = 0.00; Chi² = 3.54, df = 4 (P = 0.47); I² = 0%								
Test for overall effect: Z = 0.82 (P = 0.41)								
**2.1.2 GM adolescents**								
Bomba et al., 2015	626,685	100,600	11	689,829	64,033	8	6.8%	-0.69 [-1.63, 0.25]
Castro-Fornieles et al., 2009	660.7	68.3	12	731.9	41.1	9	6.7%	-1.17 [-2.12, -0.22]
Katzman et al., 1997	584.9	40.4	6	562.6	51.4	16	6.7%	0.44 [-0.51, 1.39]
Lazaro et al., 2013	779.5	53.8	35	781.2	51	17	11.7%	-0.03 [-0.61, 0.55]
Mainz et al., 2012	638	72	18	738	99	19	9.8%	-1.13 [-1.82, -0.43]
**Subtotal (95% CI)**			**82**			**69**	**41.7%**	**-0.51 [-1.12, 0.09]**
Heterogeneity: Tau² = 0.30; Chi² = 11.39, df = 4 (P = 0.02); I² = 65%								
Test for overall effect: Z = 1.65 (P = 0.10)								
**Total (95% CI)**			**197**			**192**	**100.0%**	**-0.27 [-0.57, 0.02]**
Heterogeneity: Tau² = 0.11; Chi² = 17.73, df = 9 (P = 0.04); I² = 49%								
Test for overall effect: Z = 1.80 (P = 0.07)								
Test for subgroup differences: Chi² = 1.44, df = 1 (P = 0.23), I² = 30.6%								

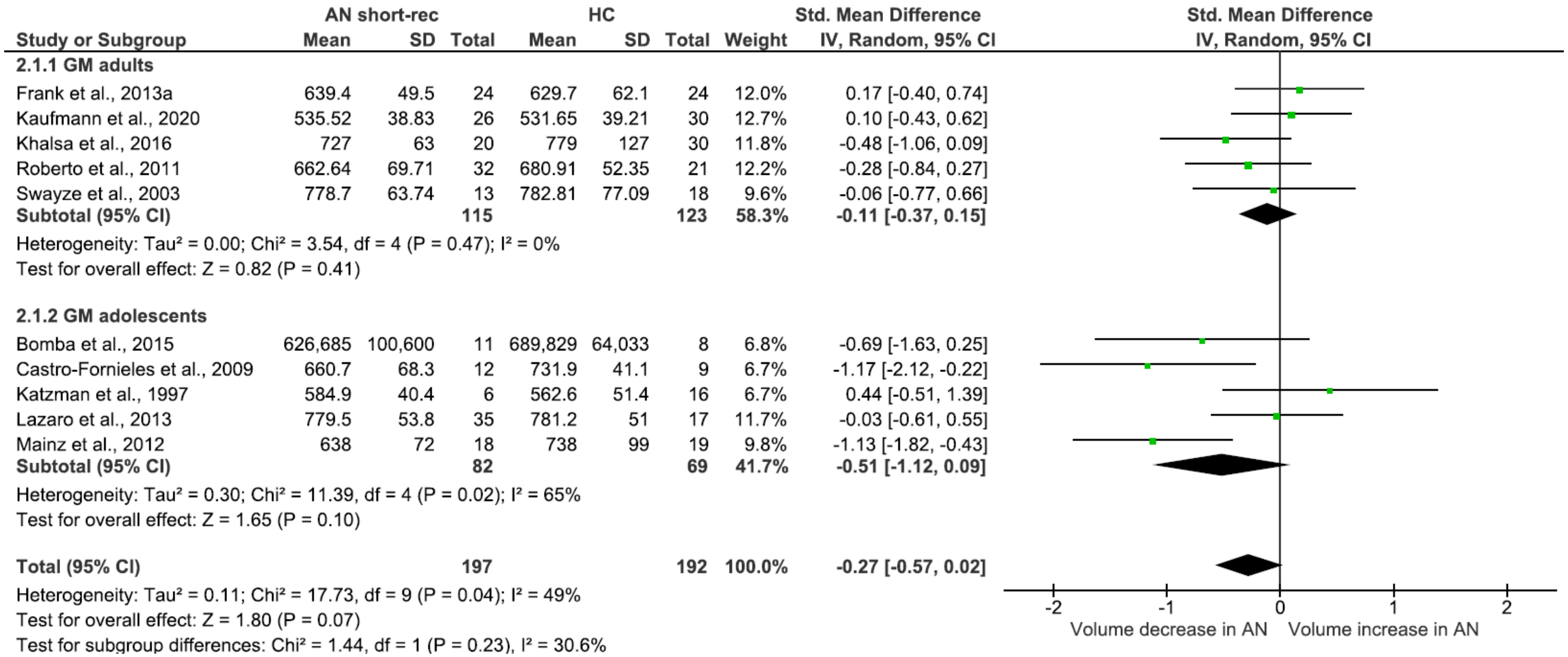

A

Study or Subgroup	AN short-rec Mean	AN short-rec SD	AN short-rec Total	HC Mean	HC SD	HC Total	Weight	Std. Mean Difference IV, Random, 95% CI
**2.2.1 WM adults**								
Frank et al., 2013a	493	56.3	24	494	44.3	24	12.9%	-0.02 [-0.59, 0.55]
Kaufmann et al., 2020	442.73	37.17	26	447.36	50.06	30	14.6%	-0.10 [-0.63, 0.42]
Khalsa et al., 2016	451	39	20	490	100	30	12.6%	-0.47 [-1.04, 0.10]
Roberto et al., 2011	489.99	51.04	32	488.36	55.54	21	13.5%	0.03 [-0.52, 0.58]
Swayze et al., 2003	488.44	54.05	13	499.05	73.52	18	8.6%	-0.16 [-0.87, 0.56]
**Subtotal (95% CI)**			**115**			**123**	**62.2%**	**-0.14 [-0.40, 0.12]**
Heterogeneity: Tau² = 0.00; Chi² = 1.84, df = 4 (P = 0.76); I² = 0%								
Test for overall effect: Z = 1.04 (P = 0.30)								
**2.2.2 WM adolescents**								
Bomba et al., 2015	481,015	44,491	11	521,839	23,645	8	4.7%	-1.04 [-2.03, -0.06]
Castro-Fornieles et al., 2009	457.6	53.3	12	503.1	42.7	9	5.4%	-0.89 [-1.80, 0.03]
Katzman et al., 1997	422.2	52.1	6	391	44.6	16	5.0%	0.64 [-0.32, 1.61]
Lazaro et al., 2013	528.3	37.3	35	532.4	39.2	17	12.4%	-0.11 [-0.69, 0.47]
Mainz et al., 2012	435	38	18	439	54	19	10.3%	-0.08 [-0.73, 0.56]
**Subtotal (95% CI)**			**82**			**69**	**37.8%**	**-0.26 [-0.77, 0.24]**
Heterogeneity: Tau² = 0.17; Chi² = 8.18, df = 4 (P = 0.09); I² = 51%								
Test for overall effect: Z = 1.02 (P = 0.31)								
**Total (95% CI)**			**197**			**192**	**100.0%**	**-0.17 [-0.39, 0.05]**
Heterogeneity: Tau² = 0.01; Chi² = 10.18, df = 9 (P = 0.34); I² = 12%								
Test for overall effect: Z = 1.55 (P = 0.12)								
Test for subgroup differences: Chi² = 0.19, df = 1 (P = 0.66), I² = 0%								

Std. Mean Difference IV, Random, 95% CI

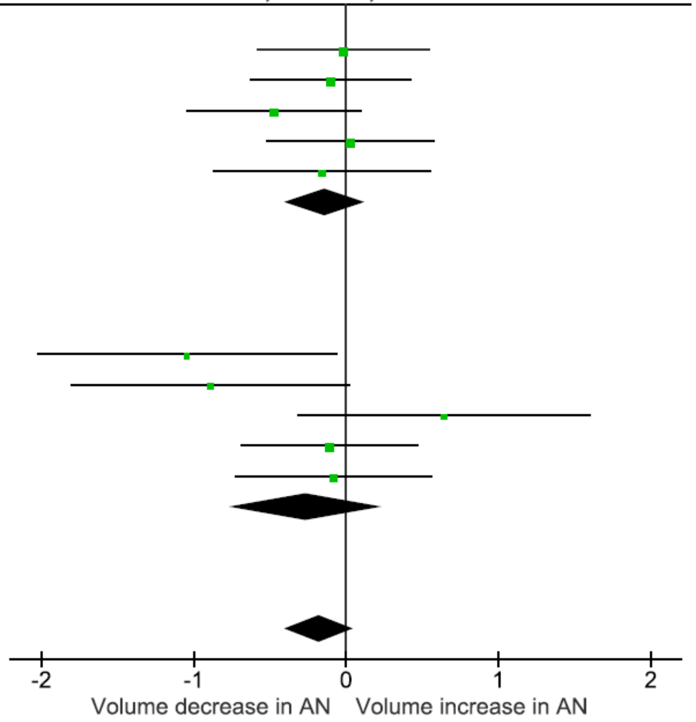

B

Study or Subgroup	AN short-rec Mean	AN short-rec SD	AN short-rec Total	HC Mean	HC SD	HC Total	Weight	Std. Mean Difference IV, Random, 95% CI
**2.3.1 CSF adults**								
Frank et al., 2013a	226.5	26.6	24	226.8	30.6	24	12.9%	-0.01 [-0.58, 0.56]
Khalsa et al., 2016	415	91	20	458	129	30	12.9%	-0.37 [-0.94, 0.20]
Roberto et al., 2011	267.7	49.2	32	229.02	40.82	21	12.8%	0.83 [0.25, 1.40]
Swayze et al., 2003	130.75	38.5	13	96.51	26.48	18	10.7%	1.04 [0.28, 1.81]
**Subtotal (95% CI)**			**89**			**93**	**49.3%**	**0.35 [-0.29, 0.99]**
Heterogeneity: Tau² = 0.33; Chi² = 13.23, df = 3 (P = 0.004); I² = 77%								
Test for overall effect: Z = 1.06 (P = 0.29)								
**2.3.2 CSF adolescents**								
Bomba et al., 2015	252,179	52,168	11	221,033	24,736	8	9.0%	0.69 [-0.25, 1.63]
Castro-Fornieles et al., 2009	175.4	17.7	12	175.4	15.9	9	9.8%	0.00 [-0.86, 0.86]
Katzman et al., 1997	118.4	18.6	6	83.6	15	16	7.3%	2.10 [0.93, 3.26]
Lazaro et al., 2013	307.6	24.3	35	306.7	21.3	17	12.8%	0.04 [-0.54, 0.62]
Mainz et al., 2012	455	59	18	422	52	19	11.9%	0.58 [-0.08, 1.24]
**Subtotal (95% CI)**			**82**			**69**	**50.7%**	**0.58 [-0.02, 1.17]**
Heterogeneity: Tau² = 0.29; Chi² = 11.11, df = 4 (P = 0.03); I² = 64%								
Test for overall effect: Z = 1.88 (P = 0.06)								
**Total (95% CI)**			**171**			**162**	**100.0%**	**0.46 [0.05, 0.87]**
Heterogeneity: Tau² = 0.26; Chi² = 24.80, df = 8 (P = 0.002); I² = 68%								
Test for overall effect: Z = 2.18 (P = 0.03)								
Test for subgroup differences: Chi² = 0.26, df = 1 (P = 0.61), I² = 0%								

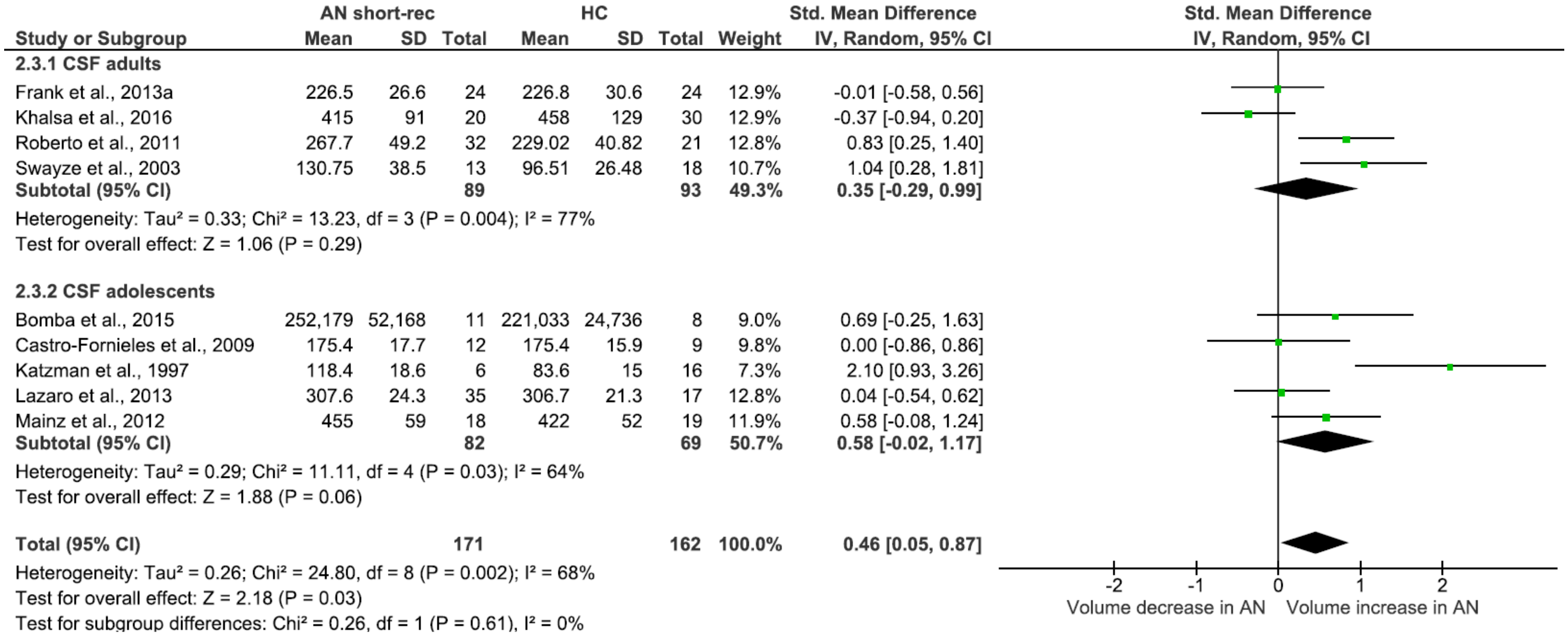

C

Supplementary Figure 3. Meta-analysis of studies in longer-term recovered patients reporting volume scores for gray matter (A), white matter (B) and cerebrospinal fluid (C) showing significant remaining gray matter volume reductions after 1.5 years of recovery.

Study or Subgroup	AN longer-rec Mean	AN longer-rec SD	AN longer-rec Total	HC Mean	HC SD	HC Total	Weight	Std. Mean Difference IV, Random, 95% CI
**3.1.1 GM adults**								
Bang et al., 2016	635.96	52.03	22	664.39	44.81	22	8.6%	-0.58 [-1.18, 0.03]
Favaro et al., 2015	625,340.44	43,260.69	20	625,779.257	40,929.9227	38	10.2%	-0.01 [-0.55, 0.53]
Friederich et al., 2012	724.4	70.7	13	752	71.9	14	5.8%	-0.38 [-1.14, 0.39]
Joos et al., 2011	0.49	0.013	5	0.498	0.017	18	3.5%	-0.47 [-1.47, 0.53]
Lambe et al., 1997	312.305	27.033	12	307.383	33.849	18	6.2%	0.15 [-0.58, 0.88]
Mühlau et al., 2007	0.379	0.01	22	0.392	0.02	37	10.1%	-0.75 [-1.30, -0.21]
Nickel et al., 2018	698.4	54.3	24	721.7	55	41	11.2%	-0.42 [-0.93, 0.09]
Oliva et al., 2020	629.39	42.13	15	636.74	55.61	15	6.5%	-0.14 [-0.86, 0.57]
Scaife et al., 2017	42.2	1.6	14	43	1.1	16	6.2%	-0.57 [-1.31, 0.16]
Wagner et al., 2006	745.05	54.3	30	729.3	45.4	31	11.3%	0.31 [-0.19, 0.82]
**Subtotal (95% CI)**			**177**			**250**	**79.6%**	**-0.27 [-0.51, -0.03]**
Heterogeneity: Tau² = 0.04; Chi² = 12.58, df = 9 (P = 0.18); I² = 28%								
Test for overall effect: Z = 2.22 (P = 0.03)								
**3.1.2 GM adolescents**								
King et al., 2015	81,607.84	6,335.2	34	84,789.17	6,071.58	34	12.1%	-0.51 [-0.99, -0.02]
Zucker et al., 2017	666.77	51.17	21	690.39	66.26	20	8.3%	-0.39 [-1.01, 0.23]
**Subtotal (95% CI)**			**55**			**54**	**20.4%**	**-0.46 [-0.84, -0.08]**
Heterogeneity: Tau² = 0.00; Chi² = 0.08, df = 1 (P = 0.78); I² = 0%								
Test for overall effect: Z = 2.38 (P = 0.02)								
**Total (95% CI)**			**232**			**304**	**100.0%**	**-0.31 [-0.50, -0.11]**
Heterogeneity: Tau² = 0.02; Chi² = 13.51, df = 11 (P = 0.26); I² = 19%								
Test for overall effect: Z = 3.05 (P = 0.002)								
Test for subgroup differences: Chi² = 0.72, df = 1 (P = 0.40), I² = 0%								

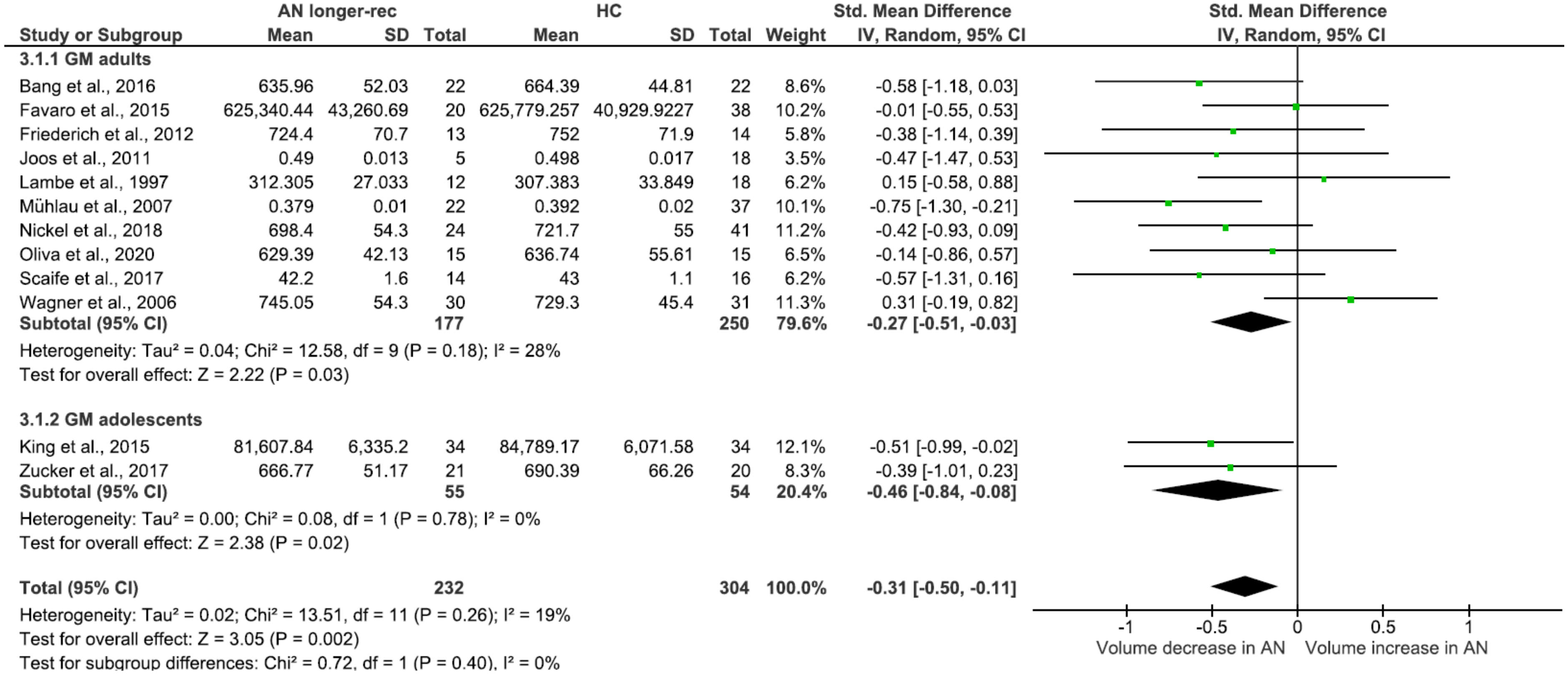

A

Study or Subgroup	AN longer-rec Mean	AN longer-rec SD	AN longer-rec Total	HC Mean	HC SD	HC Total	Weight	Std. Mean Difference IV, Random, 95% CI
**3.2.1 WM adults**								
Bang et al., 2016	474.31	51.71	22	479.7	39.19	22	8.8%	-0.12 [-0.71, 0.48]
Favaro et al., 2015	428,032.44	44,009.87	20	429,071.341	47,002.4867	38	10.4%	-0.02 [-0.56, 0.52]
Friederich et al., 2012	440.8	54	13	466.8	68.3	14	5.2%	-0.41 [-1.17, 0.36]
Joos et al., 2011	0.316	0.011	5	0.317	0.02	18	3.1%	-0.05 [-1.04, 0.94]
Lambe et al., 1997	221.919	20.394	12	213.488	22.018	18	5.6%	0.38 [-0.35, 1.12]
Mühlau et al., 2007	0.247	0.01	22	0.247	0.01	37	11.0%	0.00 [-0.53, 0.53]
Nickel et al., 2018	501	49	24	504.6	43.7	41	12.1%	-0.08 [-0.58, 0.43]
Oliva et al., 2020	515.62	34.44	15	517.86	46.68	15	6.0%	-0.05 [-0.77, 0.66]
Pfuhl et al., 2016	417,829.33	38,367.51	31	444,867.35	39,331.63	31	11.6%	-0.69 [-1.20, -0.17]
Scaife et al., 2017	37.8	1.1	14	37.5	1.4	16	5.9%	0.23 [-0.49, 0.95]
Wagner et al., 2006	393.35	34.15	30	393.6	35.3	31	12.2%	-0.01 [-0.51, 0.49]
**Subtotal (95% CI)**			**208**			**281**	**91.9%**	**-0.10 [-0.28, 0.08]**
Heterogeneity: Tau² = 0.00; Chi² = 8.49, df = 10 (P = 0.58); I² = 0%								
Test for overall effect: Z = 1.09 (P = 0.27)								
**3.2.2 WM adolescents**								
Zucker et al., 2017	535.42	62.3	21	551.81	70.31	20	8.1%	-0.24 [-0.86, 0.37]
**Subtotal (95% CI)**			**21**			**20**	**8.1%**	**-0.24 [-0.86, 0.37]**
Heterogeneity: Not applicable								
Test for overall effect: Z = 0.77 (P = 0.44)								
**Total (95% CI)**			**229**			**301**	**100.0%**	**-0.11 [-0.29, 0.06]**
Heterogeneity: Tau² = 0.00; Chi² = 8.67, df = 11 (P = 0.65); I² = 0%								
Test for overall effect: Z = 1.27 (P = 0.20)								
Test for subgroup differences: Chi² = 0.18, df = 1 (P = 0.67), I² = 0%								

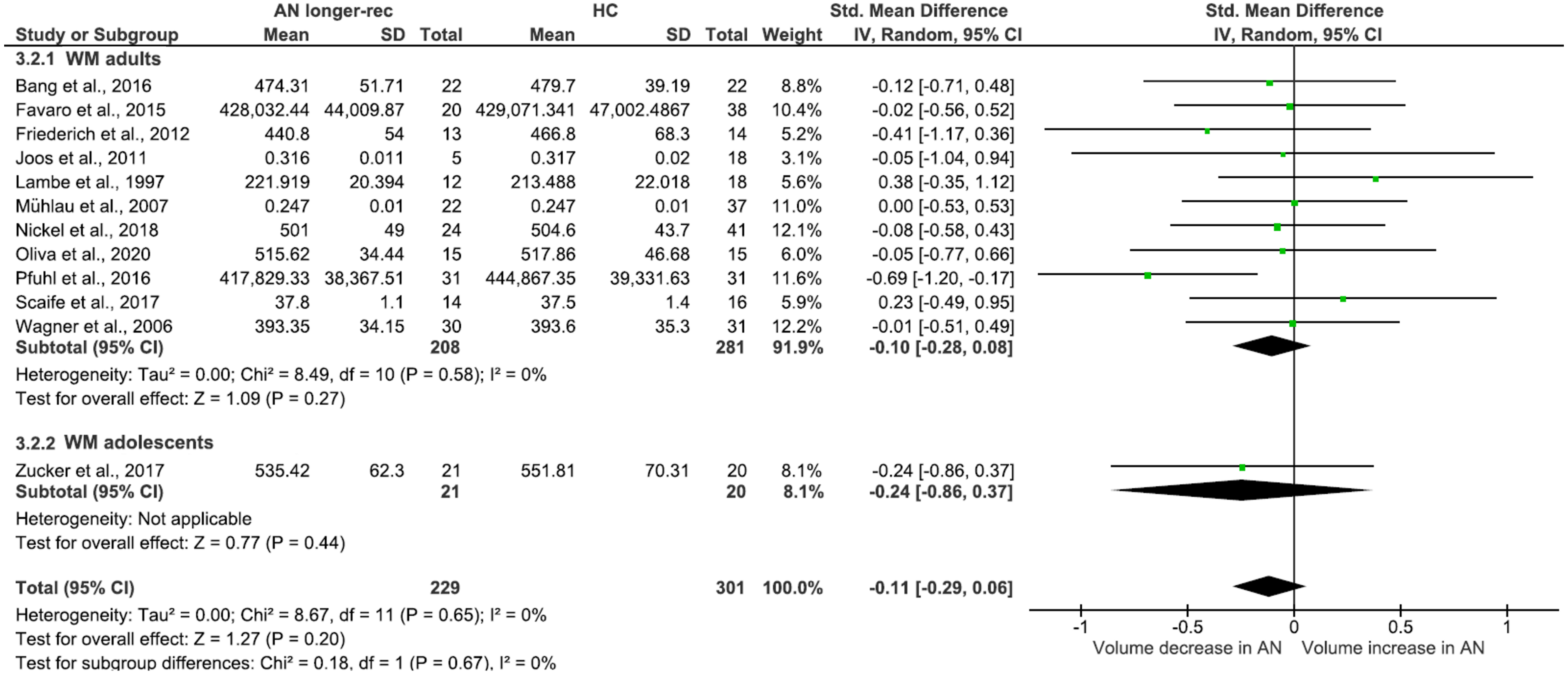

B

Study or Subgroup	AN longer-rec Mean	AN longer-rec SD	AN longer-rec Total	HC Mean	HC SD	HC Total	Weight	Std. Mean Difference IV, Random, 95% CI
**3.3.1 CSF adults**								
Bang et al., 2016	224.82	30.61	22	232.19	21.4	22	11.2%	-0.27 [-0.87, 0.32]
Friederich et al., 2012	265.6	63.1	13	252.8	40	14	8.1%	0.24 [-0.52, 0.99]
Joos et al., 2011	0.194	0.1	5	0.186	0.027	18	5.4%	0.15 [-0.84, 1.15]
Lambe et al., 1997	58.414	16.641	12	47.3	8.421	18	8.0%	0.88 [0.11, 1.65]
Mühlau et al., 2007	0.375	0.02	22	0.361	0.02	37	12.4%	0.69 [0.15, 1.23]
Nickel et al., 2018	329	30.9	24	341.8	36.2	41	13.3%	-0.37 [-0.88, 0.14]
Oliva et al., 2020	267.24	40.09	15	258.76	30.73	15	8.8%	0.23 [-0.49, 0.95]
Scaife et al., 2017	20	2.2	14	19.5	1.6	16	8.7%	0.26 [-0.46, 0.98]
Wagner et al., 2006	325.75	52.4	30	313.4	55.6	31	13.4%	0.23 [-0.28, 0.73]
**Subtotal (95% CI)**			**157**			**212**	**89.2%**	**0.20 [-0.08, 0.48]**
Heterogeneity: Tau² = 0.07; Chi² = 13.40, df = 8 (P = 0.10); I² = 40%								
Test for overall effect: Z = 1.39 (P = 0.16)								
**3.3.2 CSF adolescents**								
Zucker et al., 2017	220.04	24.54	21	223.2	23.23	20	10.8%	-0.13 [-0.74, 0.48]
**Subtotal (95% CI)**			**21**			**20**	**10.8%**	**-0.13 [-0.74, 0.48]**
Heterogeneity: Not applicable								
Test for overall effect: Z = 0.41 (P = 0.68)								
**Total (95% CI)**			**178**			**232**	**100.0%**	**0.16 [-0.09, 0.42]**
Heterogeneity: Tau² = 0.06; Chi² = 14.27, df = 9 (P = 0.11); I² = 37%								
Test for overall effect: Z = 1.24 (P = 0.22)								
Test for subgroup differences: Chi² = 0.91, df = 1 (P = 0.34), I² = 0%								

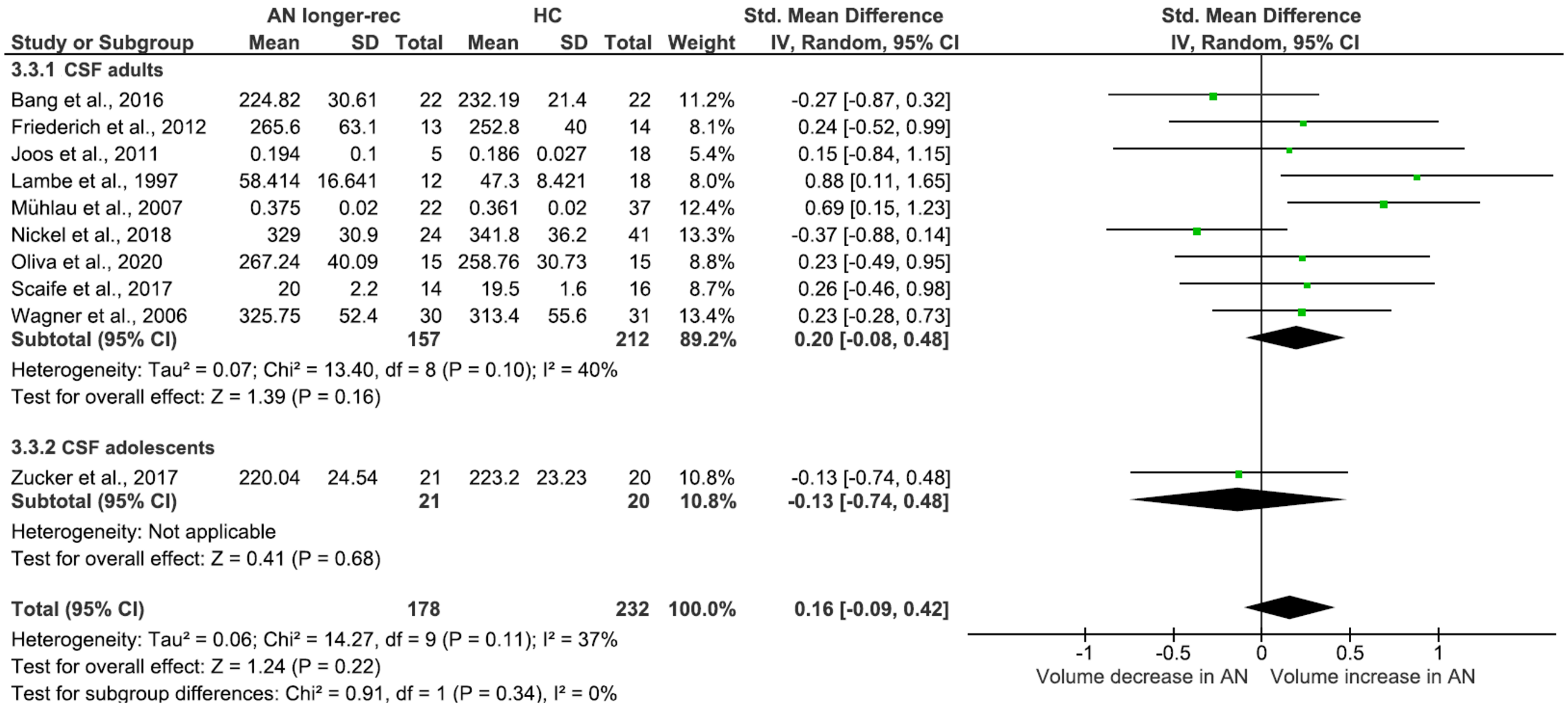

C

Supplementary Figure 4. Results from the directed ALE meta-analysis of structural differences in gray matter volume and cortical thickness in AN displaying TFCE-corrected significant clusters which show reductions in patients with AN compared to healthy controls (analysis "GM_loss_acute_and_recovered"). *ALE*, Anatomical likelihood estimation; *AN*, Anorexia nervosa; *GM*, Gray matter; *TFCE*, Threshold-free cluster enhancement.

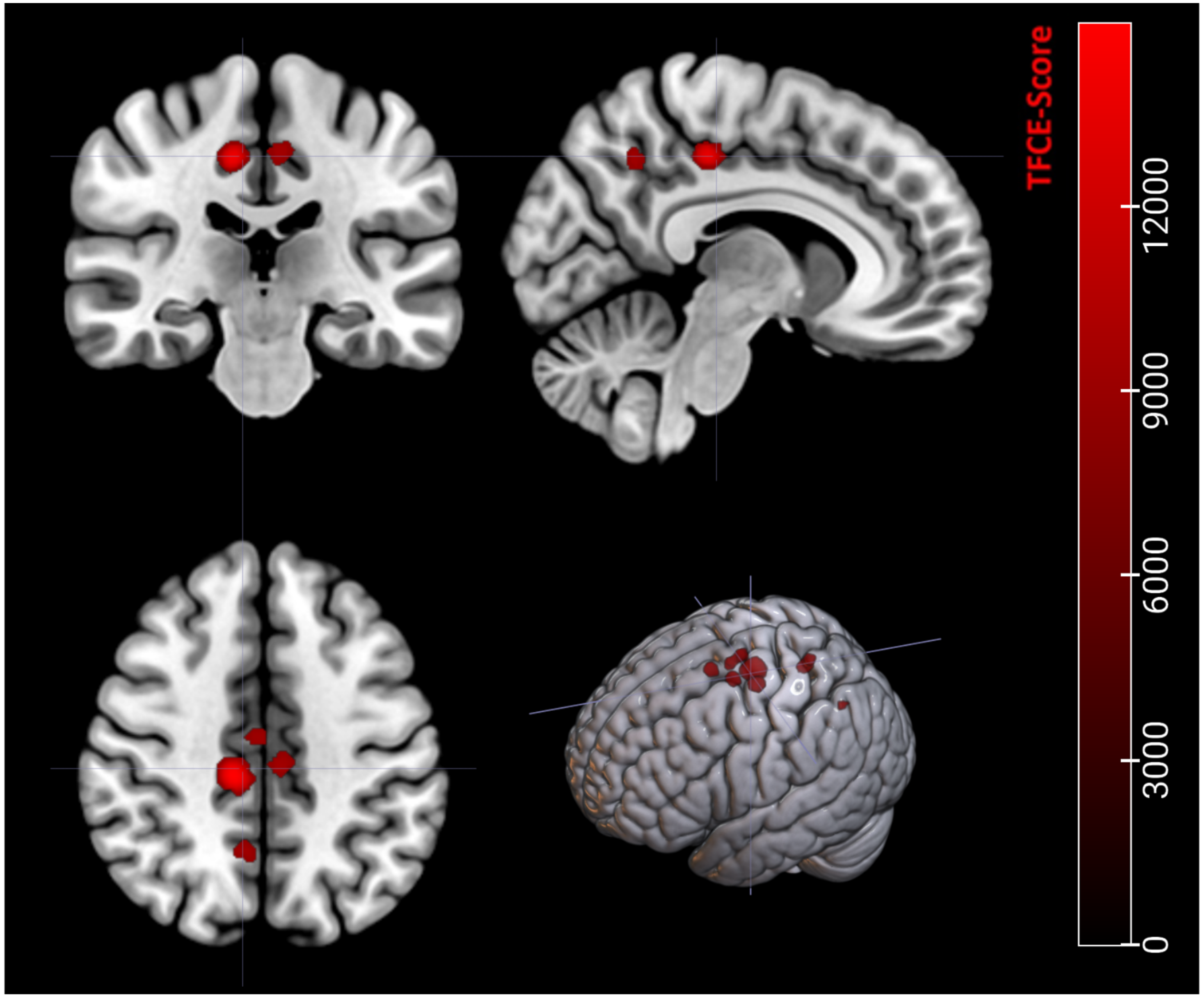

Supplementary Figure 5. Results from the directed ALE meta-analysis of structural differences in gray matter volume and cortical thickness in AN displaying TFCE-corrected significant clusters which show reductions in adult patients with acute AN compared to healthy controls (sub-analysis "GM_loss_acute_adults"). *ALE*, Anatomical likelihood estimation; *AN*, Anorexia nervosa; *GM*, Gray matter; *TFCE*, Threshold-free cluster enhancement.

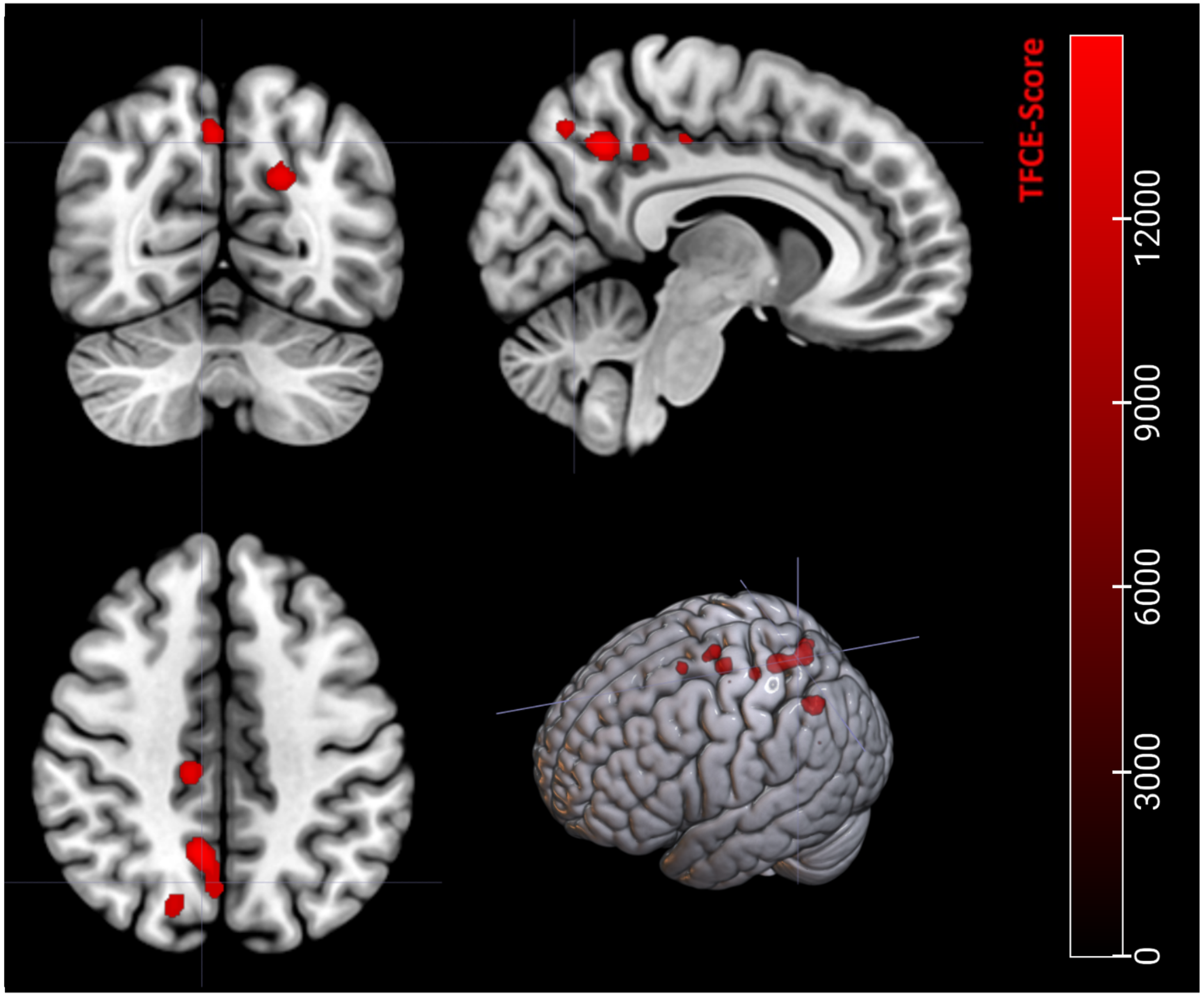

4 Detailed information on risk of bias assessment

As stated in the manuscript, the risk of bias for all included studies was assessed via a modified version of the Newcastle-Ottawa Scale (NOS; https://www.ohri.ca/programs/clinical_epidemiology/oxford.asp, (Wells et al.)) that was adjusted to allow for an evaluation of the methodological quality of cross-sectional studies. In our modified NOS rating, studies were rated on the basis of 8 items in 3 categories including the selection of the sample, the comparability of patients and controls (e.g., regarding age and sex), and the assessment and analysis of our outcome of interest (i.e., brain structure). In this modified NOS rating, a maximum of 9 stars could be achieved.

In some cases, the NOS rating for the same study differs between the global brain volume meta-analysis and the ALE meta-analyses. The reason for this is that in the comparability section, the same study was sometimes rewarded with more stars for the ALE meta-analyses than for the global brain volume meta-analysis, because in the statistical analysis of regional brain structural alterations (i.e., studies included in the ALE meta-analyses) further potentially confounding differences between patients and controls were included as covariates. This additional statistical control of possible confounding factors resulted in a higher NOS rating, but only for studies included in the ALE meta-analyses whereas for studies included in the global brain volume meta-analysis only the study design itself could be considered in the comparability section.

The following section shows the exact rating scheme applied in our meta-analysis and the detailed evaluation of included studies for the global brain volume analysis (AN_{acute}, $AN_{short\text{-}rec}$ and $AN_{longer\text{-}rec}$) and the ALE meta-analyses:

Newcastle-Ottawa-Scale (NOS) – modified version

Note: A study can be awarded a maximum of one star for each numbered item within the *Selection* and *Outcome* categories. A maximum of two stars can be given for *Comparability*.

Selection (Maximum 4 stars)

1) *Is the case definition adequate/ ascertainment of exposure (i.e. anorexia nervosa condition)?*

a) Yes, diagnosed by clinical expert/ diagnostic criteria according to DSM or ICD met/ independent validation/ measurement tool *
b) Yes, e.g., record linkage or based on self reports
c) No description

2) *Representativeness of the cases*

a) Consecutive or obviously representative series of cases *
b) Potential for selection biases or not stated

3) *Selection of Controls*

a) Community controls *
b) Hospital controls
c) No description

4) *Definition of Controls*

a) No history of anorexia nervosa *
b) No mention of history of anorexia nervosa

<u>Comparability (Maximum 2 stars)</u>

1) *Comparability of cohorts on the basis of the design or analysis.* (Either cases and controls must be matched in the design and/or confounders must be adjusted for in the analysis. Statements of no differences between groups or that differences were not statistically significant are not sufficient for establishing comparability.)

a) Study controls for the most important factors (age and sex) *
b) Study controls for any additional potential confounder *

<u>Outcome (Maximum 3 stars)</u>

1) *Assessment of outcome (i.e., brain structure)*

a) Adequate measurement method applied (i.e., MRI scan of sufficiently good quality) *
b) No description

2) Analysis method (for estimation of outcome of interest)

a) Appropriate analysis method applied (to determine exact global volume score or regional structural brain volume changes) *
b) No description

3) *Accordance in outcome measurement and analysis for all cases and controls*

a) Same measurement <u>and</u> analysis method applied for all cases and controls *
b) Different measurement and/or analysis method applied for cases and controls
c) No description

NOS rating for studies included in the global brain volume meta-analysis, for patients with AN_{acute}:

Study	Case definition adequate	Representativeness of the cases	Selection of Controls	Definition of Controls	Comparability of cases and controls on the basis of the design or analysis	Assessment of outcome	Analysis method	Accordance in outcome measurement and analysis	Total NOS score
Amianto et al., 2013	*	*	*	*		*	*	*	7
Bär et al., 2015	*	*	*	*	*	*	*	*	8
Boghi et al., 2011	*	*		*	*	*	*	*	7
Bomba et al., 2013	*	*			*	*	*	*	6
Boto et al., 2017		*				*	*	*	4
Brooks et al., 2011	*	*	*	*		*	*	*	7
Burkert et al., 2015	*	*	*	*	*	*	*	*	8
Castro-Fornieles et al., 2009	*	*	*			*	*	*	6
Curzio et al., 2020	*	*		*	*	*	*		6
D'Agata et al., 2015	*	*	*	*		*	*	*	7
Doose et al., 2023	*	*	*	*	*	*	*	*	8
Favaro et al., 2015	*	*		*		*	*	*	6
Fonville et al., 2014	*	*	*	*	**	*	*	*	9
Frank et al., 2013a	*	*	*		*	*	*	*	7
Frank et al., 2013b	*	*	*			*	*	*	6
Friederich et al., 2012	*	*	*	*	*	*	*	*	8
Fujisawa et al., 2015	*		*		**	*	*	*	7
Gaudio et al., 2011	*	*		*		*	*	*	6
Gaudio et al., 2017	*	*	*	*	*	*	*	*	8
Geisler et al., 2022	*	*	*	*	*	*	*	*	8
Halls et al., 2022	*	*	*	*		*	*	*	7
Joos et al., 2010	*	*	*	*	*	*	*	*	8
Katzman et al., 1996	*	*		*		*	*	*	6
Kaufmann et al., 2020	*	*		*	**	*	*	*	8

**Khalsa et al., 2016**	*				*	*	*	*	5
**King et al., 2015**	*	*	*	*	*	*	*	*	8
**Kohmura et al., 2017**	*	*	*	*	*	*	*		7
**Lloyd et al., 2023**	*	*		*	*	*	*		6
**Mishima et al., 2021**	*	*	*	*	*	*	*	*	8
**Myrvang et al., 2018**	*	*	*	*	*	*	*		7
**Nickel et al., 2018**	*	*			**	*	*	*	7
**Olivo et al., 2018**	*	*	*	*	*	*	*	*	8
**Roberto et al., 2011**	*	*	*	*	**	*	*	*	9
**Scaife et al., 2017**	*			*		*	*	*	5
**Seitz et al., 2015**	*	*	*	*	*	*	*		7
**Suchan et al., 2010**	*			*	*	*	*	*	6
**Swayze et al., 2003**	*	*	*		*	*	*		6
**Via et al., 2014**	*	*	*		**	*	*	*	8
**Yu et al., 2024**	*	*	*	*		*	*		6
**Yue et al., 2018**	*		*	*		*	*	*	6

NOS rating for studies included in the global brain volume meta-analysis, for patients with $AN_{short\text{-}rec}$:

Study	Case definition adequate	Representativeness of the cases	Selection of Controls	Definition of Controls	Comparability of cases and controls on the basis of the design or analysis	Assessment of outcome	Analysis method	Accordance in outcome measurement and analysis	Total NOS score
Bomba et al., 2015	*	*			*	*	*	*	6
Brodrick et al., 2021	*	*		*		*	*	*	6
Castro-Fornieles et al., 2009	*	*	*			*	*	*	6
Frank et al., 2013a	*	*	*		*	*	*	*	7
Katzman et al., 1997	*	*		*	*	*	*	*	7
Kaufmann et al., 2020	*	*		*	**	*	*	*	8
Khalsa et al., 2016	*				*	*	*	*	5
Lázaro et al., 2013	*	*	*	*		*	*	*	7
Mainz et al., 2012	*	*	*	*	*	*	*	*	8
Roberto et al., 2011	*	*	*	*	**	*	*	*	9
Swayze et al., 2003	*	*	*		*	*	*		6

NOS rating for studies included in the global brain volume meta-analysis, for patients with $AN_{longer-rec}$:

Study	Case definition adequate	Representativeness of the cases	Selection of Controls	Definition of Controls	Comparability of cases and controls on the basis of the design or analysis	Assessment of outcome	Analysis method	Accordance in outcome measurement and analysis	Total NOS score
Bang et al., 2016	*	*	*	*	*	*	*	*	8
Favaro et al., 2015	*	*		*		*	*	*	6
Friederich et al., 2012	*	*	*	*	*	*	*	*	8
Joos et al., 2011			*	*		*	*	*	5
King et al., 2015	*	*	*	*	*	*	*	*	8
Lambe et al., 1997	*	*	*	*	*	*	*	*	8
Mühlau et al., 2007	*	*		*		*	*	*	6
Nickel et al., 2018	*	*			**	*	*	*	7
Oliva et al., 2020	*	*		*	*	*	*	*	7
Pfuhl et al., 2016	*	*	*	*	*	*	*	*	8
Scaife et al., 2017	*			*		*	*	*	5
Wagner et al., 2006	*			*		*	*	*	5
Zucker et al., 2017	*	*		*	**	*	*	*	8

NOS rating for studies included in the ALE meta-analyses:

Study	Case definition adequate	Representativeness of the cases	Selection of Controls	Definition of Controls	Comparability of cases and controls on the basis of the design or analysis	Assessment of outcome	Analysis method	Accordance in outcome measurement and analysis	Total NOS score
Amianto et al., 2013	*	*	*	*	**	*	*	*	9
Bär et al., 2015	*	*	*	*	*	*	*	*	8
Boghi et al., 2011	*	*		*	**	*	*	*	8
Bomba et al., 2015	*	*			*	*	*	*	6
Brooks et al., 2011	*	*	*	*	**	*	*	*	9
Cascino et al., 2020	*	*		*	*	*	*		6
Castro-Fornieles et al., 2021	*	*	*	*		*	*	*	7
Castro-Fornieles et al., 2009	*	*	*			*	*	*	6
Collantoni et al., 2024	*	*	*	*	*	*	*		7
D'Agata et al., 2015	*	*	*	*	**	*	*	*	9
De la Cruz et al., 2021	*	*		*	*	*	*	*	7
Favaro et al., 2015	*	*		*	**	*	*	*	8
Fonville et al., 2014	*	*	*	*	**	*	*	*	9
Frank et al., 2013a	*	*	*		**	*	*	*	8
Frank et al., 2013b	*	*	*		**	*	*	*	8
Friederich et al., 2012	*	*	*	*	**	*	*	*	9
Fujisawa et al., 2015	*		*		**	*	*	*	7
Gaudio et al., 2011	*	*		*	**	*	*	*	8
Gaudio et al., 2017	*	*	*	*	*	*	*	*	8
Joos et al., 2010	*	*	*	*	*	*	*	*	8
Kaufmann et al., 2020	*	*		*	**	*	*	*	8
Kohmura et al., 2017	*	*	*	*	**	*	*		8
Lenhart et al., 2022	*	*		*	**	*	*	*	8
Leppanen et al., 2019	*	*	*	*	*	*	*		7

**Martin Monzon et al., 2017**	*	*		*	**	*	*	*	8
**Mishima et al., 2021**	*	*	*	*	*	*	*	*	8
**Mühlau et al., 2007**	*	*		*	*	*	*	*	7
**Nickel et al., 2018**	*	*			**	*	*	*	7
**Oliva et al., 2020**	*	*		*	*	*	*	*	7
**Phillipou et al., 2018**	*	*	*	*	**	*	*	*	9
**Seitz et al., 2015**	*	*	*	*	*	*	*		7
**Suchan et al., 2010**	*			*	*	*	*	*	6
**Tose et al., 2024**	*	*		*	**	*	*		7
**Van Opstal et al., 2015**	*	*	*			*	*	*	6
**Yu et al., 2024**	*	*	*	*	**	*	*		8

5 References Supplement

Aghourian, M., Legault-Denis, C., Soucy, J.-P., Rosa-Neto, P., Gauthier, S., Kostikov, A., Gravel, P., & Bédard, M.-A. (2017). Quantification of brain cholinergic denervation in Alzheimer's disease using PET imaging with [18F]-FEOBV. *Molecular Psychiatry*, *22*(11), Article 11. https://doi.org/10.1038/mp.2017.183

Amianto, F., Caroppo, P., D'Agata, F., Spalatro, A., Lavagnino, L., Caglio, M., Righi, D., Bergui, M., Abbate-Daga, G., Rigardetto, R., Mortara, P., & Fassino, S. (2013). Brain volumetric abnormalities in patients with anorexia and bulimia nervosa: A voxel-based morphometry study. *Psychiatry Research*, *213*(3), 210–216. https://doi.org/10.1016/j.pscychresns.2013.03.010

Bang, L., Rø, Ø., & Endestad, T. (2016). Normal gray matter volumes in women recovered from anorexia nervosa: A voxel-based morphometry study. *BMC Psychiatry*, *16*. https://doi.org/10.1186/s12888-016-0856-z

Bär, K.-J., de la Cruz, F., Berger, S., Schultz, C. C., & Wagner, G. (2015). Structural and functional differences in the cingulate cortex relate to disease severity in anorexia nervosa. *Journal of Psychiatry & Neuroscience : JPN*, *40*(4), 269–279. https://doi.org/10.1503/jpn.140193

Beliveau, V., Ganz, M., Feng, L., Ozenne, B., Højgaard, L., Fisher, P. M., Svarer, C., Greve, D. N., & Knudsen, G. M. (2017). A High-Resolution *In Vivo* Atlas of the Human Brain's Serotonin System. *The Journal of Neuroscience*, *37*(1), 120–128. https://doi.org/10.1523/JNEUROSCI.2830-16.2016

Boghi, A., Sterpone, S., Sales, S., D'Agata, F., Bradac, G. B., Zullo, G., & Munno, D. (2011). In vivo evidence of global and focal brain alterations in anorexia nervosa. *Psychiatry Research*, *192*(3), 154–159. https://doi.org/10.1016/j.pscychresns.2010.12.008

Bomba, M., Riva, A., Morzenti, S., Grimaldi, M., Neri, F., & Nacinovich, R. (2015). Global and regional brain volumes normalization in weight-recovered adolescents with anorexia nervosa: Preliminary findings of a longitudinal voxel-based morphometry study. *Neuropsychiatric Disease and Treatment*, *11*, 637–645. https://doi.org/10.2147/NDT.S73239

Bomba, M., Riva, A., Veggo, F., Grimaldi, M., Morzenti, S., Neri, F., & Nacinovich, R. (2013). Impact of speed and magnitude of weight loss on the development of brain trophic changes in adolescents with anorexia nervosa: A case control study. *Italian Journal of Pediatrics*, *39*, 14. https://doi.org/10.1186/1824-7288-39-14

Boto, J., Gkinis, G., Roche, A., Kober, T., Maréchal, B., Ortiz, N., Lövblad, K.-O., Lazeyras, F., & Vargas, M. I. (2017). Evaluating anorexia-related brain atrophy using MP2RAGE-based morphometry. *European Radiology*, *27*(12), 5064–5072. https://doi.org/10.1007/s00330-017-4914-9

Brodrick, B. B., Adler-Neal, A. L., Palka, J. M., Mishra, V., Aslan, S., & McAdams, C. J. (2021). Structural brain differences in recovering and weight-recovered adult outpatient women with anorexia nervosa. *Journal of Eating Disorders*, *9*(1), 108. https://doi.org/10.1186/s40337-021-00466-w

Brooks, S. J., Barker, G. J., O'Daly, O. G., Brammer, M., Williams, S. C., Benedict, C., Schiöth, H. B., Treasure, J., & Campbell, I. C. (2011). Restraint of appetite and reduced regional brain volumes in anorexia nervosa: A voxel-based morphometric study. *BMC Psychiatry*, *11*, 179. https://doi.org/10.1186/1471-244X-11-179

Burkert, N. T., Koschutnig, K., Ebner, F., & Freidl, W. (2015). Structural hippocampal alterations, perceived stress, and coping deficiencies in patients with anorexia nervosa. *INTERNATIONAL JOURNAL OF EATING DISORDERS*, *48*(6), 670–676. https://doi.org/10.1002/eat.22397

Cascino, G., Canna, A., Monteleone, A. M., Russo, A. G., Prinster, A., Aiello, M., Esposito, F., Salle, F. D., & Monteleone, P. (2020). Cortical thickness, local gyrification index and fractal dimensionality in people with acute and recovered Anorexia Nervosa and in people with Bulimia Nervosa. *Psychiatry Research. Neuroimaging*, *299*, 111069. https://doi.org/10.1016/j.pscychresns.2020.111069

Castro-Fornieles, J., Bargalló, N., Lázaro, L., Andrés, S., Falcon, C., Plana, M. T., & Junqué, C. (2009). A cross-sectional and follow-up voxel-based morphometric MRI study in adolescent anorexia nervosa. *Journal of Psychiatric Research*, *43*(3), 331–340. https://doi.org/10.1016/j.jpsychires.2008.03.013

Castro-Fornieles, J., de la Serna, E., Calvo, A., Pariente, J., Andrés-Perpiña, S., Plana, M. T., Romero, S., Flamarique, I., Gárriz, M., & Bargalló, N. (2021). Cortical thickness 20 years after diagnosis of anorexia nervosa during adolescence. *European Archives of Psychiatry and Clinical Neuroscience*, *271*(6), 1133–1139. https://doi.org/10.1007/s00406-019-00992-4

Collantoni, E., Alberti, F., Dahmen, B., von Polier, G., Konrad, K., Herpertz-Dahlmann, B., Favaro, A., & Seitz, J. (2024). Intra-individual cortical networks in Anorexia Nervosa: Evidence from a longitudinal dataset. *European Eating Disorders Review : The Journal of the Eating Disorders Association*, *32*(2), 298–309. https://doi.org/10.1002/erv.3043

Curzio, O., Calderoni, S., Maestro, S., Rossi, G., Pasquale, C. F. D., Belmonti, V., Apicella, F., Muratori, F., & Retico, A. (2020). Lower gray matter volumes of frontal lobes and insula in adolescents with anorexia nervosa restricting type: Findings from a Brain Morphometry Study. *European Psychiatry*, *63*(1), e27. https://doi.org/10.1192/j.eurpsy.2020.19

D'Agata, F., Caroppo, P., Amianto, F., Spalatro, A., Caglio, M. M., Bergui, M., Lavagnino, L., Righi, D., Abbate-Daga, G., Pinessi, L., Mortara, P., & Fassino, S. (2015). Brain correlates of alexithymia in eating disorders: A voxel-based morphometry study. *Psychiatry and Clinical Neurosciences*, *69*(11), 708–716. https://doi.org/10.1111/pcn.12318

de la Cruz, F., Schumann, A., Suttkus, S., Helbing, N., Zopf, R., & Bär, K.-J. (2021). Cortical thinning and associated connectivity changes in patients with anorexia nervosa. *Translational Psychiatry*, *11*. https://doi.org/10.1038/s41398-021-01237-6

Ding, Y.-S., Singhal, T., Planeta-Wilson, B., Gallezot, J.-D., Nabulsi, N., Labaree, D., Ropchan, J., Henry, S., Williams, W., Carson, R. E., Neumeister, A., & Malison, R. T. (2010). PET imaging of the effects of age and cocaine on the norepinephrine transporter in the human brain using (S,S)-[11C]O-methylreboxetine and HRRT. *Synapse*, *64*(1), 30–38. https://doi.org/10.1002/syn.20696

Doose, A., Tam, F. I., Hellerhoff, I., King, J. A., Boehm, I., Gottloeber, K., Wahl, H., Werner, A., Raschke, F., Bartnik-Olson, B., Lin, A. P., Akgün, K., Roessner, V., Linn, J., & Ehrlich, S. (2023). Triangulating brain alterations in anorexia nervosa: A multimodal investigation of magnetic resonance spectroscopy, morphometry and blood-based biomarkers. *Translational Psychiatry*, *13*(1), 277. https://doi.org/10.1038/s41398-023-02580-6

Dukart, J., Holiga, Š., Chatham, C., Hawkins, P., Forsyth, A., McMillan, R., Myers, J., Lingford-Hughes, A. R., Nutt, D. J., Merlo-Pich, E., Risterucci, C., Boak, L., Umbricht, D., Schobel, S., Liu, T., Mehta, M. A., Zelaya, F. O., Williams, S. C., Brown, G., … Sambataro, F. (2018). Cerebral blood flow predicts differential neurotransmitter activity. *Scientific Reports*, *8*(1), Article 1. https://doi.org/10.1038/s41598-018-22444-0

Eickhoff, S. B., Bzdok, D., Laird, A. R., Kurth, F., & Fox, P. T. (2012). Activation likelihood estimation meta-analysis revisited. *NeuroImage*, *59*(3), 2349–2361. https://doi.org/10.1016/j.neuroimage.2011.09.017

Eickhoff, S., Laird, A., Grefkes, C., Wang, L., Zilles, K., & Fox, P. (2009). Coordinate-Based Activation Likelihood Estimation Meta-Analysis of Neuroimaging Data: A Random-Effects Approach Based on Empirical Estimates of Spatial Uncertainty. *Human brain mapping*, *30*, 2907–2926. https://doi.org/10.1002/hbm.20718

Favaro, A., Tenconi, E., Degortes, D., Manara, R., & Santonastaso, P. (2015). Gyrification brain abnormalities as predictors of outcome in anorexia nervosa. *Human Brain Mapping*, *36*(12), 5113–5122. https://doi.org/10.1002/hbm.22998

Fonville, L., Giampietro, V., Williams, S. C. R., Simmons, A., & Tchanturia, K. (2014). Alterations in brain structure in adults with anorexia nervosa and the impact of illness duration. *Psychological Medicine*, *44*(9), 1965–1975. https://doi.org/10.1017/S0033291713002389

Frank, G. K., Shott, M. E., Hagman, J. O., & Mittal, V. A. (2013a). Alterations in brain structures related to taste reward circuitry in ill and recovered anorexia nervosa and in bulimia nervosa. *The American Journal of Psychiatry*, *170*(10), 1152–1160. https://doi.org/10.1176/appi.ajp.2013.12101294

Frank, G. K., Shott, M. E., Hagman, J. O., & Yang, T. T. (2013b). Localized Brain Volume and White Matter Integrity Alterations in Adolescent Anorexia Nervosa. *Journal of the American Academy of Child and Adolescent Psychiatry*, *52*(10), 1066-1075.e5. https://doi.org/10.1016/j.jaac.2013.07.007

Friederich, H.-C., Walther, S., Bendszus, M., Biller, A., Thomann, P., Zeigermann, S., Katus, T., Brunner, R., Zastrow, A., & Herzog, W. (2012). Grey matter abnormalities within cortico-limbic-striatal circuits in acute and weight-restored anorexia nervosa patients. *NeuroImage*, *59*(2), 1106–1113. https://doi.org/10.1016/j.neuroimage.2011.09.042

Fujisawa, T. X., Yatsuga, C., Mabe, H., Yamada, E., Masuda, M., & Tomoda, A. (2015). Anorexia Nervosa during Adolescence Is Associated with Decreased Gray Matter Volume in the Inferior Frontal Gyrus. *PLoS ONE*, *10*(6). https://doi.org/10.1371/journal.pone.0128548

Gallezot, J.-D., Nabulsi, N., Neumeister, A., Planeta-Wilson, B., Williams, W. A., Singhal, T., Kim, S., Maguire, R. P., McCarthy, T., Frost, J. J., Huang, Y., Ding, Y.-S., & Carson, R. E. (2010). Kinetic Modeling of the Serotonin 5-HT1B Receptor Radioligand [11C]P943 in Humans. *Journal of Cerebral Blood Flow & Metabolism*, *30*(1), 196–210. https://doi.org/10.1038/jcbfm.2009.195

Gallezot, J.-D., Planeta, B., Nabulsi, N., Palumbo, D., Li, X., Liu, J., Rowinski, C., Chidsey, K., Labaree, D., Ropchan, J., Lin, S.-F., Sawant-Basak, A., McCarthy, T. J., Schmidt, A. W., Huang, Y., & Carson, R. E. (2017). Determination of receptor occupancy in the presence of mass dose: [11C]GSK189254 PET imaging of histamine H3 receptor occupancy by PF-03654746. *Journal of Cerebral Blood Flow & Metabolism*, *37*(3), 1095–1107. https://doi.org/10.1177/0271678X16650697

Galovic, M., Al-Diwani, A., Vivekananda, U., Torrealdea, F., Erlandsson, K., Fryer, T. D., Hong, Y. T., Thomas, B. A., McGinnity, C. J., Edmond, E., Sander, K., Årstad, E., Jelcic, I., Aigbirhio, F. I., Groves, A. M., Thielemans, K., Hutton, B., Hammers, A., Duncan, J. S., … Investigators, for the N. (2021). *In vivo NMDA receptor function in people with NMDA receptor antibody encephalitis* (S. 2021.12.04.21267226). medRxiv. https://doi.org/10.1101/2021.12.04.21267226

Gaudio, S., Nocchi, F., Franchin, T., Genovese, E., Cannatà, V., Longo, D., & Fariello, G. (2011). Gray matter decrease distribution in the early stages of Anorexia Nervosa restrictive type in adolescents. *Psychiatry Research*, *191*(1), 24–30. https://doi.org/10.1016/j.pscychresns.2010.06.007

Gaudio, S., Quattrocchi, C. C., Piervincenzi, C., Zobel, B. B., Montecchi, F. R., Dakanalis, A., Riva, G., & Carducci, F. (2017). White matter abnormalities in treatment-naive adolescents at the earliest stages of Anorexia Nervosa: A diffusion tensor imaging study. *Psychiatry Research. Neuroimaging*, *266*, 138–145. https://doi.org/10.1016/j.pscychresns.2017.06.011

Geisler, D., King, J. A., Bahnsen, K., Bernardoni, F., Doose, A., Müller, D. K., Marxen, M., Roessner, V., van den Heuvel, M., & Ehrlich, S. (2022). Altered White Matter Connectivity in Young Acutely Underweight Patients With Anorexia Nervosa. *Journal of the American Academy of Child and Adolescent Psychiatry*, *61*(2), 331–340. https://doi.org/10.1016/j.jaac.2021.04.019

Gómez, F. J. G., Huertas, I., Ramírez, J. A. L., & Solís, D. G. (2018). Elaboración de una plantilla de SPM para la normalización de imágenes de PET con 18F-DOPA. *Imagen Diagnóstica*, *9*(01), Article 01. https://doi.org/10.33588/imagendiagnostica.901.2

Halls, D., Leppanen, J., Kerr-Gaffney, J., Simic, M., Nicholls, D., Mandy, W., Williams, S., & Tchanturia, K. (2022). Examining the relationship between autistic spectrum disorder characteristics and structural brain differences seen in anorexia nervosa. *European Eating Disorders Review : The Journal of the Eating Disorders Association*, *30*(5), 459–473. https://doi.org/10.1002/erv.2910

Hillmer, A. T., Esterlis, I., Gallezot, J. D., Bois, F., Zheng, M. Q., Nabulsi, N., Lin, S. F., Papke, R. L., Huang, Y., Sabri, O., Carson, R. E., & Cosgrove, K. P. (2016). Imaging of cerebral α4β2* nicotinic acetylcholine receptors with (−)-[18F]Flubatine PET: Implementation of bolus plus constant infusion and sensitivity to acetylcholine in human brain. *NeuroImage*, *141*, 71–80. https://doi.org/10.1016/j.neuroimage.2016.07.026

Joos, A., Hartmann, A., Glauche, V., Perlov, E., Unterbrink, T., Saum, B., Tüscher, O., Tebartz van Elst, L., & Zeeck, A. (2011). Grey matter deficit in long-term recovered anorexia nervosa patients. *European Eating Disorders Review: The Journal of the Eating Disorders Association*, *19*(1), 59–63. https://doi.org/10.1002/erv.1060

Joos, A., Klöppel, S., Hartmann, A., Glauche, V., Tüscher, O., Perlov, E., Saum, B., Freyer, T., Zeeck, A., & Tebartz van Elst, L. (2010). Voxel-based morphometry in eating disorders: Correlation of psychopathology with grey matter volume. *Psychiatry Research*, *182*(2), 146–151. https://doi.org/10.1016/j.pscychresns.2010.02.004

Kaller, S., Rullmann, M., Patt, M., Becker, G.-A., Luthardt, J., Girbardt, J., Meyer, P. M., Werner, P., Barthel, H., Bresch, A., Fritz, T. H., Hesse, S., & Sabri, O. (2017). Test–retest measurements of dopamine D1-type receptors using simultaneous PET/MRI imaging. *European Journal of Nuclear Medicine and Molecular Imaging*, *44*(6), 1025–1032. https://doi.org/10.1007/s00259-017-3645-0

Kantonen, T., Karjalainen, T., Isojärvi, J., Nuutila, P., Tuisku, J., Rinne, J., Hietala, J., Kaasinen, V., Kalliokoski, K., Scheinin, H., Hirvonen, J., Vehtari, A., & Nummenmaa, L. (2020). Interindividual variability and lateralization of μ-opioid receptors in the human brain. *NeuroImage*, *217*, 116922. https://doi.org/10.1016/j.neuroimage.2020.116922

Katzman, D. K., Lambe, E. K., Mikulis, D. J., Ridgley, J. N., Goldbloom, D. S., & Zipursky, R. B. (1996). Cerebral gray matter and white matter volume deficits in adolescent girls with anorexia nervosa. *The Journal of Pediatrics*, *129*(6), 794–803. https://doi.org/10.1016/s0022-3476(96)70021-5

Katzman, D. K., Zipursky, R. B., Lambe, E. K., & Mikulis, D. J. (1997). A longitudinal magnetic resonance imaging study of brain changes in adolescents with anorexia nervosa. *Archives of Pediatrics & Adolescent Medicine*, *151*(8), 793–797. https://doi.org/10.1001/archpedi.1997.02170450043006

Kaufmann, L.-K., Hänggi, J., Jäncke, L., Baur, V., Piccirelli, M., Kollias, S., Schnyder, U., Martin-Soelch, C., & Milos, G. (2020). Age influences structural brain restoration during weight gain therapy in anorexia nervosa. *Translational Psychiatry*, *10*. https://doi.org/10.1038/s41398-020-0809-7

Kaulen, N., Rajkumar, R., Régio Brambilla, C., Mauler, J., Ramkiran, S., Orth, L., Sbaihat, H., Lang, M., Wyss, C., Rota Kops, E., Scheins, J., Neumaier, B., Ermert, J., Herzog, H., Langen, K.-J., Lerche, C., Shah, N. J., Veselinović, T., & Neuner, I. (2022). mGluR5 and GABAA receptor-specific parametric PET atlas construction—PET/MR data processing pipeline, validation, and application. *Human Brain Mapping*, *43*(7), 2148–2163. https://doi.org/10.1002/hbm.25778

Khalsa, S. S., Kumar, R., Patel, V., Strober, M., & Feusner, J. D. (2016). Mammillary Body Volume Abnormalities in Anorexia Nervosa. *The International Journal of Eating Disorders*, *49*(10), 920–929. https://doi.org/10.1002/eat.22573

King, J. A., Geisler, D., Ritschel, F., Boehm, I., Seidel, M., Roschinski, B., Soltwedel, L., Zwipp, J., Pfuhl, G., Marxen, M., Roessner, V., & Ehrlich, S. (2015). Global Cortical Thinning in Acute Anorexia Nervosa Normalizes Following Long-Term Weight Restoration. *Biological Psychiatry*, *77*(7), 624–632. https://doi.org/10.1016/j.biopsych.2014.09.005

Kohmura, K., Adachi, Y., Tanaka, S., Katayama, H., Imaeda, M., Kawano, N., Nishioka, K., Ando, M., Iidaka, T., & Ozaki, N. (2017). Regional decrease in gray matter volume is related to body dissatisfaction in anorexia nervosa. *Psychiatry Research. Neuroimaging*, *267*, 51–58. https://doi.org/10.1016/j.pscychresns.2017.07.004

Lambe, E. K., Katzman, D. K., Mikulis, D. J., Kennedy, S. H., & Zipursky, R. B. (1997). Cerebral gray matter volume deficits after weight recovery from anorexia nervosa. *Archives of General Psychiatry*, *54*(6), 537–542. https://doi.org/10.1001/archpsyc.1997.01830180055006

Lancaster, J. L., Tordesillas-Gutiérrez, D., Martinez, M., Salinas, F., Evans, A., Zilles, K., Mazziotta, J. C., & Fox, P. T. (2007). Bias between MNI and Talairach coordinates analyzed using the ICBM-152 brain template. *Human Brain Mapping*, *28*(11), 1194–1205. https://doi.org/10.1002/hbm.20345

Lázaro, L., Andrés, S., Calvo, A., Cullell, C., Moreno, E., Plana, M. T., Falcón, C., Bargalló, N., & Castro-Fornieles, J. (2013). Normal gray and white matter volume after weight restoration in adolescents with anorexia nervosa. *The International Journal of Eating Disorders*, *46*(8), 841–848. https://doi.org/10.1002/eat.22161

Lenhart, L., Gander, M., Steiger, R., Dabkowska-Mika, A., Mangesius, S., Haid-Stecher, N., Fuchs, M., Buchheim, A., Sevecke, K., & Gizewski, E. R. (2022). Attachment status is associated with grey matter recovery in adolescent anorexia nervosa: Findings from a longitudinal study. *The European Journal of Neuroscience*, *55*(5), 1373–1387. https://doi.org/10.1111/ejn.15614

Leppanen, J., Sedgewick, F., Cardi, V., Treasure, J., & Tchanturia, K. (2019). Cortical morphometry in anorexia nervosa: An out-of-sample replication study. *European Eating Disorders Review*, *27*(5), 507–520. https://doi.org/10.1002/erv.2686

Lloyd, E. C., Foerde, K. E., Muratore, A. F., Aw, N., Semanek, D., Steinglass, J. E., & Posner, J. (2023). Large-Scale Exploration of Whole-Brain Structural Connectivity in Anorexia Nervosa: Alterations in the Connectivity of Frontal and Subcortical Networks. *Biological Psychiatry. Cognitive Neuroscience and Neuroimaging*, *8*(8), 864–873. https://doi.org/10.1016/j.bpsc.2022.06.002

Lukow, P. B., Martins, D., Veronese, M., Vernon, A. C., McGuire, P., Turkheimer, F. E., & Modinos, G. (2022). Cellular and molecular signatures of in vivo imaging measures of GABAergic neurotransmission in the human brain. *Communications Biology*, *5*(1), Article 1. https://doi.org/10.1038/s42003-022-03268-1

Mainz, V., Schulte-Rüther, M., Fink, G. R., Herpertz-Dahlmann, B., & Konrad, K. (2012). Structural brain abnormalities in adolescent anorexia nervosa before and after weight recovery and associated hormonal changes. *Psychosomatic Medicine*, *74*(6), 574–582. https://doi.org/10.1097/PSY.0b013e31824ef10e

Martin Monzon, B., Henderson, L. A., Madden, S., Macefield, V. G., Touyz, S., Kohn, M. R., Clarke, S., Foroughi, N., & Hay, P. (2017). Grey matter volume in adolescents with anorexia nervosa and associated eating disorder symptoms. *The European Journal of Neuroscience*, *46*(7), 2297–2307. https://doi.org/10.1111/ejn.13659

Mishima, R., Isobe, M., Noda, T., Tose, K., Kawabata, M., Noma, S., & Murai, T. (2021). Structural brain changes in severe and enduring anorexia nervosa: A multimodal magnetic resonance imaging study of gray matter volume, cortical thickness, and white matter integrity. *Psychiatry Research. Neuroimaging*, *318*, 111393. https://doi.org/10.1016/j.pscychresns.2021.111393

Mühlau, M., Gaser, C., Ilg, R., Conrad, B., Leibl, C., Cebulla, M. H., Backmund, H., Gerlinghoff, M., Lommer, P., Schnebel, A., Wohlschläger, A. M., Zimmer, C., & Nunnemann, S. (2007). Gray matter decrease of the anterior cingulate cortex in anorexia nervosa. *The American Journal of Psychiatry*, *164*(12), 1850–1857. https://doi.org/10.1176/appi.ajp.2007.06111861

Myrvang, A. D., Vangberg, T. R., Stedal, K., Rø, Ø., Endestad, T., Rosenvinge, J. H., & Aslaksen, P. M. (2018). Hippocampal subfields in adolescent anorexia nervosa. *Psychiatry Research. Neuroimaging*, *282*, 24–30. https://doi.org/10.1016/j.pscychresns.2018.10.007

Naganawa, M., Nabulsi, N., Henry, S., Matuskey, D., Lin, S.-F., Slieker, L., Schwarz, A. J., Kant, N., Jesudason, C., Ruley, K., Navarro, A., Gao, H., Ropchan, J., Labaree, D., Carson, R. E., & Huang, Y. (2021). First-in-Human Assessment of 11C-LSN3172176, an M1 Muscarinic Acetylcholine Receptor PET Radiotracer. *Journal of Nuclear Medicine*, *62*(4), 553–560. https://doi.org/10.2967/jnumed.120.246967

Nickel, K., Joos, A., Tebartz van Elst, L., Matthis, J., Holovics, L., Endres, D., Zeeck, A., Hartmann, A., Tüscher, O., & Maier, S. (2018). Recovery of cortical volume and thickness after remission from acute anorexia nervosa. *The International Journal of Eating Disorders*, *51*(9), 1056–1069. https://doi.org/10.1002/eat.22918

Normandin, M. D., Zheng, M.-Q., Lin, K.-S., Mason, N. S., Lin, S.-F., Ropchan, J., Labaree, D., Henry, S., Williams, W. A., Carson, R. E., Neumeister, A., & Huang, Y. (2015). Imaging the Cannabinoid CB1 Receptor in Humans with [11C] OMAR: Assessment of Kinetic Analysis Methods, Test–Retest Reproducibility, and Gender Differences. *Journal of Cerebral Blood Flow & Metabolism*, *35*(8), 1313–1322. https://doi.org/10.1038/jcbfm.2015.46

Oliva, R., Baiano, M., Salvo, P., Cereser, L., Castiello, U., & Begliomini, C. (2020). Metacognition in individuals recovered from anorexia nervosa: A voxel-based morphometry study. *Psychiatry Research. Neuroimaging*, *304*, 111138. https://doi.org/10.1016/j.pscychresns.2020.111138

Olivo, G., Solstrand Dahlberg, L., Wiemerslage, L., Swenne, I., Zhukovsky, C., Salonen-Ros, H., Larsson, E.-M., Gaudio, S., Brooks, S. J., & Schiöth, H. B. (2018). Atypical anorexia nervosa is not related to brain structural changes in newly diagnosed adolescent patients. *The International Journal of Eating Disorders*, *51*(1), 39–45. https://doi.org/10.1002/eat.22805

Pfuhl, G., King, J. A., Geisler, D., Roschinski, B., Ritschel, F., Seidel, M., Bernardoni, F., Müller, D. K., White, T., Roessner, V., & Ehrlich, S. (2016). Preserved white matter microstructure in young patients with anorexia nervosa? *Human Brain Mapping*, *37*(11), 4069–4083. https://doi.org/10.1002/hbm.23296

Phillipou, A., Rossell, S. L., Gurvich, C., Castle, D. J., Abel, L. A., Nibbs, R. G., & Hughes, M. E. (2018). Differences in regional grey matter volumes in currently ill patients with anorexia nervosa. *The European Journal of Neuroscience*, *47*(2), 177–183. https://doi.org/10.1111/ejn.13793

Radhakrishnan, R., Nabulsi, N., Gaiser, E., Gallezot, J.-D., Henry, S., Planeta, B., Lin, S., Ropchan, J., Williams, W., Morris, E., D’Souza, D. C., Huang, Y., Carson, R. E., & Matuskey, D. (2018). Age-Related Change in 5-HT6 Receptor Availability in Healthy Male Volunteers Measured with 11C-GSK215083 PET. *Journal of Nuclear Medicine*, *59*(9), 1445–1450. https://doi.org/10.2967/jnumed.117.206516

Roberto, C. A., Mayer, L. E. S., Brickman, A. M., Barnes, A., Muraskin, J., Yeung, L.-K., Steffener, J., Sy, M., Hirsch, J., Stern, Y., & Walsh, B. T. (2011). Brain tissue volume changes following weight gain in adults with anorexia nervosa. *The International Journal of Eating Disorders*, *44*(5), 406–411. https://doi.org/10.1002/eat.20840

Sandiego, C. M., Gallezot, J.-D., Lim, K., Ropchan, J., Lin, S., Gao, H., Morris, E. D., & Cosgrove, K. P. (2015). Reference Region Modeling Approaches for Amphetamine Challenge Studies with [11C]FLB 457 and PET. *Journal of Cerebral Blood Flow & Metabolism*, *35*(4), 623–629. https://doi.org/10.1038/jcbfm.2014.237

Scaife, J. C., Godier, L. R., Filippini, N., Harmer, C. J., & Park, R. J. (2017). Reduced Resting-State Functional Connectivity in Current and Recovered Restrictive Anorexia Nervosa. *Frontiers in Psychiatry*, *8*, 30. https://doi.org/10.3389/fpsyt.2017.00030

Seitz, J., Walter, M., Mainz, V., Herpertz-Dahlmann, B., Konrad, K., & von Polier, G. (2015). Brain volume reduction predicts weight development in adolescent patients with anorexia nervosa. *Journal of Psychiatric Research*, *68*, 228–237. https://doi.org/10.1016/j.jpsychires.2015.06.019

Smart, K., Cox, S. M. L., Scala, S. G., Tippler, M., Jaworska, N., Boivin, M., Séguin, J. R., Benkelfat, C., & Leyton, M. (2019). Sex differences in [11C]ABP688 binding: A positron emission tomography study of mGlu5 receptors. *European Journal of Nuclear Medicine and Molecular Imaging*, *46*(5), 1179–1183. https://doi.org/10.1007/s00259-018-4252-4

Suchan, B., Busch, M., Schulte, D., Grönemeyer, D., Grönermeyer, D., Herpertz, S., & Vocks, S. (2010). Reduction of gray matter density in the extrastriate body area in women with anorexia nervosa. *Behavioural Brain Research*, *206*(1), 63–67. https://doi.org/10.1016/j.bbr.2009.08.035

Swayze, V. W., Andersen, A. E., Andreasen, N. C., Arndt, S., Sato, Y., & Ziebell, S. (2003). Brain tissue volume segmentation in patients with anorexia nervosa before and after weight normalization. *The International Journal of Eating Disorders*, *33*(1), 33–44. https://doi.org/10.1002/eat.10111

Tose, K., Takamura, T., Isobe, M., Hirano, Y., Sato, Y., Kodama, N., Yoshihara, K., Maikusa, N., Moriguchi, Y., Noda, T., Mishima, R., Kawabata, M., Noma, S., Takakura, S., Gondo, M., Kakeda, S., Takahashi, M., Ide, S., Adachi, H., … Sekiguchi, A. (2024). Systematic reduction of gray matter volume in anorexia nervosa, but relative enlargement with clinical symptoms in the prefrontal and posterior insular cortices: A multicenter neuroimaging study. *Molecular Psychiatry*, *29*(4), 891–901. https://doi.org/10.1038/s41380-023-02378-4

van Opstal, A. M., Westerink, A. M., Teeuwisse, W. M., van der Geest, M. A. M., van Furth, E. F., & van der Grond, J. (2015). Hypothalamic BOLD response to glucose intake and hypothalamic volume are similar in anorexia nervosa and healthy control subjects. *Frontiers in Neuroscience*, *9*, 159. https://doi.org/10.3389/fnins.2015.00159

Via, E., Zalesky, A., Sánchez, I., Forcano, L., Harrison, B. J., Pujol, J., Fernández-Aranda, F., Menchón, J. M., Soriano-Mas, C., Cardoner, N., & Fornito, A. (2014). Disruption of brain white matter microstructure in women with anorexia nervosa. *Journal of Psychiatry & Neuroscience: JPN*, *39*(6), 367–375. https://doi.org/10.1503/jpn.130135

Wagner, A., Greer, P., Bailer, U. F., Frank, G. K., Henry, S. E., Putnam, K., Meltzer, C. C., Ziolko, S. K., Hoge, J., McConaha, C., & Kaye, W. H. (2006). Normal brain tissue volumes after long-term recovery in anorexia and bulimia nervosa. *Biological Psychiatry*, *59*(3), 291–293. https://doi.org/10.1016/j.biopsych.2005.06.014

Wells, G., Shea, B., O'Connell, D., Peterson, J., Welch, V., Losos, M., & Tugwell, P. *The Newcastle-Ottawa Scale (NOS) for assessing the quality of nonrandomised studies in meta-analyses*. Ottawa Hospital Research Institute. Retrieved July 24, 2024. Available from: https://www.ohri.ca/programs/clinical_epidemiology/oxford.asp

Yu, X., Robinson, L., Bobou, M., Zhang, Z., Banaschewski, T., Barker, G. J., Bokde, A. L. W., Flor, H., Grigis, A., Garavan, H., Gowland, P., Heinz, A., Brühl, R., Martinot, J.-L., Paillère Martinot, M.-L., Artiges, E., Nees, F., Orfanos, D. P., Lemaître, H., … Desrivières, S. (2024). Multimodal investigations of structural and functional brain alterations in anorexia and bulimia nervosa and their relationships to psychopathology. *Biological Psychiatry*, S0006-3223(24)01759-1. https://doi.org/10.1016/j.biopsych.2024.11.008

Yue, L., Wang, Y., Kaye, W. H., Kang, Q., Huang, J.-B., Cheung, E. F. C., Xiao, S.-F., Wang, Z., Chen, J., & Chan, R. C. K. (2018). Structural alterations in the caudate nucleus and precuneus in un-medicated anorexia nervosa patients. *Psychiatry Research. Neuroimaging*, *281*, 12–18. https://doi.org/10.1016/j.pscychresns.2018.08.009

Zucker, N. L., Kragel, P. A., Wagner, H. R., Keeling, L., Mayer, E., Wang, J., Kang, M. S., Merwin, R., Simmons, W. K., & LaBar, K. S. (2017). The Clinical Significance of Posterior Insular Volume in Adolescent Anorexia Nervosa. *Psychosomatic Medicine*, *79*(9), 1025–1035. https://doi.org/10.1097/PSY.0000000000000510
